# Monkeypox virus 2022, gene heterogeneity and protein polymorphism

**DOI:** 10.1038/s41392-023-01540-2

**Published:** 2023-07-17

**Authors:** Yanjiao Li, Jingjing Hou, Zhong Sun, Jingjing Hu, Karuppiah Thilakavathy, Yuxi Wang, Zhongjun Shao, Yihan Lu, Weibing Wang, Chenglong Xiong

**Affiliations:** 1 Department of Epidemiology, School of Public Health, Fudan University; Key Laboratory of Public Health Safety, Ministry of Education, 200032 Shanghai, China; 2 Department of Pulmonary and Critical Care Medicine, Tongji Hospital, School of Medicine, Tongji University, 200065 Shanghai, China; 3 Department of Biomedical Sciences, Faculty of Medicine and Health Sciences, Universiti Putra Malaysia, Serdang, 43400 Malaysia; 4 Department of Epidemiology, Ministry of Education Key Lab of Hazard Assessment and Control in Special Operational Environment, School of Public Health, Air Force Medical University, 710068 Xi’an, China

**Keywords:** Microbiology, Infectious diseases

**Dear Editor**,

Human monkeypox (MPX), an endemic disease in equatorial Africa,^1^ has been causing outbreaks in non-endemic countries since early May 2022. Interestingly, many confirmed cases have no history of travel to Africa. This is the first time many MPX cases have been reported concurrently in both non-endemic and endemic countries in disparate geographical areas.^2^ In response, we have analyzed 27 genes or sequences from 643 full-length human monkeypox virus (MPXV) genomes collected after January 1 and submitted by August 7, 2022 (Supplementary Table 1).

We divided these 643 genomes into 24 clusters with a 95% identity threshold (Supplementary Table 2). The phylogenetic trees of the 26 genes or sequences (D14L was not detected in any MPXVs 2022) can be categorized into four types (Extend data).

The first type is the two-branch type, including C10L, Ckbp, CrmB, K1L, O1L_trctd, Rep2, and V-slfn genes. In this type, five clusters represented by hMpxV/India/KL-ICMR-16-5316-553/2022, hMpxV/Thailand/NIC PKT-M1/2022, hMpxV/Thailand/NIC-74/2022, hMpxV/USA/FL-DHCPPCDC-001/2022 and hMpxV/USA/VA-DHCPPCDC-002 converge as sub-clade I, while the remaining clusters form sub-clade II (Supplementary Fig. 1a). Both sub-clades originated in Nigeria in 2017, with sub-clade I potentially emerging earlier. Between 2018 and 2021, it was inadvertently imported as human cases in Britain, the United States, Israel, Singapore, and Turkey.^3^ However, the epidemic areas of sub-clade I in 2022 appear to be relatively limited, mainly in the United States, India, and Thailand. Nigeria, the origin of both sub-clades, established many sub-clade I strains in 2017 but none in 2022. Currently, sub-clade II strains are circulating globally (Supplementary Fig. 2).

The second type is the two-branch-plus type, which includes the A46R and B5R genes. These represent variations of the two-branch type, where the five aforementioned clusters still converge into sub-clade I. However, an additional branch emerges from sub-clade II (Supplementary Fig. 1b).

The third type is the one-branch type, which groups all the 2022 endemic clusters together compared to the reference sequences, such as the E3L gene (Supplementary Fig. 1c). However, in most genes of this type, there are still one or two clusters belonging to sub-clade I that clearly show the distance from other clusters, for example, the B13R, B19R, C7L, D11L, N1R, and T4 genes (Supplementary Fig. 1d). There are also clusters belonging to sub-clade II (e.g., hMpxV/Hungary/NBL-001/2022) that show significant distance from others, such as the K4L gene (Supplementary Fig. 1e).

The fourth type is the irregular type, which includes nine genes: B14R, C1L, C6R, D7L, F1L, Hemagglutinin, N2L, O1L, and P1L (Fig. 1a, Supplementary Fig. 1f). In this phylogenetic tree type, most clusters still display properties of either one-branch or two-branch types. However, in contrast to the majority that can be converged, the prominent branches no longer belong to the clusters within sub-clade I, but rather to those within sub-clade II, the most common of which is hMpxV/Hungary/NBL-001/2022.

**Fig. 1:**
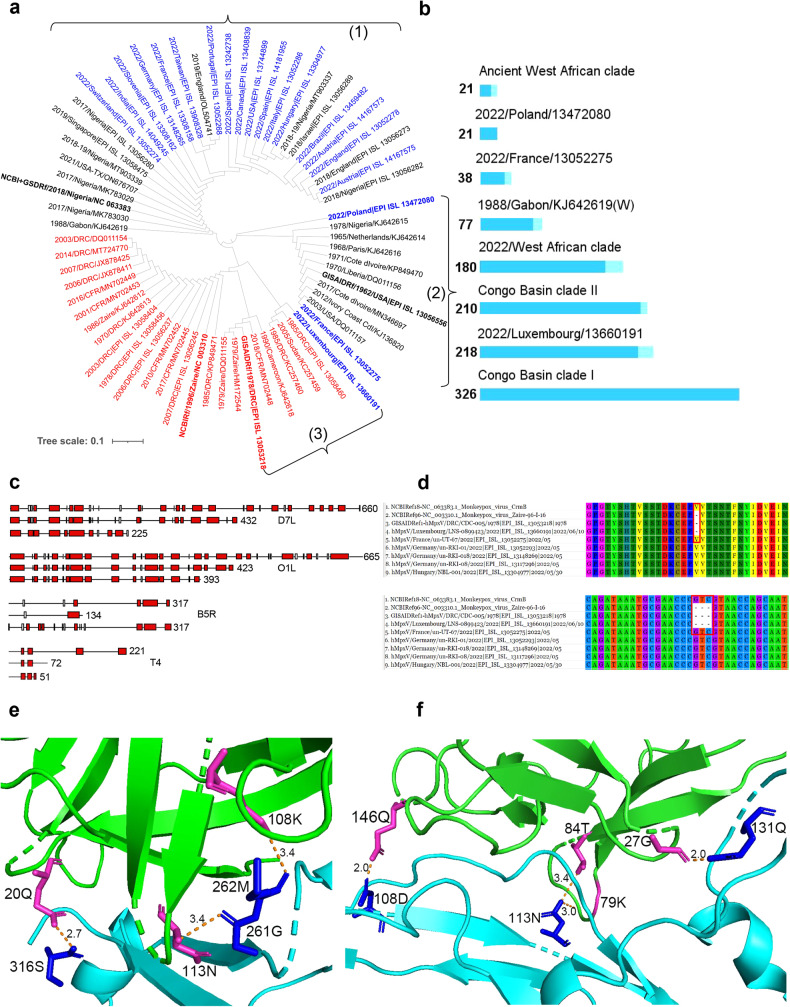
Gene heterogeneity and protein polymorphism of Monkeypox viruses in 2022. **a** The phylogenetic tree of B14R genes of MPXVs. Strains in red belong to the Congo Basin clade; those in black are from the West African clade before 2022, while the ones in blue are the current strains from 2022. Brace (2) includes strains from the ancient West African clade, while (1) includes strains from after 2017, primarily responsible for the current epidemic. In the Congo Basin clade, strains within brace (3) belong to sub-CBC II and those outside brace (3) belong to sub-CBC I. The hMpxV/Luxembourg/LNS-0899423/2022 strain from the West African clade shows similarity to the Congo Basin clade. **b** The polymorphism in the length of proteins encoded by B14R genes of monkeypox viruses (MPXVs). Blue bars indicate a consistency of more than 90%, while cyan bars a indicate consistency of less than 90%. In the Congo Basin clade, MPXVs express two product lengths; one is 326 AA (sub-CBC I, or Congo Basin clade I) and the other is 206-210 AA (sub-CBC II, or Congo Basin clade II). The others belong to the West African clade. **c** The proteins encoded by D7L, O1L, B5R, and T4 genes of MPXVs. In each of them, the first is the standard length of the protein, while the others are its variants. Proteins are shown as α-helix domains (according to the Gamier-Robson method) here. **d** The crmB gene (below) and its encoding protein (upper) of hMpxV/Luxembourg/LNS-0899423/2022. This strain is from the West African clade but displays an interesting hallmark belonging to the Congo Basin clade. **e**, **f** The complexities of B14R proteins and their ligands. In most Congo Basin clade isolates, three hydrogen bonds are formed via 261G, 262M, and 316S to the ligand IL-1β’s 113N, 108K, and 20Q, with bond lengths of 3.4 Å, 3.4 Å, and 2.7 Å respectively (**e**). In most current MPXVs 2022, four hydrogen bonds are formed via the 108D, 131Q, and 113N (twice) to the ligand IL-1β’s 146Q, 27G, and 79K and 84T, with the bond lengths of 2.0 Å, 2.0 Å, 3.0 Å, and 3.4 Å respectively (**f**). The cyan ribbon and blue amino acid residues, and the green ribbon and red amino acid residues represent the B14R proteins and the IL-1β ligands, respectively. The numbers denote the length values of hydrogen bonds

The complexity of the phylogenetic tree reflects the gene heterogeneity of the MPXVs 2022. This suggests that the current human MPX outbreak might be more complicated than anticipated. First, MPXVs 2022 may have been circulating in humans much earlier than May 2022. Studies concur that the MPXVs 2022 originated in Nigeria in 2017.^4^ It is plausible that the virus has been transmitting and circulating in humans at a low intensity since then, and genes susceptible to selective pressure have continuously accumulated variations. This means that some genes are more vulnerable to host or environmental pressure due to the properties of the proteins they encode, and these genes warrant more attention when using vaccines or antibodies to prevent human MPX. Second, this phenomenon might indicate the presence of yet undiscovered animal hosts or vectors in the environment, which could function as the natural reservoirs for monkeypox virus, consistently and stably maintaining the virus with ancestral genomic signatures. When conditions are favorable, this could result in cross-species transmission from the hosts to humans. In either case, long-term and timely surveillance of multiple animal species is vital for comprehending the origin, variation, and transmission of MPXVs. For the population infected with MPXV 2022 during this outbreak, it is essential to perform a differential diagnosis to determine whether the infecting pathogen possesses specific virulence-related genes and to ensure more effective treatment and better prognosis for patients.

Due to the early appearance of stop codons, the proteins encoded by the B14R, D7L, O1L, B5R, and T4 genes of MPXVs 2022 can be truncated into different lengths, demonstrating length polymorphism (Fig. 1b, c, Extend data). Although not supported by other contemporary MPXVs 2022, hMpxV/Luxembourg/LNS-0899423/2022 (EPI_13660191) displays more similarity to the virulent Congo Basin clade than to the mild West African clade. The protein encoded by its O1L gene has 423 amino acid residues (AA) (Fig. 1c), and in the crmB gene, a GTC deletion at positions 511-513nt results in a *valine* deletion at position 171 AA (Fig. 1d). This suggests that transitional strains with varying virulence may exist between the Congo Basin and West African clades.

In this study, we examined five virulence genes, including B14R, B19R, C7L, D14L, and T4. B19R and C7L are historically shared by both the West African and the Congo Basin clades, while the others were absent in the ancient West African strains.^5^ These genes play a crucial role in determining the virulence of MPXVs. D14L was not detected in any of the MPXVs 2022 genomes. Interestingly, functional protein products were identified in the B14R and T4 genes of these strains.

The B14R gene encodes an apoptotic regulator, interleukin-1β (IL-1β) binding protein, which binds to the IL-1β ligand to activate the corresponding signaling pathway and initiate host cell apoptosis.^6^ The B14R gene of the current MPXVs 2022 is markedly distinct from the virulent strains of the Congo Basin clade due to the presence of “AT” repeats with varying lengths in their ORFs (Extend data). The polymorphism in the length of the proteins encoded is noteworthy. In the Congo Basin clade, MPXVs can express two product lengths: 326 AA (referred to as sub-CBC I) and 206-210 AA (referred to as sub-CBC II). Before 2017, except for a 1988 Gabon strain (KJ642619) capable of expressing a protein up to 77 AA, none of the ancient West African strains could express a sufficiently long protein, with translation being halted at 21 AA by a series of consecutive stop codons. However, almost all MPXVs 2022 express proteins up to 180 AA. Only one Polish strain (EPI_13472080) and one French strain (EPI_13052275) still retain the characteristics of the ancient West African strains. The aforementioned hMpxV/Luxembourg/LNS-0899423/2022 strain again displays similarity to the Congo Basin clade due to its protein reaching up to 218 AA, even exceeding the 206-210 AA of sub-CBC II strains (Fig. 1b). Spatial modeling and receptor-ligand docking analysis reveal that despite the high spatial structural similarity between the full-length 326 AA receptors encoded by the sub-CBC I strain and the 180 AA encoded by MPXV 2022 (root mean square deviation (RMSD) value is 0.187), the truncated latter exhibits more efficient binding to IL-1β ligands through more hydrogen bonds (3 vs 4) (Fig. 1e, f) and lower binding free energy (ΔG_bind_, −2.36 KJ/mol vs −3.14 KJ/mol). This suggests that the current West African clade MPXVs 2022 may bind to the IL-1β more efficiently.

The T4 gene also encodes an apoptotic regulator and is traditionally viewed as another gene determining MPXV virulence, as the full-length protein (221 AA) it encodes is precisely expressed only in the virulent Congo Basin clade. In this study, the protein encoded by the T4 gene was found to have three different lengths. In the ancient mild West African clade, it encodes a protein with only 72 AA. In several strains isolated from the USA in 2003, it even encoded a 51 AA protein. However, almost all of the West African clade strains of the current MPXVs 2022 express the standard lengths of 221 AA, akin to the virulent Congo Basin clade (Fig. 1c). Full-length T4 was present in Nigerian isolates circulating as early as 2017.

These two genes of the MPXVs 2022 and their functional proteins may influence virulence and human-to-human transmission capacity, potentially responsible for the unprecedented expansions of infected populations and epidemic areas. In order to discover the trends of virus virulence and human-to-human transmission capacity in advance, and predict the effectiveness of existing MPXV vaccines (or smallpox vaccines) in providing protection, it is essential to not only consider the variation and evolution of MPXV but also closely monitor transmission in potential hosts or vectors.

## Supplementary information

Table S1

Table S2

Supplementary Fig.1

Supplementary Fig.2

Supplementary_Materials_8463R1

Data S1

Data S2

Data S3

Data S4

Data S5

Data S6

Data S7

## Data Availability

All data and materials are presented either in the main manuscript or in the Supplementary materials and are available upon request.

## References

[1] Lopera JG, Falendysz EA, Rocke TE, Osorio JE (2015). Attenuation of monkeypox virus by deletion of genomic regions. Virology.

[2] ECDC-WHO. *Joint ECDC-WHO Regional Office for Europe Monkeypox Surveillance Bulletin*. https://monkeypoxreport.ecdc.europa.eu/.

[3] Vaughan A (2020). Human-to-human transmission of monkeypox virus, United Kingdom, October 2018. Emerg. Infect. Dis..

[4] Mauldin MR (2022). Exportation of monkeypox virus from the African continent. J. Infect. Dis..

[5] Chen N (2005). Virulence differences between monkeypox virus isolates from West Africa and the Congo basin. Virology.

[6] Alcamí A, Smith GL (1996). A mechanism for the inhibition of fever by a virus. Proc. Natl Acad. Sci. USA.

