# Supplementary material for: Monkeypox virus 2022, gene heterogeneity and protein polymorphism
Source: Signal Transduct Target Ther. 2023 Jul 17;8:278. doi: 10.1038/s41392-023-01540-2 (PMC10352349; doi:10.1038/s41392-023-01540-2)
Supplement: Supplementary file 1 — Table S1 [file 41392_2023_1540_MOESM1_ESM.docx]

**Table S1. Genes and sequences used in this study**

| **Name** | **CDS** | **VACV-WR^a^** | **VACV-Cop^b^** | **Main Function** | **Reference** |
| --- | --- | --- | --- | --- | --- |
| A46R | 149809..150531 | A46R | A45R | Superoxide dismutase-like | 5 |
| B13R | 172150..172599 | B13R | C16L, B15R | Serine protease inhibitor (SPI-2/CrmA) | 7 |
| B19R | 174532..175587 | B16R | B19R | IFN-α/β binding protein | 5 |
| B5R | 165122..166075 | B5R | B4R | Extracellular enveloped virus (EEV) complement control protein | 8,9 |
| C10L | Com^*^(17878..18828) | D13L | C10L | IL-1 receptor antagonist | 9,10 |
| C1L | Com(19361..20005) | D19L | C1L | Bcl-2-like protein | 11 |
| C6R | 28091..28540 | C6R | K7R | Putative TLR signaling inhibitor | 5 |
| C7L | Com(15925..16377) | D10L | C7L | Host range; Type 1 IFN inhibitor | 5 |
| Ckbp | 195635..196375 | J3R | C23L, B29R | CC-chemokine binding protein | 5,10,12 |
| CrmB | 194459..195508 | J2R | C22L | Secreted TNF binding protein | 5 |
| D11L | Com(16602..17069) | D11L | C6L | Bcl-2 domain, IFN-β inhibitor | 11 |
| D7L | Com(10918..12900) | D7L | / | Host range; secreted IL-18 binding protein | 5 |
| E3L | Com(46022..46483) | F3L | E3L | Z-DNA binding domain, dsRNA-binding | 11 |
| F1L | Com(28603..29262) | C7L | F1L | Apoptosis inhibitor/caspase-9 inhibitor | 5,9,10 |
| Hemagglutinin | 158917..159858 | B2R | A56R | EEV envelope and cell membrane glycoprotein hemagglutinin | 5 |
| K1L | Com(23264..24118) | C1L | K1L | Host range; Ankyrin-like protein | 5 |
| K4L | Com(25823..27097) | C4L | K4L | Phospholipase D-like protein | 5,11,12 |
| N1R | 189199..189660 | N1R | C16L, B22R | Ubiquitin-protein ligase p28-like protein | 13 |
| N2L | Com(20531..21064) | P2L | N2L | Bcl-2 domain, IRF3 activation inhibitor, α-amanitin target | 11 |
| O1L | Com(55128..57125) | Q1L | O1L | Intracellular enveloped virion (IEV) morphogenesis protein | 11 |
| O1L_trctd | Com(21105..22433) | O1L | M1L | Ankyrin-like protein, apoptosis inhibitor | 5,11 |
| P1L | Com(20051..20404) | P1L | N1L | Virulence (Cytoplasmic) protein, anti-apoptotic Bcl-2-like protein | 5 |
| Rep2 | 190788..196450 | / | / | Long terminal repeat | this study |
| T4 | 168230..168895 | B10R | B9R | Kelch-like protein | 9 |
| v-slfn | 161596..163107 | B4R | B2R | Ankyrin repeat protein | 5,9,10,12 |
| B14R^**^ | 172403..173383 | B14R | B16R | IL-1β binding protein, Serine Protease Inhibitor-2 | 5,11,8 |
| D14L^**^ | Com(19060..19710) | D14L | C4L | Secreted complement binding protein | 5,11,8 |

**Notes:**

^*^ Com in front of the CDS column refers to this gene locates in the reverse complementary chain of genome.

^**^ The CDS starting and ending of this gene refer to Zaire-96-I-16 strain of monkeypox virus (NC_003310, belonging to the Congo Basin clade), while those of other genes or sequences refer to MPXV-M5312_HM12_Rivers isolate (NC_063383, belonging to the West African clade).

^a^ This gene is similar to that of the VACV-WR (Vaccinia Virus Western Reserve strain).

^b^ This gene is similar to that of the VACV-Cop (Vaccinia Virus Copenhagen strain).

**Additional references:**

1. Weaver, J. R. & Isaacs, S. N. Monkeypox virus and insights into its immunomodulatory proteins. *Immunol Rev*. **225**, 96-113 (2008).
2. Xu, Z. *et al*. Identification of 10 cowpox virus proteins that are necessary for induction of hemorrhagic lesions (red pocks) on chorioallantoic membranes. *J Virol*. **88**, 8615-8628 (2014).
3. Xu, Z., Zikos, D., Osterrieder, N. & Tischer, B. K. Generation of a complete single-gene knockout bacterial artificial chromosome library of cowpox virus and identification of its essential genes. *J Virol*. **88**, 490-502 (2014).
4. Afonso, P. P. *et al*. Biological characterization and next-generation genome sequencing of the unclassified Cotia virus SPAn232 (Poxviridae). *J Virol*. **86**, 5039-5054 (2012).
5. Senkevich, T. G., Yutin, N., Wolf, Y. I., Koonin, E. V. & Moss, B. Ancient Gene Capture and Recent Gene Loss Shape the Evolution of Orthopoxvirus-Host Interaction Genes. *mBio* **12**, e0149521 (2021).
6. Morikawa, S. *et al*. An attenuated LC16m8 smallpox vaccine: analysis of full-genome sequence and induction of immune protection. *J Virol*. **79**, 11873-11891 (2005).
7. Odom, M. R., Hendrickson, R. C. & Lefkowitz, E. J. Poxvirus protein evolution: family wide assessment of possible horizontal gene transfer events. *Virus Res*. **144**, 233-249 (2009).
