# Supplementary material for: Monkeypox virus 2022, gene heterogeneity and protein polymorphism
Source: Signal Transduct Target Ther. 2023 Jul 17;8:278. doi: 10.1038/s41392-023-01540-2 (PMC10352349; doi:10.1038/s41392-023-01540-2)
Supplement: Supplementary file 2 — Table S2 [file 41392_2023_1540_MOESM2_ESM.docx]

**Table S2. Unique clusters and their redundant genomes***

| >Cluster 0 | |
| --- | --- |
| 0 | 198450aa, >hMpxV/USA/VA-DHCPPCDC-002/2022\|EPI ISL 13096615\|2022-05...^#^ |
| >Cluster 1 | |
| 0 | 198450aa, >hMpxV/Spain/MD-HGUGM-6532064/2022\|EPI ISL 13106454\|2022-05-31...^#^ |
| >Cluster 2 | |
| 0 | 198450aa, >hMpxV/Germany/un-RKI-025/2022\|EPI ISL 13148263\|2022-05...^#^ |
| 1 | 198450aa, >hMpxV/Germany/un-RKI-019/2022\|EPI ISL 13148264\|2022-05...at 99.95% |
| 2 | 198450aa, >hMpxV/Germany/un-RKI-015/2022\|EPI ISL 13148265\|2022-05...at 99.87% |
| 3 | 198450aa, >hMpxV/Germany/un-RKI-023/2022\|EPI ISL 13148266\|2022-05...at 99.87% |
| 4 | 198450aa, >hMpxV/Germany/un-RKI-022/2022\|EPI ISL 13148267\|2022-05...at 99.95% |
| 5 | 198450aa, >hMpxV/Germany/un-RKI-021/2022\|EPI ISL 13148268\|2022-05...at 99.95% |
| 6 | 198450aa, >hMpxV/Germany/un-RKI-018/2022\|EPI ISL 13148269\|2022-05...at 96.56% |
| 7 | 198450aa, >hMpxV/Germany/un-RKI-024/2022\|EPI ISL 13148270\|2022-05...at 99.96% |
| 8 | 198450aa, >hMpxV/Germany/un-RKI-020/2022\|EPI ISL 13148271\|2022-05...at 99.93% |
| 9 | 198450aa, >hMpxV/Germany/un-RKI-012/2022\|EPI ISL 13148272\|2022-05...at 99.94% |
| 10 | 198450aa, >hMpxV/Germany/un-RKI-017/2022\|EPI ISL 13148273\|2022-05...at 99.88% |
| 11 | 198450aa, >hMpxV/Germany/un-RKI-016/2022\|EPI ISL 13148274\|2022-05...at 99.91% |
| 12 | 198450aa, >hMpxV/Germany/un-RKI-014/2022\|EPI ISL 13148275\|2022-05...at 98.71% |
| 13 | 198450aa, >hMpxV/Germany/un-RKI-011/2022\|EPI ISL 13148276\|2022-05...at 99.93% |
| 14 | 198450aa, >hMpxV/Germany/un-RKI-013/2022\|EPI ISL 13308117\|2022-06...at 98.68% |
| 15 | 198450aa, >hMpxV/Germany/un-RKI-026/2022\|EPI ISL 13308118\|2022-06...at 98.67% |
| 16 | 198450aa, >hMpxV/Germany/un-RKI-027/2022\|EPI ISL 13308119\|2022-06...at 98.67% |
| 17 | 198450aa, >hMpxV/Germany/un-RKI-028/2022\|EPI ISL 13308121\|2022-06...at 98.69% |
| 18 | 198450aa, >hMpxV/Germany/un-RKI-029/2022\|EPI ISL 13308122\|2022-06...at 98.68% |
| 19 | 198450aa, >hMpxV/Germany/un-RKI-030/2022\|EPI ISL 13308124\|2022-06...at 98.49% |
| 20 | 198450aa, >hMpxV/Germany/un-RKI-031/2022\|EPI ISL 13308125\|2022-06...at 98.67% |
| 21 | 198450aa, >hMpxV/Germany/un-RKI-032/2022\|EPI ISL 13308127\|2022-06...at 98.32% |
| 22 | 198450aa, >hMpxV/Germany/un-RKI-033/2022\|EPI ISL 13308129\|2022-06...at 98.65% |
| 23 | 198450aa, >hMpxV/Germany/un-RKI-035/2022\|EPI ISL 13308131\|2022-06...at 98.67% |
| 24 | 198450aa, >hMpxV/Germany/un-RKI-034/2022\|EPI ISL 13308133\|2022-06...at 98.68% |
| 25 | 198450aa, >hMpxV/Germany/un-RKI-036/2022\|EPI ISL 13308135\|2022-06...at 98.69% |
| 26 | 198450aa, >hMpxV/Germany/un-RKI-037/2022\|EPI ISL 13308137\|2022-06...at 98.69% |
| 27 | 198450aa, >hMpxV/Germany/un-RKI-038/2022\|EPI ISL 13308139\|2022-06...at 98.67% |
| 28 | 198450aa, >hMpxV/Germany/un-RKI-039/2022\|EPI ISL 13308140\|2022-06...at 98.67% |
| 29 | 198450aa, >hMpxV/Germany/un-RKI-040/2022\|EPI ISL 13308142\|2022-06...at 98.34% |
| 30 | 198450aa, >hMpxV/Germany/un-RKI-041/2022\|EPI ISL 13308144\|2022-06...at 98.63% |
| 31 | 198450aa, >hMpxV/Germany/un-RKI-042/2022\|EPI ISL 13308145\|2022-06...at 98.68% |
| 32 | 198450aa, >hMpxV/Germany/un-RKI-043/2022\|EPI ISL 13308146\|2022-06...at 98.48% |
| 33 | 198450aa, >hMpxV/Germany/un-RKI-044/2022\|EPI ISL 13308147\|2022-06...at 98.66% |
| 34 | 198450aa, >hMpxV/Germany/un-RKI-045/2022\|EPI ISL 13308148\|2022-06...at 98.43% |
| 35 | 198450aa, >hMpxV/Germany/un-RKI-046/2022\|EPI ISL 13308150\|2022-06...at 98.68% |
| 36 | 198450aa, >hMpxV/Germany/un-RKI-047/2022\|EPI ISL 13308151\|2022-06...at 98.63% |
| 37 | 198450aa, >hMpxV/Germany/un-RKI-048/2022\|EPI ISL 13308153\|2022-06...at 98.68% |
| 38 | 198450aa, >hMpxV/Germany/un-RKI-050/2022\|EPI ISL 13308157\|2022-06...at 98.68% |
| 39 | 198450aa, >hMpxV/Germany/un-RKI-067/2022\|EPI ISL 13411153\|2022-06...at 98.68% |
| 40 | 198450aa, >hMpxV/Germany/un-RKI-065/2022\|EPI ISL 13411154\|2022-06...at 98.64% |
| 41 | 198450aa, >hMpxV/Germany/un-RKI-064/2022\|EPI ISL 13411155\|2022-06...at 98.68% |
| 42 | 198450aa, >hMpxV/Germany/un-RKI-063/2022\|EPI ISL 13411156\|2022-06...at 98.69% |
| 43 | 198450aa, >hMpxV/Germany/un-RKI-062/2022\|EPI ISL 13411157\|2022-06...at 98.67% |
| 44 | 198450aa, >hMpxV/Germany/un-RKI-061/2022\|EPI ISL 13411158\|2022-06...at 98.65% |
| 45 | 198450aa, >hMpxV/Germany/un-RKI-060/2022\|EPI ISL 13411159\|2022-06...at 98.56% |
| 46 | 198450aa, >hMpxV/Germany/un-RKI-059/2022\|EPI ISL 13411160\|2022-06...at 98.69% |
| 47 | 198450aa, >hMpxV/Germany/un-RKI-058/2022\|EPI ISL 13411161\|2022-06...at 98.66% |
| 48 | 198450aa, >hMpxV/Germany/un-RKI-057/2022\|EPI ISL 13411162\|2022-06...at 98.65% |
| 49 | 198450aa, >hMpxV/Germany/un-RKI-056/2022\|EPI ISL 13411163\|2022-06...at 98.66% |
| 50 | 198450aa, >hMpxV/Germany/un-RKI-055/2022\|EPI ISL 13411164\|2022-06...at 98.65% |
| 51 | 198450aa, >hMpxV/Germany/un-RKI-054/2022\|EPI ISL 13411165\|2022-06...at 98.68% |
| 52 | 198450aa, >hMpxV/Germany/un-RKI-053/2022\|EPI ISL 13411166\|2022-06...at 98.42% |
| 53 | 198450aa, >hMpxV/Germany/un-RKI-052/2022\|EPI ISL 13411167\|2022-06...at 98.67% |
| 54 | 198450aa, >hMpxV/Germany/un-RKI-051/2022\|EPI ISL 13411168\|2022-06...at 98.28% |
| 55 | 198450aa, >hMpxV/Germany/un-RKI-068/2022\|EPI ISL 13483155\|2022-06...at 98.67% |
| 56 | 198450aa, >hMpxV/Germany/un-RKI-069/2022\|EPI ISL 13483157\|2022-06...at 98.66% |
| 57 | 198450aa, >hMpxV/Germany/un-RKI-073/2022\|EPI ISL 13483159\|2022-06...at 98.67% |
| 58 | 198450aa, >hMpxV/Germany/un-RKI-074/2022\|EPI ISL 13483161\|2022-06...at 98.65% |
| 59 | 198450aa, >hMpxV/Germany/un-RKI-076/2022\|EPI ISL 13483162\|2022-06...at 98.65% |
| 60 | 198450aa, >hMpxV/Germany/un-RKI-087/2022\|EPI ISL 13483163\|2022-06...at 98.66% |
| 61 | 198450aa, >hMpxV/Germany/un-RKI-088/2022\|EPI ISL 13483164\|2022-06...at 98.69% |
| 62 | 198450aa, >hMpxV/Germany/un-RKI-070/2022\|EPI ISL 13483165\|2022-06...at 98.65% |
| 63 | 198450aa, >hMpxV/Germany/un-RKI-071/2022\|EPI ISL 13483167\|2022-06...at 98.55% |
| 64 | 198450aa, >hMpxV/Germany/un-RKI-072/2022\|EPI ISL 13483168\|2022-06...at 98.39% |
| 65 | 198450aa, >hMpxV/Germany/un-RKI-075/2022\|EPI ISL 13483170\|2022-06...at 98.45% |
| 66 | 198450aa, >hMpxV/Germany/un-RKI-078/2022\|EPI ISL 13483171\|2022-06...at 98.66% |
| 67 | 198450aa, >hMpxV/Germany/un-RKI-079/2022\|EPI ISL 13483173\|2022-06...at 98.64% |
| 68 | 198450aa, >hMpxV/Germany/un-RKI-089/2022\|EPI ISL 13483187\|2022-06...at 98.66% |
| 69 | 198450aa, >hMpxV/Germany/un-RKI-090/2022\|EPI ISL 13483188\|2022-06...at 98.68% |
| 70 | 198450aa, >hMpxV/Germany/un-RKI-099/2022\|EPI ISL 13483200\|2022-06...at 98.31% |
| 71 | 198450aa, >hMpxV/Germany/un-RKI-100/2022\|EPI ISL 13483201\|2022-06...at 98.62% |
| 72 | 198450aa, >hMpxV/Germany/un-RKI-077/2022\|EPI ISL 13483203\|2022-06...at 98.65% |
| 73 | 198450aa, >hMpxV/Germany/un-RKI-097/2022\|EPI ISL 13483205\|2022-06...at 98.60% |
| 74 | 198450aa, >hMpxV/Germany/un-RKI-092/2022\|EPI ISL 13483206\|2022-06...at 98.65% |
| 75 | 198450aa, >hMpxV/Germany/un-RKI-101/2022\|EPI ISL 13483208\|2022-06...at 98.53% |
| 76 | 198450aa, >hMpxV/Peru/LIM-INS-001/2022\|EPI ISL 13530881\|2022-06-25...at 95.26% |
| 77 | 198450aa, >hMpxV/Germany/un-RKI-102/2022\|EPI ISL 13734237\|2022-07...at 98.42% |
| 78 | 198450aa, >hMpxV/Germany/un-RKI-103/2022\|EPI ISL 13734238\|2022-07...at 98.64% |
| 79 | 198450aa, >hMpxV/Germany/un-RKI-105/2022\|EPI ISL 13734240\|2022-07...at 98.63% |
| 80 | 198450aa, >hMpxV/Germany/un-RKI-106/2022\|EPI ISL 13734241\|2022-07...at 98.64% |
| 81 | 198450aa, >hMpxV/Germany/un-RKI-107/2022\|EPI ISL 13734242\|2022-07...at 98.61% |
| 82 | 198450aa, >hMpxV/Germany/un-RKI-108/2022\|EPI ISL 13734243\|2022-07...at 98.65% |
| 83 | 198450aa, >hMpxV/Germany/un-RKI-109/2022\|EPI ISL 13734244\|2022-07...at 98.64% |
| 84 | 198450aa, >hMpxV/Germany/un-RKI-110/2022\|EPI ISL 13734245\|2022-07...at 98.64% |
| 85 | 198450aa, >hMpxV/Germany/un-RKI-111/2022\|EPI ISL 13734246\|2022-07...at 98.63% |
| 86 | 198450aa, >hMpxV/Germany/un-RKI-112/2022\|EPI ISL 13734247\|2022-07...at 98.64% |
| 87 | 198450aa, >hMpxV/Germany/un-RKI-113/2022\|EPI ISL 13734248\|2022-07...at 98.64% |
| 88 | 198450aa, >hMpxV/Germany/un-RKI-114/2022\|EPI ISL 13734249\|2022-07...at 98.64% |
| 89 | 198450aa, >hMpxV/Germany/un-RKI-115/2022\|EPI ISL 13734250\|2022-07...at 98.44% |
| 90 | 198450aa, >hMpxV/Germany/un-RKI-119/2022\|EPI ISL 13734251\|2022-07...at 98.64% |
| 91 | 198450aa, >hMpxV/Germany/un-RKI-120/2022\|EPI ISL 13734252\|2022-07...at 98.63% |
| 92 | 198450aa, >hMpxV/Germany/un-RKI-121/2022\|EPI ISL 13734253\|2022-07...at 98.64% |
| 93 | 198450aa, >hMpxV/Germany/un-RKI-122/2022\|EPI ISL 13734254\|2022-07...at 98.25% |
| 94 | 198450aa, >hMpxV/Germany/un-RKI-123/2022\|EPI ISL 13734255\|2022-07...at 98.67% |
| 95 | 198450aa, >hMpxV/Germany/un-RKI-124/2022\|EPI ISL 13734256\|2022-07...at 98.64% |
| 96 | 198450aa, >hMpxV/Germany/un-RKI-125/2022\|EPI ISL 13734257\|2022-07...at 98.64% |
| 97 | 198450aa, >hMpxV/Germany/un-RKI-126/2022\|EPI ISL 13734258\|2022-07...at 98.62% |
| 98 | 198450aa, >hMpxV/Germany/un-RKI-127/2022\|EPI ISL 13734259\|2022-07...at 98.62% |
| 99 | 198450aa, >hMpxV/Germany/BE-ChVir28305/2022\|EPI ISL 13889908\|2022-06-05...at 95.06% |
| 100 | 198450aa, >hMpxV/Germany/BE-ChVir28566/2022\|EPI ISL 13890048\|2022-06-16...at 95.25% |
| 101 | 198450aa, >hMpxV/Germany/un-RKI166/2022\|EPI ISL 13908350\|2022-07...at 98.58% |
| 102 | 198450aa, >hMpxV/Germany/un-RKI167/2022\|EPI ISL 13908351\|2022-07...at 98.64% |
| 103 | 198450aa, >hMpxV/Germany/un-RKI168/2022\|EPI ISL 13908352\|2022-07...at 98.65% |
| 104 | 198450aa, >hMpxV/Germany/un-RKI170/2022\|EPI ISL 13908353\|2022-07...at 98.64% |
| 105 | 198450aa, >hMpxV/Germany/un-RKI171/2022\|EPI ISL 13908354\|2022-07...at 98.64% |
| 106 | 198450aa, >hMpxV/Germany/un-RKI172/2022\|EPI ISL 13908355\|2022-07...at 98.64% |
| 107 | 198450aa, >hMpxV/Germany/un-RKI173/2022\|EPI ISL 13908356\|2022-07...at 98.65% |
| 108 | 198450aa, >hMpxV/Germany/un-RKI174/2022\|EPI ISL 13908357\|2022-07...at 98.55% |
| 109 | 198450aa, >hMpxV/Germany/un-RKI175/2022\|EPI ISL 13908358\|2022-07...at 98.66% |
| 110 | 198450aa, >hMpxV/Germany/un-RKI176/2022\|EPI ISL 13908359\|2022-07...at 98.67% |
| 111 | 198450aa, >hMpxV/Slovakia/CUSP-33057/2022\|EPI ISL 13955501\|2022-07-04...at 95.22% |
| 112 | 198450aa, >hMpxV/Spain/un-ISCIII-353 R/2022\|EPI ISL 14181948\|2022...at 95.02% |
| 113 | 198450aa, >hMpxV/Spain/un-ISCIII-351 R/2022\|EPI ISL 14181949\|2022...at 95.03% |
| 114 | 198450aa, >hMpxV/Spain/un-ISCIII-345 R/2022\|EPI ISL 14181951\|2022...at 95.05% |
| 115 | 198450aa, >hMpxV/Spain/un-ISCIII-350 R/2022\|EPI ISL 14181952\|2022...at 95.04% |
| 116 | 198450aa, >hMpxV/Spain/un-ISCIII-352 R/2022\|EPI ISL 14181953\|2022...at 95.04% |
| 117 | 198450aa, >hMpxV/Spain/un-ISCIII-349 R/2022\|EPI ISL 14181954\|2022...at 95.02% |
| 118 | 198450aa, >hMpxV/Spain/un-ISCIII-399/2022\|EPI ISL 14181955\|2022...at 95.04% |
| 119 | 198450aa, >hMpxV/Spain/un-ISCIII-453/2022\|EPI ISL 14181956\|2022...at 95.03% |
| 120 | 198450aa, >hMpxV/Spain/un-ISCIII-395/2022\|EPI ISL 14181957\|2022...at 95.00% |
| 121 | 198450aa, >hMpxV/Spain/un-ISCIII-403/2022\|EPI ISL 14181958\|2022...at 95.05% |
| 122 | 198450aa, >hMpxV/Spain/un-ISCIII-457/2022\|EPI ISL 14181959\|2022...at 95.04% |
| 123 | 198450aa, >hMpxV/Germany/BY-IMB-25241/2022\|EPI ISL 13052263\|2022-05-19...at 95.02% |
| 124 | 198450aa, >hMpxV/England/UKHSA-1/2022\|EPI ISL 13052278\|2022-05...at 95.05% |
| 125 | 198450aa, >hMpxV/England/UKHSA-2/2022\|EPI ISL 13052279\|2022-05...at 95.10% |
| 126 | 198450aa, >hMpxV/England/UKHSA-4/2022\|EPI ISL 13052281\|2022-05...at 95.11% |
| 127 | 198450aa, >hMpxV/France/un-VGEMI-HCL0001/2022\|EPI ISL 13052287\|2022-05-22...at 95.31% |
| 128 | 198450aa, >hMpxV/USA/UT-UPHL-82200022/2022\|EPI ISL 13052288\|2022-05-20...at 95.00% |
| 129 | 198450aa, >hMpxV/Italy/un-INMI-Pt2/2022\|EPI ISL 13251120\|2022-05-19...at 95.01% |
| 130 | 198450aa, >hMpxV/Italy/un-INMI-Pt3/2022\|EPI ISL 13331712\|2022-05-19...at 95.02% |
| 131 | 198450aa, >hMpxV/Italy/un-INMI-Pt4/2022\|EPI ISL 13331713\|2022-05-21...at 95.06% |
| 132 | 198450aa, >hMpxV/Finland/DVFM-37/2022\|EPI ISL 13331714\|2022-05...at 97.21% |
| 133 | 198450aa, >hMpxV/Finland/DVFM-42/2022\|EPI ISL 13331715\|2022...at 97.21% |
| 134 | 198450aa, >hMpxV/Germany/un-RKI-081/2022\|EPI ISL 13483177\|2022-06...at 98.65% |
| 135 | 198450aa, >hMpxV/Germany/un-RKI-082/2022\|EPI ISL 13483178\|2022-06...at 98.38% |
| 136 | 198450aa, >hMpxV/Germany/un-RKI-083/2022\|EPI ISL 13483180\|2022-06...at 98.66% |
| 137 | 198450aa, >hMpxV/Germany/un-RKI-084/2022\|EPI ISL 13483182\|2022-06...at 98.60% |
| 138 | 198450aa, >hMpxV/Germany/un-RKI-085/2022\|EPI ISL 13483183\|2022-06...at 98.61% |
| 139 | 198450aa, >hMpxV/Germany/un-RKI-086/2022\|EPI ISL 13483185\|2022-06...at 98.62% |
| 140 | 198450aa, >hMpxV/Germany/un-RKI-091/2022\|EPI ISL 13483190\|2022-06...at 98.39% |
| 141 | 198450aa, >hMpxV/Germany/un-RKI-093/2022\|EPI ISL 13483191\|2022-06...at 98.66% |
| 142 | 198450aa, >hMpxV/Germany/un-RKI-094/2022\|EPI ISL 13483193\|2022-06...at 98.53% |
| 143 | 198450aa, >hMpxV/Germany/un-RKI-095/2022\|EPI ISL 13483195\|2022-06...at 98.59% |
| 144 | 198450aa, >hMpxV/Germany/un-RKI-096/2022\|EPI ISL 13483196\|2022-06...at 98.66% |
| 145 | 198450aa, >hMpxV/Brazil/RJ-FIOCRUZ-14992/2022\|EPI ISL 13484458\|2022-06-16...at 95.18% |
| 146 | 198450aa, >hMpxV/Germany/un-UMR-124759/2022\|EPI ISL 13586184\|2022-06-23...at 95.27% |
| 147 | 198450aa, >hMpxV/Germany/un-RKI-164/2022\|EPI ISL 13744931\|2022-07...at 98.64% |
| 148 | 198450aa, >hMpxV/Netherlands/un-EMC-NL008/2022\|EPI ISL 13822667\|2022-06-27...at 95.01% |
| 149 | 198450aa, >hMpxV/Peru/LIM-INS-006/2022\|EPI ISL 13833195\|2022-07-01...at 95.27% |
| 150 | 198450aa, >hMpxV/Peru/LIM-INS-007/2022\|EPI ISL 13833196\|2022-07-01...at 95.27% |
| 151 | 198450aa, >hMpxV/Peru/LIM-INS-008/2022\|EPI ISL 13833197\|2022-07-01...at 95.27% |
| 152 | 198450aa, >hMpxV/Germany/BE-ChVir28154/2022\|EPI ISL 13889436\|2022-05-28...at 95.06% |
| 153 | 198450aa, >hMpxV/Germany/BE-ChVir28679/2022\|EPI ISL 13889438\|2022-06-22...at 95.06% |
| 154 | 198450aa, >hMpxV/Germany/BE-ChVir28689/2022\|EPI ISL 13889439\|2022-06-24...at 95.06% |
| 155 | 198450aa, >hMpxV/Germany/BE-ChVir28707/2022\|EPI ISL 13889440\|2022-06-25...at 95.06% |
| 156 | 198450aa, >hMpxV/Germany/BE-ChVir28474/2022\|EPI ISL 13889441\|2022-06-12...at 95.32% |
| 157 | 198450aa, >hMpxV/Germany/BE-ChVir28675/2022\|EPI ISL 13889443\|2022-06-23...at 95.07% |
| 158 | 198450aa, >hMpxV/Germany/BE-ChVir28705/2022\|EPI ISL 13889444\|2022-06-26...at 95.06% |
| 159 | 198450aa, >hMpxV/Germany/BE-ChVir28473/2022\|EPI ISL 13889445\|2022-06-12...at 95.32% |
| 160 | 198450aa, >hMpxV/Germany/BE-ChVir28149/2022\|EPI ISL 13889446\|2022-05-28...at 95.32% |
| 161 | 198450aa, >hMpxV/Germany/BE-ChVir28220/2022\|EPI ISL 13889447\|2022-06-01...at 95.05% |
| 162 | 198450aa, >hMpxV/Germany/BE-ChVir28581/2022\|EPI ISL 13889448\|2022-06-17...at 95.07% |
| 163 | 198450aa, >hMpxV/Germany/BE-ChVir28684/2022\|EPI ISL 13889449\|2022-06-23...at 95.06% |
| 164 | 198450aa, >hMpxV/Germany/BE-ChVir28539/2022\|EPI ISL 13889450\|2022-06-14...at 95.03% |
| 165 | 198450aa, >hMpxV/Germany/BE-ChVir28152/2022\|EPI ISL 13889660\|2022-05-29...at 95.06% |
| 166 | 198450aa, >hMpxV/Germany/BE-ChVir28451/2022\|EPI ISL 13889796\|2022-06-09...at 95.28% |
| 167 | 198450aa, >hMpxV/Germany/BE-ChVir28292/2022\|EPI ISL 13890135\|2022-05-30...at 95.05% |
| 168 | 198450aa, >hMpxV/Germany/BE-ChVir28138/2022\|EPI ISL 13890273\|2022-05-20...at 95.27% |
| 169 | 198450aa, >hMpxV/Germany/BE-ChVir28388/2022\|EPI ISL 13890338\|2022-06-08...at 95.01% |
| 170 | 198450aa, >hMpxV/Germany/BE-ChVir28443/2022\|EPI ISL 13890464\|2022-06-09...at 95.30% |
| 171 | 198450aa, >hMpxV/Germany/BE-ChVir28633/2022\|EPI ISL 13890465\|2022-06-21...at 95.07% |
| 172 | 198450aa, >hMpxV/Germany/BE-ChVir28148/2022\|EPI ISL 13890467\|2022-05-27...at 95.30% |
| 173 | 198450aa, >hMpxV/Germany/BE-ChVir28599/2022\|EPI ISL 13890468\|2022-06-16...at 95.06% |
| 174 | 198450aa, >hMpxV/Germany/BE-ChVir28682/2022\|EPI ISL 13890469\|2022-06-23...at 95.06% |
| 175 | 198450aa, >hMpxV/Germany/BE-ChVir28446/2022\|EPI ISL 13890471\|2022-06-10...at 95.32% |
| 176 | 198450aa, >hMpxV/Germany/BE-ChVir28685/2022\|EPI ISL 13890473\|2022-06-23...at 95.06% |
| 177 | 198450aa, >hMpxV/Germany/BE-ChVir28209/2022\|EPI ISL 13890474\|2022-05-30...at 95.30% |
| 178 | 198450aa, >hMpxV/Germany/BE-ChVir28274/2022\|EPI ISL 13890475\|2022-05-31...at 95.29% |
| 179 | 198450aa, >hMpxV/Germany/BE-ChVir28396/2022\|EPI ISL 13890476\|2022-06-09...at 95.32% |
| 180 | 198450aa, >hMpxV/Germany/BE-ChVir28718/2022\|EPI ISL 13890478\|2022-06-28...at 95.04% |
| 181 | 198450aa, >hMpxV/Germany/BE-ChVir28146/2022\|EPI ISL 13890481\|2022-05-27...at 95.33% |
| 182 | 198450aa, >hMpxV/Germany/un-RKI169/2022\|EPI ISL 13908346\|2022-07...at 98.64% |
| 183 | 198450aa, >hMpxV/Germany/un-RKI180/2022\|EPI ISL 13908347\|2022-07...at 98.59% |
| 184 | 198450aa, >hMpxV/Germany/un-RKI181/2022\|EPI ISL 13908348\|2022-07...at 98.27% |
| 185 | 198450aa, >hMpxV/Germany/un-RKI183/2022\|EPI ISL 13908349\|2022-07...at 98.64% |
| 186 | 198450aa, >hMpxV/Thailand/NIC-74/2022\|EPI ISL 13983888\|2022-07-17...at 98.61% |
| 187 | 198450aa, >hMpxV/USA/NY-URMC-2207A101/2022\|EPI ISL 14003930\|2022-07-11...at 95.26% |
| 188 | 198450aa, >hMpxV/Peru/LIM-INS-013/2022\|EPI ISL 14207728\|2022-07-08...at 95.27% |
| 189 | 198450aa, >hMpxV/Slovakia/PHA-CU-0802 34042/2022\|EPI ISL 14211645\|2022-07-20...at 95.01% |
| 190 | 198450aa, >hMpxV/USA/NY-URMC-2207A102/2022\|EPI ISL 14251112\|2022-07-14...at 95.26% |
| 191 | 198450aa, >hMpxV/USA/TX-DHCPPCDC-007/2021\|EPI ISL 13100621\|2021-07...at 98.61% |
| 192 | 198450aa, >hMpxV/Germany/un-RKI-04/2022\|EPI ISL 13117293\|2022-05...at 98.66% |
| 193 | 198450aa, >hMpxV/Germany/un-RKI-09/2022\|EPI ISL 13117294\|2022-05...at 99.85% |
| 194 | 198450aa, >hMpxV/Germany/un-RKI-010/2022\|EPI ISL 13117295\|2022-05...at 99.90% |
| 195 | 198450aa, >hMpxV/Germany/un-RKI-08/2022\|EPI ISL 13117296\|2022-05...at 98.70% |
| 196 | 198450aa, >hMpxV/Germany/un-RKI-07/2022\|EPI ISL 13117297\|2022-05...at 98.65% |
| 197 | 198450aa, >hMpxV/Germany/un-RKI-06/2022\|EPI ISL 13117298\|2022-05...at 98.69% |
| 198 | 198450aa, >hMpxV/Italy/APU-IZSPB-0001/2022\|EPI ISL 13362760\|2022-06-08...at 95.93% |
| 199 | 198450aa, >hMpxV/Germany/un-UMR-124757/2022\|EPI ISL 13584854\|2022-06-23...at 95.29% |
| 200 | 198450aa, >hMpxV/Germany/un-RKI-128/2022\|EPI ISL 13734260\|2022-07...at 98.64% |
| 201 | 198450aa, >hMpxV/Germany/un-RKI-129/2022\|EPI ISL 13734261\|2022-07...at 98.64% |
| 202 | 198450aa, >hMpxV/Germany/un-RKI-130/2022\|EPI ISL 13734262\|2022-07...at 98.64% |
| 203 | 198450aa, >hMpxV/Germany/un-RKI-131/2022\|EPI ISL 13734263\|2022-07...at 98.64% |
| 204 | 198450aa, >hMpxV/Germany/un-RKI-132/2022\|EPI ISL 13734264\|2022-07...at 98.51% |
| 205 | 198450aa, >hMpxV/Germany/un-RKI-133/2022\|EPI ISL 13734265\|2022-07...at 98.64% |
| 206 | 198450aa, >hMpxV/Germany/un-RKI-134/2022\|EPI ISL 13734266\|2022-07...at 98.64% |
| 207 | 198450aa, >hMpxV/Germany/un-RKI-135/2022\|EPI ISL 13734267\|2022-07...at 98.64% |
| 208 | 198450aa, >hMpxV/Germany/un-RKI-136/2022\|EPI ISL 13734268\|2022-07...at 98.64% |
| 209 | 198450aa, >hMpxV/Finland/DVFM-96/2022\|EPI ISL 13744902\|2022-06-16...at 97.21% |
| 210 | 198450aa, >hMpxV/Germany/un-RKI-140/2022\|EPI ISL 13744903\|2022-07...at 98.64% |
| 211 | 198450aa, >hMpxV/Germany/un-RKI-145/2022\|EPI ISL 13744904\|2022-07...at 98.63% |
| 212 | 198450aa, >hMpxV/Germany/un-RKI-160/2022\|EPI ISL 13744905\|2022-07...at 98.64% |
| 213 | 198450aa, >hMpxV/Germany/un-RKI-139/2022\|EPI ISL 13744906\|2022-07...at 98.64% |
| 214 | 198450aa, >hMpxV/Germany/un-RKI-165/2022\|EPI ISL 13744907\|2022-07...at 98.64% |
| 215 | 198450aa, >hMpxV/Germany/un-RKI-137/2022\|EPI ISL 13744908\|2022-07...at 98.63% |
| 216 | 198450aa, >hMpxV/Germany/un-RKI-138/2022\|EPI ISL 13744909\|2022-07...at 98.64% |
| 217 | 198450aa, >hMpxV/Germany/un-RKI-141/2022\|EPI ISL 13744910\|2022-07...at 98.64% |
| 218 | 198450aa, >hMpxV/Germany/un-RKI-142/2022\|EPI ISL 13744911\|2022-07...at 98.64% |
| 219 | 198450aa, >hMpxV/Germany/un-RKI-143/2022\|EPI ISL 13744912\|2022-07...at 98.59% |
| 220 | 198450aa, >hMpxV/Germany/un-RKI-144/2022\|EPI ISL 13744913\|2022-07...at 98.64% |
| 221 | 198450aa, >hMpxV/Germany/un-RKI-146/2022\|EPI ISL 13744914\|2022-07...at 98.64% |
| 222 | 198450aa, >hMpxV/Germany/un-RKI-147/2022\|EPI ISL 13744915\|2022-07...at 98.64% |
| 223 | 198450aa, >hMpxV/Germany/un-RKI-148/2022\|EPI ISL 13744916\|2022-07...at 98.64% |
| 224 | 198450aa, >hMpxV/Germany/un-RKI-149/2022\|EPI ISL 13744917\|2022-07...at 98.64% |
| 225 | 198450aa, >hMpxV/Germany/un-RKI-150/2022\|EPI ISL 13744918\|2022-07...at 98.64% |
| 226 | 198450aa, >hMpxV/Germany/un-RKI-151/2022\|EPI ISL 13744919\|2022-07...at 98.64% |
| 227 | 198450aa, >hMpxV/Germany/un-RKI-152/2022\|EPI ISL 13744920\|2022-07...at 98.60% |
| 228 | 198450aa, >hMpxV/Germany/un-RKI-153/2022\|EPI ISL 13744921\|2022-07...at 98.64% |
| 229 | 198450aa, >hMpxV/Germany/un-RKI-154/2022\|EPI ISL 13744922\|2022-07...at 98.64% |
| 230 | 198450aa, >hMpxV/Germany/un-RKI-155/2022\|EPI ISL 13744923\|2022-07...at 98.64% |
| 231 | 198450aa, >hMpxV/Germany/un-RKI-156/2022\|EPI ISL 13744924\|2022-07...at 98.64% |
| 232 | 198450aa, >hMpxV/Germany/un-RKI-157/2022\|EPI ISL 13744925\|2022-07...at 98.64% |
| 233 | 198450aa, >hMpxV/Germany/un-RKI-158/2022\|EPI ISL 13744926\|2022-07...at 98.63% |
| 234 | 198450aa, >hMpxV/Germany/un-RKI-159/2022\|EPI ISL 13744927\|2022-07...at 98.64% |
| 235 | 198450aa, >hMpxV/Germany/un-RKI-161/2022\|EPI ISL 13744928\|2022-07...at 98.64% |
| 236 | 198450aa, >hMpxV/Germany/un-RKI-162/2022\|EPI ISL 13744929\|2022-07...at 98.64% |
| 237 | 198450aa, >hMpxV/Germany/un-RKI-163/2022\|EPI ISL 13744930\|2022-07...at 98.64% |
| 238 | 198450aa, >hMpxV/Netherlands/un-EMC-NL009/2022\|EPI ISL 13822668\|2022-06-26...at 95.00% |
| 239 | 198450aa, >hMpxV/Germany/BE-ChVir28423/2022\|EPI ISL 13889515\|2022-06-09...at 95.04% |
| 240 | 198450aa, >hMpxV/Germany/BE-ChVir28604/2022\|EPI ISL 13889590\|2022-06-17...at 95.29% |
| 241 | 198450aa, >hMpxV/Germany/BE-ChVir28456/2022\|EPI ISL 13889977\|2022-06-10...at 95.06% |
| 242 | 198450aa, >hMpxV/Germany/BE-ChVir28703/2022\|EPI ISL 13890408\|2022-06-24...at 95.13% |
| 243 | 198450aa, >hMpxV/Germany/un-RKI177/2022\|EPI ISL 13908360\|2022-07...at 98.64% |
| 244 | 198450aa, >hMpxV/Germany/un-RKI178/2022\|EPI ISL 13908361\|2022-07...at 98.66% |
| 245 | 198450aa, >hMpxV/Germany/un-RKI179/2022\|EPI ISL 13908362\|2022-07...at 98.63% |
| 246 | 198450aa, >hMpxV/Germany/un-RKI182/2022\|EPI ISL 13908363\|2022-07...at 98.52% |
| 247 | 198450aa, >hMpxV/Germany/un-RKI184/2022\|EPI ISL 13908364\|2022-07...at 98.64% |
| 248 | 198450aa, >hMpxV/Germany/un-RKI185/2022\|EPI ISL 13908365\|2022-07...at 98.64% |
| 249 | 198450aa, >hMpxV/Thailand/CU-220016-FTV/2022\|EPI ISL 14011193\|2022-07-17...at 95.21% |
| 250 | 198450aa, >hMpxV/Thailand/NIC-251/2022\|EPI ISL 14153982\|2022-07-27...at 95.00% |
| 251 | 198450aa, >hMpxV/Peru/LIM-INS-010/2022\|EPI ISL 14207725\|2022-07-02...at 95.27% |
| >Cluster 3 | |
| 0 | 198450aa, >hMpxV/Switzerland/ZH-UZH-IMV-3ba6449f/2022\|EPI ISL 13251157\|2022-06-01...^#^ |
| 1 | 198450aa, >hMpxV/Brazil/RS-IAL-05/2022\|EPI ISL 13343697\|2022-06-07...at 99.86% |
| 2 | 198450aa, >hMpxV/Scotland/CVR-1d/2022\|EPI ISL 13409180\|2022-05-15...at 95.33% |
| 3 | 198450aa, >hMpxV/Scotland/CVR-1e/2022\|EPI ISL 13409181\|2022-05-15...at 95.33% |
| 4 | 198450aa, >hMpxV/Brazil/SP-IAL-08/2022\|EPI ISL 13436792\|2022-06-13...at 99.86% |
| 5 | 198450aa, >hMpxV/Brazil/SP-IAL-10/2022\|EPI ISL 13459346\|2022-06-21...at 99.86% |
| 6 | 198450aa, >hMpxV/Brazil/SP-IAL-11/2022\|EPI ISL 13459347\|2022-06-22...at 99.86% |
| 7 | 198450aa, >hMpxV/Brazil/SP-IAL-12/2022\|EPI ISL 13459482\|2022-06-22...at 99.86% |
| 8 | 198450aa, >hMpxV/Brazil/SP-IAL-13/2022\|EPI ISL 13459483\|2022-06-23...at 99.85% |
| 9 | 198450aa, >hMpxV/Peru/LIM-INS-003/2022\|EPI ISL 13651349\|2022-06-27...at 95.37% |
| 10 | 198450aa, >hMpxV/Peru/LIM-INS-004/2022\|EPI ISL 13651350\|2022-06-29...at 95.37% |
| 11 | 198450aa, >hMpxV/Taiwan/TCDC-110-364682/2022\|EPI ISL 13908328\|2022-07...at 99.88% |
| 12 | 198450aa, >hMpxV/Canada/un-NML-3518/2022\|EPI ISL 13908332\|2022-06-07...at 98.44% |
| 13 | 198450aa, >hMpxV/Canada/un-NML-3519/2022\|EPI ISL 13908333\|2022-06-06...at 98.43% |
| 14 | 198450aa, >hMpxV/Canada/un-NML-3542/2022\|EPI ISL 13908334\|2022-06-07...at 98.44% |
| 15 | 198450aa, >hMpxV/Canada/un-NML-3549/2022\|EPI ISL 13908335\|2022-06-07...at 98.28% |
| 16 | 198450aa, >hMpxV/Canada/un-NML-3582/2022\|EPI ISL 13908336\|2022-06-08...at 98.43% |
| 17 | 198450aa, >hMpxV/Canada/un-NML-3595/2022\|EPI ISL 13908337\|2022-06-08...at 98.42% |
| 18 | 198450aa, >hMpxV/Canada/un-NML-3607/2022\|EPI ISL 13908338\|2022-06-09...at 98.44% |
| 19 | 198450aa, >hMpxV/Canada/un-NML-3611/2022\|EPI ISL 13908339\|2022-06-09...at 98.44% |
| 20 | 198450aa, >hMpxV/Brazil/SP-IAL-21/2022\|EPI ISL 14021725\|2022-07-13...at 99.85% |
| 21 | 198450aa, >hMpxV/Brazil/SP-IAL-23/2022\|EPI ISL 14070852\|2022-07-19...at 99.92% |
| 22 | 198450aa, >hMpxV/Brazil/SP-IAL-24/2022\|EPI ISL 14070854\|2022-07-19...at 99.90% |
| 23 | 198450aa, >hMpxV/Brazil/SP-IAL-25/2022\|EPI ISL 14070855\|2022-07-19...at 99.92% |
| 24 | 198450aa, >hMpxV/Austria/MUW-1534823/2022\|EPI ISL 14167248\|2022-07-15...at 99.93% |
| 25 | 198450aa, >hMpxV/Austria/MUW-1531848/2022\|EPI ISL 14167573\|2022-07-05...at 99.93% |
| 26 | 198450aa, >hMpxV/Austria/MUW-1532243/2022\|EPI ISL 14167574\|2022-07-06...at 99.93% |
| 27 | 198450aa, >hMpxV/Austria/MUW-1536480/2022\|EPI ISL 14167575\|2022-07-22...at 99.93% |
| 28 | 198450aa, >hMpxV/USA/WA-CDC-PRB-001/2022\|EPI ISL 14244559\|2022-05...at 97.47% |
| 29 | 198450aa, >hMpxV/USA/MA-CDC-001/2022\|EPI ISL 13052289\|2022-05...at 99.93% |
| 30 | 198450aa, >hMpxV/Australia/VIC-VIDRL01/2022\|EPI ISL 13052292\|2022-05...at 95.16% |
| 31 | 198450aa, >hMpxV/Portugal/INSA-PT0023/2022\|EPI ISL 13056892\|2022-05-19...at 98.27% |
| 32 | 198450aa, >hMpxV/Portugal/INSA-PT0028/2022\|EPI ISL 13056893\|2022-05-18...at 98.41% |
| 33 | 198450aa, >hMpxV/Portugal/INSA-PT0025/2022\|EPI ISL 13056896\|2022-05-19...at 98.30% |
| 34 | 198450aa, >hMpxV/Portugal/INSA-PT0020/2022\|EPI ISL 13056897\|2022-05-19...at 95.06% |
| 35 | 198450aa, >hMpxV/Israel/IIBR-ISR001/2022\|EPI ISL 13056910\|2022-05-20...at 95.11% |
| 36 | 198450aa, >hMpxV/Canada/AB-APL-01/2022\|EPI ISL 13194516\|2022-05-31...at 99.92% |
| 37 | 198450aa, >hMpxV/Brazil/RS-IAL-02/2022\|EPI ISL 13234112\|2022-05-31...at 99.53% |
| 38 | 198450aa, >hMpxV/Switzerland/ZH-UZH-IMV-3ba64538/2022\|EPI ISL 13251723\|2022-06-02...at 99.93% |
| 39 | 198450aa, >hMpxV/Brazil/SP-IAL-03/2022\|EPI ISL 13314740\|2022-06-11...at 99.87% |
| 40 | 198450aa, >hMpxV/Spain/AN-HUCSC-00001/2022\|EPI ISL 13339105\|2022-06-07...at 99.93% |
| 41 | 198450aa, >hMpxV/Brazil/SP-IAL-06/2022\|EPI ISL 13343718\|2022-06-15...at 99.90% |
| 42 | 198450aa, >hMpxV/Canada/un-NML-2969/2022\|EPI ISL 13408861\|2022-05-24...at 98.44% |
| 43 | 198450aa, >hMpxV/Brazil/SP-IAL-09/2022\|EPI ISL 13437056\|2022-06-20...at 99.86% |
| 44 | 198450aa, >hMpxV/Brazil/SP-IAL-14/2022\|EPI ISL 13508393\|2022-06-24...at 99.86% |
| 45 | 198450aa, >hMpxV/Canada/un-NML-3127/2022\|EPI ISL 13544226\|2022-05-30...at 98.44% |
| 46 | 198450aa, >hMpxV/Canada/un-NML-3137/2022\|EPI ISL 13544228\|2022-05-29...at 98.15% |
| 47 | 198450aa, >hMpxV/Canada/un-NML-3214/2022\|EPI ISL 13544229\|2022-05-30...at 98.44% |
| 48 | 198450aa, >hMpxV/Canada/un-NML-3292/2022\|EPI ISL 13544230\|2022-06-01...at 98.44% |
| 49 | 198450aa, >hMpxV/Canada/un-NML-3407/2022\|EPI ISL 13544231\|2022-06-04...at 98.42% |
| 50 | 198450aa, >hMpxV/Canada/un-NML-3467/2022\|EPI ISL 13544233\|2022-06-04...at 98.37% |
| 51 | 198450aa, >hMpxV/Canada/un-NML-3472/2022\|EPI ISL 13544234\|2022-06-02...at 98.44% |
| 52 | 198450aa, >hMpxV/Canada/un-NML-3476/2022\|EPI ISL 13544235\|2022-05-27...at 98.43% |
| 53 | 198450aa, >hMpxV/Canada/un-NML-2858/2022\|EPI ISL 13544239\|2022-05-19...at 98.12% |
| 54 | 198450aa, >hMpxV/Canada/un-NML-2898/2022\|EPI ISL 13544241\|2022-05-20...at 98.06% |
| 55 | 198450aa, >hMpxV/Canada/un-NML-3100/2022\|EPI ISL 13544244\|2022-05-27...at 98.44% |
| 56 | 198450aa, >hMpxV/Canada/un-NML-3141/2022\|EPI ISL 13544245\|2022-05-27...at 98.07% |
| 57 | 198450aa, >hMpxV/Canada/un-NML-3162/2022\|EPI ISL 13544248\|2022-05-29...at 98.43% |
| 58 | 198450aa, >hMpxV/Canada/un-NML-3165/2022\|EPI ISL 13544249\|2022-05-29...at 98.18% |
| 59 | 198450aa, >hMpxV/Canada/un-NML-3262/2022\|EPI ISL 13544252\|2022-05-30...at 98.40% |
| 60 | 198450aa, >hMpxV/Canada/un-NML-3272/2022\|EPI ISL 13544254\|2022-05-31...at 98.44% |
| 61 | 198450aa, >hMpxV/Canada/un-NML-3273/2022\|EPI ISL 13544255\|2022-05-31...at 98.12% |
| 62 | 198450aa, >hMpxV/Canada/un-NML-3336/2022\|EPI ISL 13544256\|2022-06-01...at 98.43% |
| 63 | 198450aa, >hMpxV/Canada/un-NML-3339/2022\|EPI ISL 13544257\|2022-06-01...at 98.43% |
| 64 | 198450aa, >hMpxV/Canada/un-NML-3342/2022\|EPI ISL 13544258\|2022-06-01...at 98.42% |
| 65 | 198450aa, >hMpxV/Canada/un-NML-3348/2022\|EPI ISL 13544260\|2022-05-31...at 98.43% |
| 66 | 198450aa, >hMpxV/Canada/un-NML-3357/2022\|EPI ISL 13544261\|2022-06-02...at 98.43% |
| 67 | 198450aa, >hMpxV/Canada/un-NML-3366/2022\|EPI ISL 13544263\|2022-06-02...at 98.43% |
| 68 | 198450aa, >hMpxV/Canada/un-NML-3368/2022\|EPI ISL 13544264\|2022-06-02...at 98.42% |
| 69 | 198450aa, >hMpxV/Canada/un-NML-3441/2022\|EPI ISL 13544265\|2022-06-02...at 98.41% |
| 70 | 198450aa, >hMpxV/Canada/un-NML-3448/2022\|EPI ISL 13544266\|2022-06-02...at 98.43% |
| 71 | 198450aa, >hMpxV/Canada/un-NML-3450/2022\|EPI ISL 13544267\|2022-06-02...at 98.44% |
| 72 | 198450aa, >hMpxV/Netherlands/un-EMC-NL006/2022\|EPI ISL 13658019\|2022-06-23...at 95.09% |
| 73 | 198450aa, >hMpxV/Netherlands/NH-AUMC-0001/2022\|EPI ISL 13728303\|2022-06-21...at 95.21% |
| 74 | 198450aa, >hMpxV/Brazil/SP-IAL-18/2022\|EPI ISL 13732932\|2022-07-06...at 99.92% |
| 75 | 198450aa, >hMpxV/Peru/LIM-INS-005/2022\|EPI ISL 13833194\|2022-07-01...at 95.37% |
| 76 | 198450aa, >hMpxV/Austria/MUW 1531254/2022\|EPI ISL 13842548\|2022-07-04...at 95.37% |
| 77 | 198450aa, >hMpxV/Canada/un-NML-3657/2022\|EPI ISL 13908340\|2022-06-10...at 98.39% |
| 78 | 198450aa, >hMpxV/Canada/un-NML-3658/2022\|EPI ISL 13908341\|2022-06-10...at 98.36% |
| 79 | 198450aa, >hMpxV/Canada/un-NML-3672/2022\|EPI ISL 13908342\|2022-06-11...at 98.24% |
| 80 | 198450aa, >hMpxV/Canada/un-NML-3674/2022\|EPI ISL 13908343\|2022-06-13...at 98.29% |
| 81 | 198450aa, >hMpxV/England/UKHSA-5/2022\|EPI ISL 13958697\|2022-05-16...at 99.88% |
| 82 | 198450aa, >hMpxV/USA/WA-UW-0011/2022\|EPI ISL 14033204\|2022-07...at 99.79% |
| 83 | 198450aa, >hMpxV/USA/WA-UW-0012/2022\|EPI ISL 14033205\|2022-07...at 99.93% |
| 84 | 198450aa, >hMpxV/USA/WA-UW-0014/2022\|EPI ISL 14033206\|2022-07...at 99.93% |
| 85 | 198450aa, >hMpxV/USA/WA-UW-0015/2022\|EPI ISL 14033207\|2022-07...at 99.93% |
| 86 | 198450aa, >hMpxV/USA/WA-UW-0016/2022\|EPI ISL 14033208\|2022-07...at 99.93% |
| 87 | 198450aa, >hMpxV/USA/WA-UW-0018/2022\|EPI ISL 14033209\|2022-07...at 99.93% |
| 88 | 198450aa, >hMpxV/USA/WA-UW-0020/2022\|EPI ISL 14033210\|2022-07...at 99.93% |
| 89 | 198450aa, >hMpxV/USA/WA-UW-0021/2022\|EPI ISL 14033211\|2022-07...at 99.93% |
| 90 | 198450aa, >hMpxV/USA/WA-UW-0022/2022\|EPI ISL 14033212\|2022-07...at 99.93% |
| 91 | 198450aa, >hMpxV/USA/WA-UW-0023/2022\|EPI ISL 14033213\|2022-07...at 99.93% |
| 92 | 198450aa, >hMpxV/Peru/LIM-INS-012/2022\|EPI ISL 14207727\|2022-07-06...at 95.36% |
| 93 | 198450aa, >hMpxV/Peru/LIM-INS-014/2022\|EPI ISL 14207729\|2022-07-09...at 95.36% |
| 94 | 198450aa, >hMpxV/Netherlands/un-EMC-NL017/2022\|EPI ISL 14254435\|2022-07-29...at 95.07% |
| 95 | 198450aa, >hMpxV/Netherlands/un-EMC-NL019/2022\|EPI ISL 14254437\|2022-07-25...at 95.08% |
| 96 | 198450aa, >hMpxV/Portugal/INSA-PT0019/2022\|EPI ISL 13056901\|2022-05-21...at 98.32% |
| 97 | 198450aa, >hMpxV/Portugal/INSA-PT0018/2022\|EPI ISL 13056902\|2022-05-20...at 98.43% |
| 98 | 198450aa, >hMpxV/Portugal/INSA-PT0012/2022\|EPI ISL 13056903\|2022-05-23...at 98.43% |
| 99 | 198450aa, >hMpxV/Portugal/INSA-PT0024/2022\|EPI ISL 13056904\|2022-05-19...at 98.44% |
| 100 | 198450aa, >hMpxV/Portugal/INSA-PT0021/2022\|EPI ISL 13056905\|2022-05-19...at 98.44% |
| 101 | 198450aa, >hMpxV/Portugal/INSA-PT0022/2022\|EPI ISL 13056906\|2022-05-19...at 98.44% |
| 102 | 198450aa, >hMpxV/Portugal/INSA-PT0013/2022\|EPI ISL 13056907\|2022-05-20...at 98.43% |
| 103 | 198450aa, >hMpxV/Portugal/INSA-PT0014/2022\|EPI ISL 13056908\|2022-05-23...at 98.44% |
| 104 | 198450aa, >hMpxV/Portugal/INSA-PT0011/2022\|EPI ISL 13056909\|2022-05-23...at 98.44% |
| 105 | 198450aa, >hMpxV/USA/FL-DHCPPCDC-001/2022\|EPI ISL 13094227\|2022-05-22...at 99.07% |
| 106 | 198450aa, >hMpxV/USA/CA-DHCPPCDC-003/2022\|EPI ISL 13100618\|2022-05...at 95.27% |
| 107 | 198450aa, >hMpxV/USA/FL-DHCPPCDC-004/2022\|EPI ISL 13100619\|2022-05...at 99.94% |
| 108 | 198450aa, >hMpxV/USA/UT-DHCPPCDC-005/2022\|EPI ISL 13100620\|2022-05...at 99.93% |
| 109 | 198450aa, >hMpxV/USA/UT-DHCPPCDC-006/2022\|EPI ISL 13100719\|2022-05...at 95.22% |
| 110 | 198450aa, >hMpxV/Spain/CT-HUVH-60425/2022\|EPI ISL 13363142\|2022-06-10...at 98.44% |
| 111 | 198450aa, >hMpxV/Scotland/CVR-1a/2022\|EPI ISL 13409177\|2022-05-15...at 95.33% |
| 112 | 198450aa, >hMpxV/Scotland/CVR-1b/2022\|EPI ISL 13409178\|2022-05-15...at 95.33% |
| 113 | 198450aa, >hMpxV/Scotland/CVR-1c/2022\|EPI ISL 13409179\|2022-05-15...at 95.33% |
| 114 | 198450aa, >hMpxV/Brazil/SP-IAL-07/2022\|EPI ISL 13436658\|2022-06-14...at 99.88% |
| 115 | 198450aa, >hMpxV/Spain/MD-HULP-8887/2022\|EPI ISL 13449965\|2022-06-06...at 99.94% |
| 116 | 198450aa, >hMpxV/Spain/MD-HULP-4061/2022\|EPI ISL 13449966\|2022-05-20...at 99.87% |
| 117 | 198450aa, >hMpxV/Brazil/SP-IAL-15/2022\|EPI ISL 13508471\|2022-06-24...at 99.86% |
| 118 | 198450aa, >hMpxV/Austria/MUW 1531254/2022\|EPI ISL 13842548\|2022-07-04...at 95.36% |
| 119 | 198450aa, >hMpxV/Taiwan/CVDCDC-110-231642/2022\|EPI ISL 13632071\|2022-06-27...at 99.87% |
| 120 | 198450aa, >hMpxV/Brazil/SP-IAL-16/2022\|EPI ISL 13705358\|2022-07-04...at 99.85% |
| 121 | 198450aa, >hMpxV/Brazil/SP-IAL-17/2022\|EPI ISL 13705407\|2022-07-04...at 99.93% |
| 122 | 198450aa, >hMpxV/Netherlands/un-EMC-NL010/2022\|EPI ISL 13822669\|2022-06-27...at 95.16% |
| 123 | 198450aa, >hMpxV/Netherlands/un-EMC-NL011/2022\|EPI ISL 13822718\|2022-06-24...at 95.20% |
| 124 | 198450aa, >hMpxV/Canada//un-NML-3393/2022\|EPI ISL 13827274\|2022-06-03...at 98.44% |
| 125 | 198450aa, >hMpxV/Canada//un-NML-3530/2022\|EPI ISL 13827275\|2022-06-03...at 98.38% |
| 126 | 198450aa, >hMpxV/Canada//un-NML-3531/2022\|EPI ISL 13827277\|2022-06-03...at 98.44% |
| 127 | 198450aa, >hMpxV/Canada//un-NML-3532/2022\|EPI ISL 13827278\|2022-06-07...at 98.43% |
| 128 | 198450aa, >hMpxV/Canada//un-NML-3558/2022\|EPI ISL 13827279\|2022-06-09...at 98.03% |
| 129 | 198450aa, >hMpxV/Canada//un-NML-3686/2022\|EPI ISL 13827280\|2022-06-14...at 98.28% |
| 130 | 198450aa, >hMpxV/Canada//un-NML-3702/2022\|EPI ISL 13827281\|2022-06-15...at 98.45% |
| 131 | 198450aa, >hMpxV/Canada//un-NML-3725/2022\|EPI ISL 13827282\|2022-06-16...at 98.43% |
| 132 | 198450aa, >hMpxV/Austria/MUW 1531254/2022\|EPI ISL 13842548\|2022-07-04...at 95.36% |
| 133 | 198450aa, >hMpxV/USA/CA-CDPH-000001/2022\|EPI ISL 13993734\|2022-06-02...at 99.86% |
| 134 | 198450aa, >hMpxV/USA/CA-CDPH-000002/2022\|EPI ISL 13993735\|2022-06-02...at 99.93% |
| 135 | 198450aa, >hMpxV/USA/CA-CDPH-000003/2022\|EPI ISL 13993736\|2022-06-04...at 99.60% |
| 136 | 198450aa, >hMpxV/USA/CA-CDPH-000004/2022\|EPI ISL 13993737\|2022-06-04...at 99.93% |
| 137 | 198450aa, >hMpxV/USA/CA-CDPH-000005/2022\|EPI ISL 13993738\|2022-06-07...at 99.93% |
| 138 | 198450aa, >hMpxV/USA/CA-CDPH-000006/2022\|EPI ISL 13993739\|2022-06-07...at 99.93% |
| 139 | 198450aa, >hMpxV/Canada/un-NML-3817/2022\|EPI ISL 14050451\|2022-06-16...at 99.92% |
| 140 | 198450aa, >hMpxV/Canada/un-NML-3907/2022\|EPI ISL 14050453\|2022-06-27...at 99.79% |
| 141 | 198450aa, >hMpxV/Canada/un-NML-3909/2022\|EPI ISL 14050454\|2022-06-27...at 99.93% |
| 142 | 198450aa, >hMpxV/Canada/un-NML-3936/2022\|EPI ISL 14050458\|2022-06-30...at 99.48% |
| 143 | 198450aa, >hMpxV/Brazil/SP-IAL-22/2022\|EPI ISL 14070493\|2022-07-19...at 99.92% |
| 144 | 198450aa, >hMpxV/Austria/MUW-1533374/2022\|EPI ISL 14166709\|2022-07-11...at 99.94% |
| 145 | 198450aa, >hMpxV/Peru/LIM-INS-009/2022\|EPI ISL 14207724\|2022-07-01...at 95.34% |
| 146 | 198450aa, >hMpxV/Peru/LIM-INS-011/2022\|EPI ISL 14207726\|2022-07-04...at 95.36% |
| 147 | 198450aa, >hMpxV/Peru/LIM-INS-015/2022\|EPI ISL 14207730\|2022-07-09...at 95.37% |
| 148 | 198450aa, >hMpxV/Peru/LIM-INS-016/2022\|EPI ISL 14207731\|2022-07-09...at 95.36% |
| 149 | 198450aa, >hMpxV/Peru/LIM-INS-017/2022\|EPI ISL 14207732\|2022-07-08...at 95.36% |
| 150 | 198450aa, >hMpxV/Peru/LIM-INS-018/2022\|EPI ISL 14207733\|2022-07-09...at 95.37% |
| 151 | 198450aa, >hMpxV/Peru/LIM-INS-019/2022\|EPI ISL 14207734\|2022-07-11...at 95.36% |
| 152 | 198450aa, >hMpxV/Peru/LIM-INS-020/2022\|EPI ISL 14207735\|2022-07-12...at 95.36% |
| 153 | 198450aa, >hMpxV/Peru/LIM-INS-021/2022\|EPI ISL 14207736\|2022-07-12...at 95.36% |
| 154 | 198450aa, >hMpxV/Peru/LAL-INS-022/2022\|EPI ISL 14207737\|2022-07-12...at 95.36% |
| 155 | 198450aa, >hMpxV/Peru/TAC-INS-023/2022\|EPI ISL 14207738\|2022-07-12...at 95.37% |
| 156 | 198450aa, >hMpxV/Peru/LAL-INS-024/2022\|EPI ISL 14207739\|2022-07-15...at 95.36% |
| 157 | 198450aa, >hMpxV/Peru/LAL-INS-025/2022\|EPI ISL 14207740\|2022-07-15...at 95.36% |
| 158 | 198450aa, >hMpxV/Peru/LIM-INS-026/2022\|EPI ISL 14207741\|2022-07-15...at 95.36% |
| 159 | 198450aa, >hMpxV/USA/un-UW-0024/2022\|EPI ISL 14216746\|2022-07...at 99.93% |
| 160 | 198450aa, >hMpxV/USA/un-UW-0025/2022\|EPI ISL 14216748\|2022-07...at 99.92% |
| 161 | 198450aa, >hMpxV/USA/un-UW-0026/2022\|EPI ISL 14216750\|2022-07...at 99.93% |
| 162 | 198450aa, >hMpxV/USA/un-UW-0027/2022\|EPI ISL 14216752\|2022-07...at 99.93% |
| 163 | 198450aa, >hMpxV/USA/un-UW-0028/2022\|EPI ISL 14216753\|2022-07...at 99.93% |
| 164 | 198450aa, >hMpxV/USA/un-UW-0029/2022\|EPI ISL 14216754\|2022-07...at 99.93% |
| 165 | 198450aa, >hMpxV/USA/un-UW-0030/2022\|EPI ISL 14216755\|2022-07...at 99.93% |
| 166 | 198450aa, >hMpxV/USA/un-UW-0031/2022\|EPI ISL 14216756\|2022-07...at 99.93% |
| 167 | 198450aa, >hMpxV/USA/un-UW-0032/2022\|EPI ISL 14216757\|2022-07...at 99.93% |
| 168 | 198450aa, >hMpxV/USA/un-UW-0033/2022\|EPI ISL 14216758\|2022-07...at 99.94% |
| 169 | 198450aa, >hMpxV/USA/un-UW-0034/2022\|EPI ISL 14216759\|2022-07...at 99.93% |
| 170 | 198450aa, >hMpxV/USA/un-UW-0035/2022\|EPI ISL 14216761\|2022-07...at 99.93% |
| 171 | 198450aa, >hMpxV/USA/un-UW-0036/2022\|EPI ISL 14216762\|2022-07...at 99.93% |
| 172 | 198450aa, >hMpxV/USA/un-UW-0037/2022\|EPI ISL 14216764\|2022-07...at 99.93% |
| 173 | 198450aa, >hMpxV/USA/un-UW-0038/2022\|EPI ISL 14216767\|2022-07...at 99.93% |
| 174 | 198450aa, >hMpxV/USA/un-UW-0039/2022\|EPI ISL 14216769\|2022-07...at 99.78% |
| 175 | 198450aa, >hMpxV/Chile/RM-ISP-75625/2022\|EPI ISL 14224334\|2022-06-16...at 99.93% |
| >Cluster 4 | |
| 0 | 198450aa, >hMpxV/France/un-IRBA-11/2022\|EPI ISL 13308158\|2022-05-19...^#^ |
| 1 | 198450aa, >hMpxV/France/un-IRBA-14/2022\|EPI ISL 13308160\|2022-05-20...at 98.59% |
| >Cluster 5 | |
| 0 | 198450aa, >hMpxV/Slovenia/UL-S3/2022\|EPI ISL 13308162\|2022-06...^#^ |
| >Cluster 6 | |
| 0 | 198450aa, >hMpxV/Slovenia/UL-S4/2022\|EPI ISL 13308163\|2022-06...^#^ |
| 1 | 198450aa, >hMpxV/Slovenia/UL-S5/2022\|EPI ISL 13308165\|2022-06...at 99.96% |
| 2 | 198450aa, >hMpxV/Slovenia/UL-S1-VE6/2022\|EPI ISL 13308167\|2022-05-23...at 99.92% |
| 3 | 198450aa, >hMpxV/USA/NY-CDC-003/2022\|EPI ISL 13744898\|2022-05...at 98.70% |
| 4 | 198450aa, >hMpxV/USA/IL-CDC-001/2022\|EPI ISL 13744899\|2022-05...at 98.71% |
| 5 | 198450aa, >hMpxV/Ecuador/INSPI 1104-0722 EGTV-G/2022\|EPI ISL 13983356\|2022-07-04...at 95.63% |
| 6 | 198450aa, >hMpxV/USA/CA-CDC-PRB-007/2022\|EPI ISL 14244555\|2022-06...at 98.71% |
| 7 | 198450aa, >hMpxV/USA/CA-CDC-PRB-006/2022\|EPI ISL 14244556\|2022-06...at 98.70% |
| 8 | 198450aa, >hMpxV/USA/GA-CDC-PRB-001/2022\|EPI ISL 14244557\|2022-05...at 98.70% |
| 9 | 198450aa, >hMpxV/USA/PA-CDC-PRB-001/2022\|EPI ISL 14244558\|2022-05...at 98.73% |
| 10 | 198450aa, >hMpxV/Belgium/UZ REGA-6/2022\|EPI ISL 13537926\|2022-05-22...at 95.20% |
| 11 | 198450aa, >hMpxV/Belgium/UZ REGA-6/2022\|EPI ISL 13537926\|2022-05-22...at 95.17% |
| 12 | 198450aa, >hMpxV/Slovenia/UL-2/2022\|EPI ISL 13052290\|2022-05-24...at 99.92% |
| 13 | 198450aa, >hMpxV/Slovenia/UL-1/2022\|EPI ISL 13052291\|2022-05-23...at 99.93% |
| 14 | 198450aa, >hMpxV/Belgium/UZ REGA-6/2022\|EPI ISL 13537926\|2022-05-22...at 98.58% |
| 15 | 198450aa, >hMpxV/Belgium/UZ REGA-6/2022\|EPI ISL 13537926\|2022-05-22...at 98.58% |
| 16 | 198450aa, >hMpxV/Belgium/UZ REGA-6/2022\|EPI ISL 13537926\|2022-05-22...at 98.68% |
| 17 | 198450aa, >hMpxV/Belgium/UZ REGA-6/2022\|EPI ISL 13537926\|2022-05-22...at 98.61% |
| 18 | 198450aa, >hMpxV/USA/IL-CDC-003/2022\|EPI ISL 13744896\|2022-06...at 98.68% |
| 19 | 198450aa, >hMpxV/USA/NY-CDC-002/2022\|EPI ISL 13744897\|2022-05...at 98.68% |
| 20 | 198450aa, >hMpxV/Canada/BC-BCCDC-6798-56876-06/2022\|EPI ISL 13351002\|2022-06...at 98.70% |
| 21 | 198450aa, >hMpxV/Slovenia/UL-S7/2022\|EPI ISL 13445553\|2022-06...at 99.98% |
| 22 | 198450aa, >hMpxV/USA/CA-CDC-002/2022\|EPI ISL 13734270\|2022-05...at 98.68% |
| 23 | 198450aa, >hMpxV/USA/NY-CDC-004/2022\|EPI ISL 13744900\|2022-05...at 98.69% |
| 24 | 198450aa, >hMpxV/USA/NY-CDC-005/2022\|EPI ISL 13744901\|2022-06...at 95.29% |
| >Cluster 7 | |
| 0 | 198450aa, >hMpxV/Georgia/NCDC-001/2022\|EPI ISL 13331598\|2022-06-04...^#^ |
| >Cluster 8 | |
| 0 | 198450aa, >hMpxV/Canada/un-NML-2930/2022\|EPI ISL 13408801\|2022-05-21...^#^ |
| 1 | 198450aa, >hMpxV/Canada/un-NML-2836/2022\|EPI ISL 13408807\|2022-05-17...at 99.80% |
| 2 | 198450aa, >hMpxV/Canada/un-NML-2837/2022\|EPI ISL 13408809\|2022-05-18...at 99.76% |
| 3 | 198450aa, >hMpxV/Canada/un-NML-2844/2022\|EPI ISL 13408811\|2022-05-14...at 99.97% |
| 4 | 198450aa, >hMpxV/Canada/un-NML-2846/2022\|EPI ISL 13408813\|2022-05-19...at 99.95% |
| 5 | 198450aa, >hMpxV/Canada/un-NML-2849/2022\|EPI ISL 13408815\|2022-05-19...at 99.93% |
| 6 | 198450aa, >hMpxV/Canada/un-NML-2850/2022\|EPI ISL 13408817\|2022-05-19...at 99.93% |
| 7 | 198450aa, >hMpxV/Canada/un-NML-2851/2022\|EPI ISL 13408819\|2022-05-19...at 99.72% |
| 8 | 198450aa, >hMpxV/Canada/un-NML-2852/2022\|EPI ISL 13408821\|2022-05-19...at 99.95% |
| 9 | 198450aa, >hMpxV/Canada/un-NML-2853/2022\|EPI ISL 13408823\|2022-05-19...at 99.93% |
| 10 | 198450aa, >hMpxV/Canada/un-NML-2884/2022\|EPI ISL 13408837\|2022-05-20...at 99.99% |
| 11 | 198450aa, >hMpxV/Canada/un-NML-2893/2022\|EPI ISL 13408843\|2022-05-20...at 99.98% |
| 12 | 198450aa, >hMpxV/Canada/un-NML-2896/2022\|EPI ISL 13408847\|2022-05-20...at 99.99% |
| 13 | 198450aa, >hMpxV/Italy/PIE-OAS-02530345/2022\|EPI ISL 13502582\|2022-06-13...at 99.81% |
| 14 | 198450aa, >hMpxV/India/KL-ICMR-21-5432-557/2022\|EPI ISL 13953611\|2022-07-16...at 98.07% |
| 15 | 198450aa, >hMpxV/India/KL-ICMR-16-5316-570-P1/2022\|EPI ISL 14049244\|2022-07-13...at 98.29% |
| 16 | 198450aa, >hMpxV/India/KL-ICMR-16-5316-573-P2/2022\|EPI ISL 14049245\|2022-07-13...at 98.41% |
| 17 | 198450aa, >hMpxV/Portugal/INSA-PT0003/2022\|EPI ISL 13052266\|2022-05-15...at 99.94% |
| 18 | 198450aa, >hMpxV/Portugal/INSA-PT0004/2022\|EPI ISL 13052267\|2022-05-17...at 99.56% |
| 19 | 198450aa, >hMpxV/Portugal/INSA-PT0006/2022\|EPI ISL 13052268\|2022-05-15...at 99.98% |
| 20 | 198450aa, >hMpxV/Portugal/INSA-PT0007/2022\|EPI ISL 13052269\|2022-05-15...at 99.92% |
| 21 | 198450aa, >hMpxV/Portugal/INSA-PT0009/2022\|EPI ISL 13052270\|2022-05-15...at 99.97% |
| 22 | 198450aa, >hMpxV/Portugal/INSA-PT0005/2022\|EPI ISL 13052272\|2022-05-15...at 96.48% |
| 23 | 198450aa, >hMpxV/Portugal/INSA-PT0008/2022\|EPI ISL 13052273\|2022-05-15...at 96.55% |
| 24 | 198450aa, >hMpxV/Switzerland/un-UHG-38134631/2022\|EPI ISL 13052274\|2022-05-19...at 99.76% |
| 25 | 198450aa, >hMpxV/Italy/LOM-AMC-2205251-DS/2022\|EPI ISL 13302316\|2022-05-25...at 99.81% |
| 26 | 198450aa, >hMpxV/Spain/un-ITER-0001b/2022\|EPI ISL 13331717\|2022-05-31...at 99.91% |
| 27 | 198450aa, >hMpxV/Canada/un-NML-2928/2022\|EPI ISL 13408799\|2022-05-21...at 99.99% |
| 28 | 198450aa, >hMpxV/Canada/un-NML-2861/2022\|EPI ISL 13408827\|2022-05-18...at 99.90% |
| 29 | 198450aa, >hMpxV/Canada/un-NML-2873/2022\|EPI ISL 13408831\|2022-05-19...at 99.99% |
| 30 | 198450aa, >hMpxV/Canada/un-NML-2874/2022\|EPI ISL 13408833\|2022-05-19...at 99.99% |
| 31 | 198450aa, >hMpxV/Canada/un-NML-2883/2022\|EPI ISL 13408835\|2022-05-20...at 99.96% |
| 32 | 198450aa, >hMpxV/Canada/un-NML-2897/2022\|EPI ISL 13408849\|2022-05-20...at 99.97% |
| 33 | 198450aa, >hMpxV/Canada/un-NML-2899/2022\|EPI ISL 13408851\|2022-05-20...at 99.94% |
| 34 | 198450aa, >hMpxV/Canada/un-NML-2955/2022\|EPI ISL 13408855\|2022-05-23...at 99.99% |
| 35 | 198450aa, >hMpxV/Canada/un-NML-2967/2022\|EPI ISL 13408857\|2022-05-16...at 99.99% |
| 36 | 198450aa, >hMpxV/Portugal/INSA-PT0033/2022\|EPI ISL 13466448\|2022-05-24...at 99.93% |
| 37 | 198450aa, >hMpxV/Portugal/INSA-PT0038/2022\|EPI ISL 13466449\|2022-05-24...at 99.94% |
| 38 | 198450aa, >hMpxV/Portugal/INSA-PT0036/2022\|EPI ISL 13466450\|2022-05-26...at 99.91% |
| 39 | 198450aa, >hMpxV/Portugal/INSA-PT0032/2022\|EPI ISL 13466451\|2022-05-26...at 99.96% |
| 40 | 198450aa, >hMpxV/Portugal/INSA-PT0034/2022\|EPI ISL 13466452\|2022-05-26...at 99.73% |
| 41 | 198450aa, >hMpxV/Portugal/INSA-PT0031/2022\|EPI ISL 13466453\|2022-05-26...at 99.98% |
| 42 | 198450aa, >hMpxV/Portugal/INSA-PT0035/2022\|EPI ISL 13466455\|2022-05-27...at 99.97% |
| 43 | 198450aa, >hMpxV/Portugal/INSA-PT0029/2022\|EPI ISL 13466456\|2022-05-27...at 99.97% |
| 44 | 198450aa, >hMpxV/Portugal/INSA-PT0046/2022\|EPI ISL 13466457\|2022-05-30...at 99.96% |
| 45 | 198450aa, >hMpxV/Portugal/INSA-PT0041/2022\|EPI ISL 13466459\|2022-06-02...at 99.83% |
| 46 | 198450aa, >hMpxV/Portugal/INSA-PT0047/2022\|EPI ISL 13466460\|2022-06-02...at 99.91% |
| 47 | 198450aa, >hMpxV/Portugal/INSA-PT0040/2022\|EPI ISL 13466461\|2022-06-02...at 99.89% |
| 48 | 198450aa, >hMpxV/South Africa/NICD-SVPL232/2022\|EPI ISL 13632288\|2022-06-24...at 99.82% |
| 49 | 198450aa, >hMpxV/Canada/un-NML-2870/2022\|EPI ISL 13544240\|2022-05-21...at 99.84% |
| 50 | 198450aa, >hMpxV/Germany/BE-ChVir28136/2022\|EPI ISL 13889442\|2022-05-25...at 99.71% |
| 51 | 198450aa, >hMpxV/USA/MD-DHCPPCDC-008/2021\|EPI ISL 13100622\|2021-11...at 95.40% |
| 52 | 198450aa, >hMpxV/Brazil/SP-IAL-01/2022\|EPI ISL 13191438\|2022-06-07...at 96.57% |
| 53 | 198450aa, >hMpxV/Hungary/NBL-003/2022\|EPI ISL 13374487\|2022-06-01...at 99.95% |
| 54 | 198450aa, >hMpxV/South Africa/NICD-SVPL232/2022\|EPI ISL 13632288\|2022-06-24...at 99.91% |
| >Cluster 9 | |
| 0 | 198450aa, >hMpxV/Peru/LIM-INS-002/2022\|EPI ISL 13651348\|2022-06-27...^#^ |
| >Cluster 10 | |
| 0 | 198450aa, >hMpxV/Luxembourg/LNS-0899423/2022\|EPI ISL 13660191\|2022-06-10...^#^ |
| >Cluster 11 | |
| 0 | 198450aa, >hMpxV/India/KL-ICMR-16-5316-553/2022\|EPI ISL 13953610\|2022-07-13...^#^ |
| >Cluster 12 | |
| 0 | 198450aa, >hMpxV/Netherlands/un-EMC-NL013/2022\|EPI ISL 14170201\|2022-07-11...^#^ |
| 1 | 198450aa, >hMpxV/Spain/un-ISCIII-407/2022\|EPI ISL 14181960\|2022...at 98.99% |
| 2 | 198450aa, >hMpxV/Netherlands/un-EMC-NL007/2022\|EPI ISL 13658021\|2022-06-23...at 99.47% |
| 3 | 198450aa, >hMpxV/Germany/BE-ChVir28656/2022\|EPI ISL 13889435\|2022-06-21...at 98.76% |
| 4 | 198450aa, >hMpxV/Germany/BE-ChVir28692/2022\|EPI ISL 13890204\|2022-06-25...at 98.57% |
| 5 | 198450aa, >hMpxV/Germany/BE-ChVir28389/2022\|EPI ISL 13890482\|2022-06-08...at 97.25% |
| 6 | 198450aa, >hMpxV/Slovakia/PHA-CU-0802 34042/2022\|EPI ISL 14211645\|2022-07-20...at 99.02% |
| 7 | 198450aa, >hMpxV/Netherlands/un-EMC-NL018/2022\|EPI ISL 14254436\|2022-07-22...at 99.54% |
| 8 | 198450aa, >hMpxV/Netherlands/un-EMC-NL020/2022\|EPI ISL 14254438\|2022-07-18...at 99.23% |
| 9 | 198450aa, >hMpxV/Netherlands/un-EMC-NL003/2022\|EPI ISL 13244349\|2022-06-03...at 99.12% |
| >Cluster 13 | |
| 0 | 198450aa, >hMpxV/Thailand/NIC PKT-M1/2022\|EPI ISL 14295679\|2022-08-02...^#^ |
| 1 | 198450aa, >hMpxV/England/UKHSA-3/2022\|EPI ISL 13052280\|2022-05...at 99.57% |
| 2 | 198450aa, >hMpxV/Germany/BE-ChVir28700/2022\|EPI ISL 13890466\|2022-06-24...at 99.50% |
| 3 | 198450aa, >hMpxV/Germany/BE-ChVir28601/2022\|EPI ISL 13890479\|2022-06-17...at 99.41% |
| >Cluster 14 | |
| 0 | 198450aa, >hMpxV/France/un-UT-67/2022\|EPI ISL 13052275\|2022-05...^#^ |
| >Cluster 15 | |
| 0 | 198450aa, >hMpxV/Germany/un-RKI-01/2022\|EPI ISL 13052293\|2022-05...^#^ |
| 1 | 198450aa, >hMpxV/Germany/un-RKI-02/2022\|EPI ISL 13052294\|2022-05...at 99.97% |
| >Cluster 16 | |
| 0 | 198450aa, >hMpxV/Italy/FVG-AreaSP-01/2022\|EPI ISL 13052295\|2022-05-25...^#^ |
| 1 | 198450aa, >hMpxV/Brasil/SP-SPH-0002/2022\|EPI ISL 13537922\|2022-06-14...at 99.85% |
| 2 | 198450aa, >hMpxV/Brazil/SP-SPH-0001/2022\|EPI ISL 13270980\|2022-06-07...at 99.59% |
| >Cluster 17 | |
| 0 | 198450aa, >hMpxV/Hungary/NBL-001/2022\|EPI ISL 13304977\|2022-05-30...^#^ |
| >Cluster 18 | |
| 0 | 198450aa, >hMpxV/Belgium/ITM pt 07/2022\|EPI ISL 14241409\|2022-05-30...^#^ |
| 1 | 198450aa, >hMpxV/Belgium/ITM pt 07/2022\|EPI ISL 14241409\|2022-05-30...at 98.44% |
| 2 | 198450aa, >hMpxV/Belgium/ITM pt 07/2022\|EPI ISL 14241409\|2022-05-30...at 98.39% |
| 3 | 198450aa, >hMpxV/Belgium/ITM pt 07/2022\|EPI ISL 14241409\|2022-05-30...at 98.61% |
| 4 | 198450aa, >hMpxV/Belgium/ITM pt 07/2022\|EPI ISL 14241409\|2022-05-30...at 98.47% |
| 5 | 198450aa, >hMpxV/Belgium/ITM pt 07/2022\|EPI ISL 14241409\|2022-05-30...at 98.49% |
| >Cluster 19 | |
| 0 | 198450aa, >hMpxV/Germany/un-RKI-03/2022\|EPI ISL 13117291\|2022-05...^#^ |
| >Cluster 20 | |
| 0 | 198450aa, >hMpxV/Belgium/ITM-07/2022\|EPI ISL 13338028\|2022-05-30...^#^ |
| 1 | 198450aa, >hMpxV/Belgium/ITM pt 07/2022\|EPI ISL 14241409\|2022-05-30...at 100.00% |
| >Cluster 21 | |
| 0 | 198450aa, >hMpxV/Poland/NIZP-PZH-0001/2022\|EPI ISL 13472080\|2022-06-06...^#^ |
| >Cluster 22 | |
| 0 | 198450aa, >hMpxV/Mexico/CMX-InDRE-IBT-001/2022\|EPI ISL 13624509\|2022-05-27...^#^ |
| >Cluster 23 | |
| 0 | 198450aa, >hMpxV/Belgium/ITM-31/2022\|EPI ISL 13734269\|2022-05-27...^#^ |

**Notes**:

* There are 24 clusters in total, because the number starts from 0. Percentage after the genome indicates the identity between it and the unique genome retained in this cluster.

^#^ These genomes are retained and the rest are discarded as redundant one(s) when doing phylogenetic analysis, but all genomes are included in the polymorphism study.
