# Supplementary figures and images for: Monkeypox virus 2022, gene heterogeneity and protein polymorphism
Source: Signal Transduct Target Ther. 2023 Jul 17;8:278. doi: 10.1038/s41392-023-01540-2 (PMC10352349; doi:10.1038/s41392-023-01540-2)

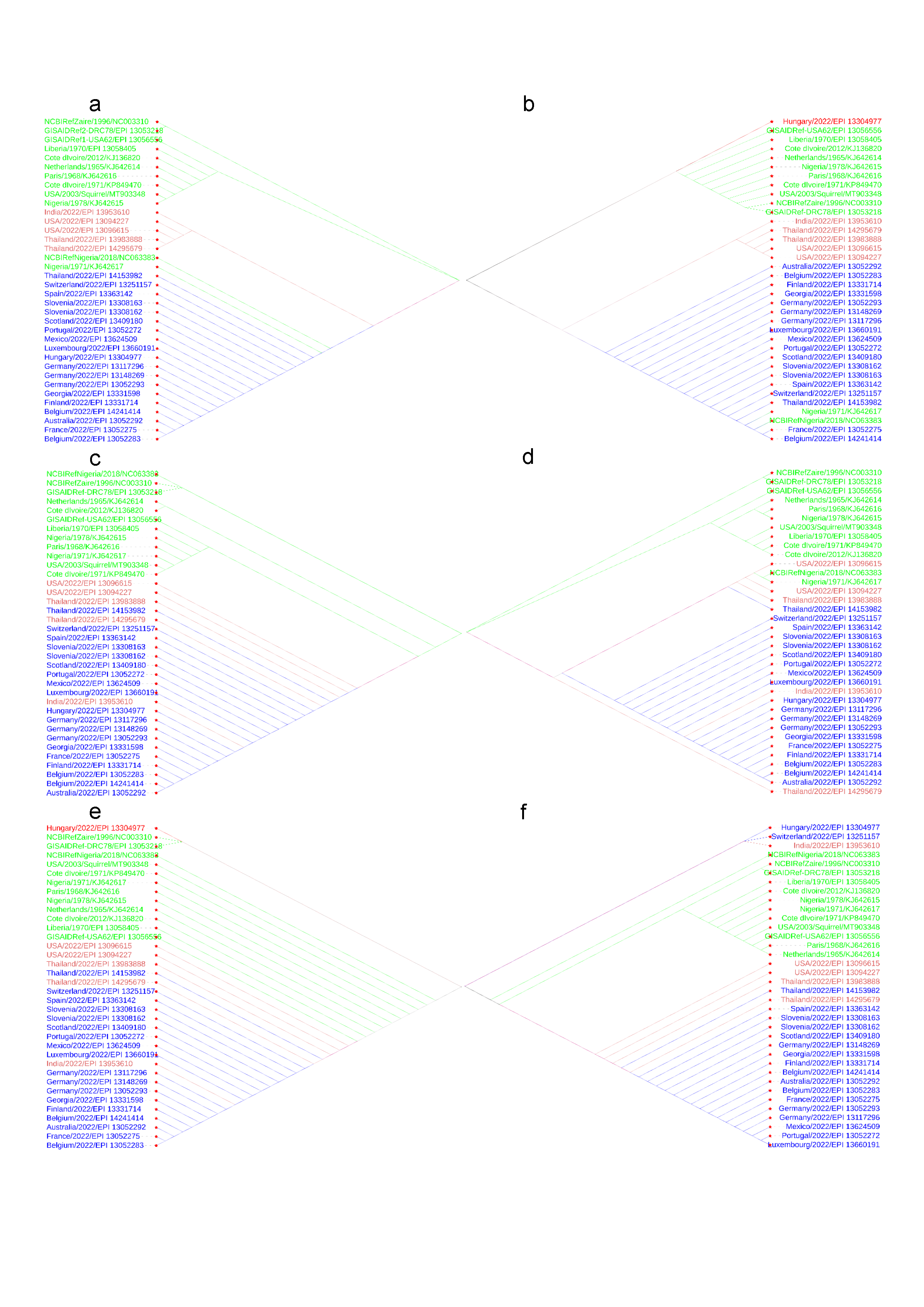

Supplement: Supplementary file 3 — Supplementary Fig.1 [file 41392_2023_1540_MOESM3_ESM.tif]

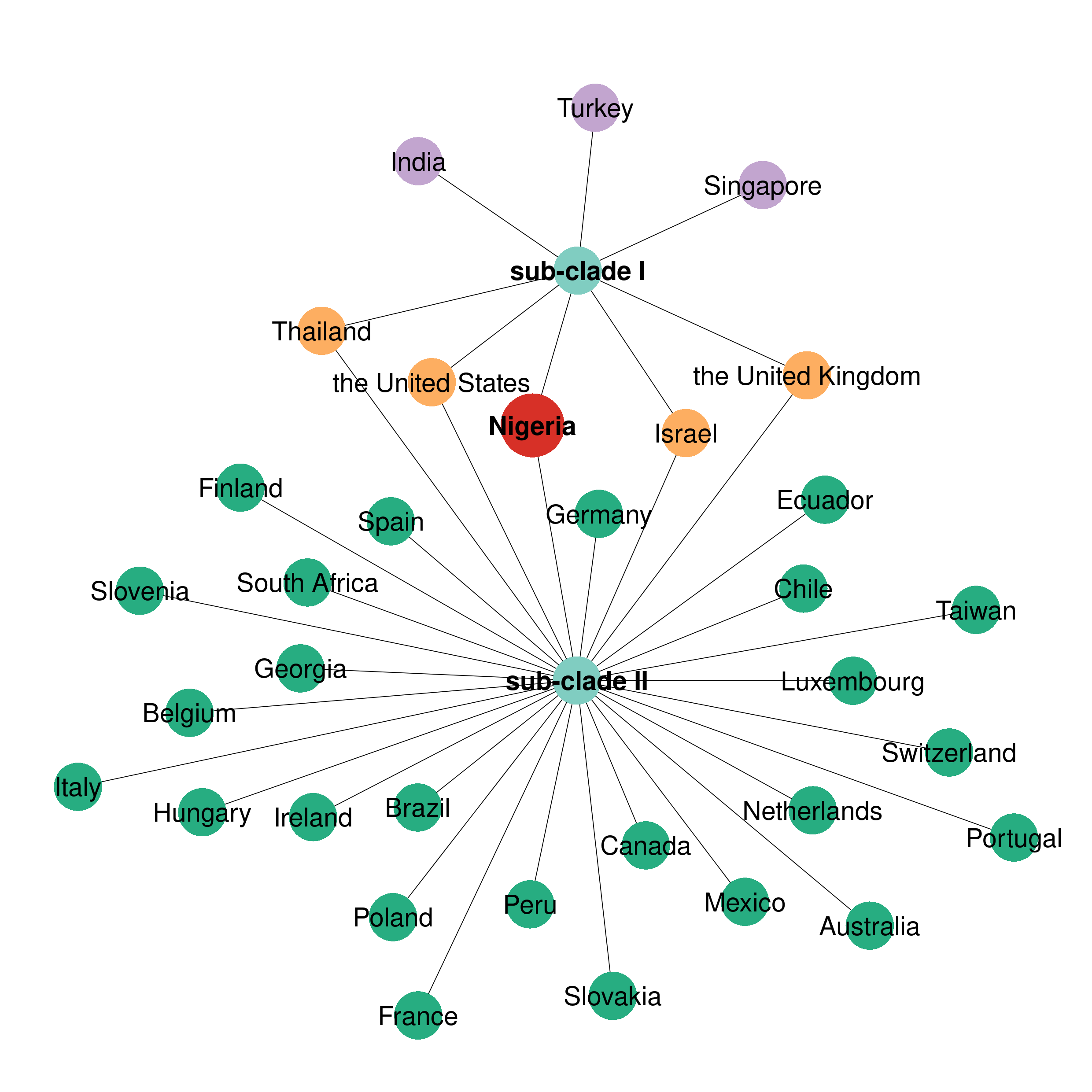

Supplement: Supplementary file 4 — Supplementary Fig.2 [file 41392_2023_1540_MOESM4_ESM.tif]
