# Supplementary material for: Monkeypox virus 2022, gene heterogeneity and protein polymorphism
Source: Signal Transduct Target Ther. 2023 Jul 17;8:278. doi: 10.1038/s41392-023-01540-2 (PMC10352349; doi:10.1038/s41392-023-01540-2)
Supplement: Supplementary file 5 — Supplementary_Materials_8463R1 [file 41392_2023_1540_MOESM5_ESM.docx]

Supplementary Materials for

Monkeypox virus 2022, gene heterogeneity and protein polymorphism

Yanjiao Li, Jingjing Hou, Zhong Sun, Jingjing Hu, Karuppiah Thilakavathy, Yuxi Wang, Zhongjun Shao, Yihan Lu, Weibing Wang, Chenglong Xiong.

Correspondence to: [xiongchenglong@fudan.edu.cn](mailto:xiongchenglong@fudan.edu.cn), [wwb@fudan.edu.cn](mailto:wwb@fudan.edu.cn), [luyihan@fudan.edu.cn](mailto:luyihan@fudan.edu.cn)

**This PDF file includes:**

Materials and Methods

Figures. S1 to S2

Tables. S1 to S2

Captions for Data S1 to S7

**Other Supplementary Materials for this manuscript include the following:**

Data S1 to S7

Data S1. Twenty-five phylogenetic trees of the monkeypox viruses’ genes or sequences,

Data S2. Polymorphism of protein: D7L,

Data S3. Polymorphism of protein: O1L,

Data S4. Polymorphism of protein: B5R,

Data S5. Heterogeneity of gene: B14R,

Data S6. Polymorphism of protein: B14R,

Data S7. Polymorphism of protein: T4.

Materials and Methods

**Retrieval of complete monkeypox virus (MPXV) genomes**

As of August 7, 2022, complete MPXV genomes were downloaded from EpiPox^TM^ database of Global Initiative on Sharing All Infuenza Data (GISAID) (https://www.epicov.org/epi3/frontend#4ffd75) and the MPXV Nucleotide Records of NCBI (https://www.ncbi.nlm.nih.gov/nuccore/?term=monkeypox+virus). Tick the complete option in GISAID and download the MPXVs’ genomes. In NCBI, download the sequences with the “complete” tag in annotation.

**Determination of target genes of MPXVs**

MPXV has a large double-stranded DNA genome, comprising ≈190 genes. Through literature review^5, 7-15^, 26 genes related to virulence, pathogenicity, host range, and those encoding basic structural proteins were included in this study. Due to the presence of spacer that is prone to variation between every two genes, the repeat regions at the left and right ends of MPXV’s genome are of great significance for analyzing its origin and evolution. The sequence within 190788-196450nt (according to the Refseq NC_063383), which is part of the right repeat region (Repeat region 2) and includes some of the above-mentioned genes, was also included in this study.

**Genes or sequences extraction**

According to the notes (https://www.ncbi.nlm.nih.gov/nuccore), most of the analyzed genes or sequences in the study were cut out by referring to the West African clade RefSeq (NC_063383), while the genes B14R and D14L were cut out by referring to the Congo Basin clade RefSeq (NC_003310), because these two were supposed to belong to the Congo Basin clade. These genes or sequences were used as references for alignment and extraction the correspondings from other MPXV genomes.

By using a combination of the iterative refinement method, the WSP and consistency scores and the algorithm of global alignment (Needleman-Wunsch) of the MAFFT software (v7.490 for Ubuntu 21.04)^16^, each genome of MPXV was compared/aligned with the references one by one. Besides, the extensional mafft.pl program (based on perl V5.32.1) developed by our team was used to extract the corresponding genes or sequences in each genome of MPXV from the alignments.

However, two strategies were used for gene or sequence extraction in this study. For the phylogenetic analyses, the redundant genomes were filtered out in advance by using CD-HIT^17^ with requiring identity > 95.0%, and then the unique one in each cluster was retained for the extraction of genes or sequences. It should be noted that some clusters have only one genome. When it comes to the polymorphism analyses of proteins encoded, the target genes in all genomes of MXPVs 2022 were extracted.

**Sequence alignments and phylogenetic analyses**

Multiple gene or sequence alignments were also performed by MAFFT v7.490. Phylogenetic analyses were carried out by using the Neighbor-joining and Maximum-likelihood methods in MEGA 6.06 ([https://www.megasoftware.net](https://www.megasoftware.net/)). Twelve strains including 8 collected earlier than the current epidemic of 2022 and 4 Refseqs specified in the databases of GISAID and NCBI were served as references.

**Polymorphism (in length) analyses for proteins encoded**

Multiple sequence alignment for each encoding gene was performed by using the Clustal W (codons) method in MEGA 6.06, and then translated the aligned nucleotide sequences into amino acids (AA). Record and describe the AA sequence length polymorphisms by observing the asterisk (*, symbol of terminator) manually. In order to ensure the research credibility, all protein variants with polymorphism in length were required to meet both i) no less than 3 MPXV strains have the same length in this protein, and ii) the genomes of these strains were submitted by at least 3 institutions to the database GISAID or NCBI. Exceptions, if any, will be specified in the main text.

The schematic diagrams of AA sequence were plotted by the Protean module of lasergene software package (version 7, <https://www.dnastar.com>) and only the domains of α-helix (Gamier-Robson method) were shown.

**Homology modeling and receptor-ligand docking**

The proteins encoded by B14R and T4 genes of MPXVs 2022 are long enough, and T4 protein has the same length as that of the Congo Basin clade strain. Although B14R gene also has a long enough protein product, which can reach 180 AA, it is still far from reaching the standard length of 326 AA of the Congo Basin clade strain. In order to find whether the 180 AA protein encoded by B14R of the West African clade MPXVs 2022 are functional, homology modeling and receptor-ligand docking were utilized to observe the similarities and differences of the main molecular forces between them and ligands.

Homology modeling was implemented by the network server of SWISS-MODEL (https://swissmodel.expasy.org/interactive)^18,19^. Interleukin-1 receptor type 2 (PDB 3o4o.1.B) was selected as the preferred template for both 180 AA and 326 AA products encoded by B14R for reasons of the highest amino acid sequence similarity (more than 54% of the coverage). Interleukin-1β (PDB 3o4o.1.A) was extracted as the ligand for the receptors (B14R proteins). ZDOCK-SERVER (https://zdock.umassmed.edu) was utilized for receptor-ligand docking^20^.

The PyMOL (version 2.1.0, https://pymol.org/2) program were used to read, annotate and plot the spatial structure of protein and intermolecular hydrogen bonds.

**Additional references**

1. Karumathil, S. *et al.* Evolution of Synonymous Codon Usage Bias in West African and Central African Strains of Monkeypox Virus. *Evolutionary Bioinformatics* **14**, 117693431876136 (2018).
2. Likos, A. M. *et al.* A tale of two clades: monkeypox viruses. *J Gen Virol*. **86**, 2661-2672 (2005).
3. Weaver, J. R. & Isaacs, S. N. Monkeypox virus and insights into its immunomodulatory proteins. *Immunol Rev*. **225**, 96-113 (2008).
4. Senkevich, T. G., Yutin, N., Wolf, Y. I., Koonin, E. V. & Moss, B. Ancient Gene Capture and Recent Gene Loss Shape the Evolution of Orthopoxvirus-Host Interaction Genes. *mBio* **12**, e0149521 (2021).
5. Odom, M. R., Hendrickson, R. C. & Lefkowitz, E. J. Poxvirus protein evolution: family wide assessment of possible horizontal gene transfer events. *Virus Res*. **144**, 233-249 (2009).
6. Xu, Z. *et al*. Identification of 10 cowpox virus proteins that are necessary for induction of hemorrhagic lesions (red pocks) on chorioallantoic membranes. *J Virol*. **88**, 8615-8628 (2014).
7. Xu, Z., Zikos, D., Osterrieder, N. & Tischer, B. K. Generation of a complete single-gene knockout bacterial artificial chromosome library of cowpox virus and identification of its essential genes. *J Virol*. **88**, 490-502 (2014).
8. Afonso, P. P. *et al*. Biological characterization and next-generation genome sequencing of the unclassified Cotia virus SPAn232 (Poxviridae). *J Virol*. **86**, 5039-5054 (2012).
9. Morikawa, S. *et al*. An attenuated LC16m8 smallpox vaccine: analysis of full-genome sequence and induction of immune protection. *J Virol*. **79**, 11873-11891 (2005).
10. Katoh, K., Misawa, K., Kuma, K. & Miyata, T. MAFFT: a novel method for rapid multiple sequence alignment based on fast Fourier transform. *Nucleic Acids Res*. **30**, 3059-3066 (2002).
11. Li, W. & Godzik, A. Cd-hit: a fast program for clustering and comparing large sets of protein or nucleotide sequences. *Bioinformatics* **22**, 1658-1659 (2006).
12. Waterhouse, A. *et al*. SWISS-MODEL: homology modelling of protein structures and complexes. *Nucleic Acids Res*. **46(W1)**, W296-W303 (2018).
13. Studer, G. *et al*. QMEANDisCo-distance constraints applied on model quality estimation. *Bioinformatics* **36**, 1765-1771 (2020). *Erratum*: **36**, 2647 (2020).
14. Pierce, B. G. *et al*. ZDOCK server: interactive docking prediction of protein-protein complexes and symmetric multimers. *Bioinformatics* **30**, 1771-1773 (2014).


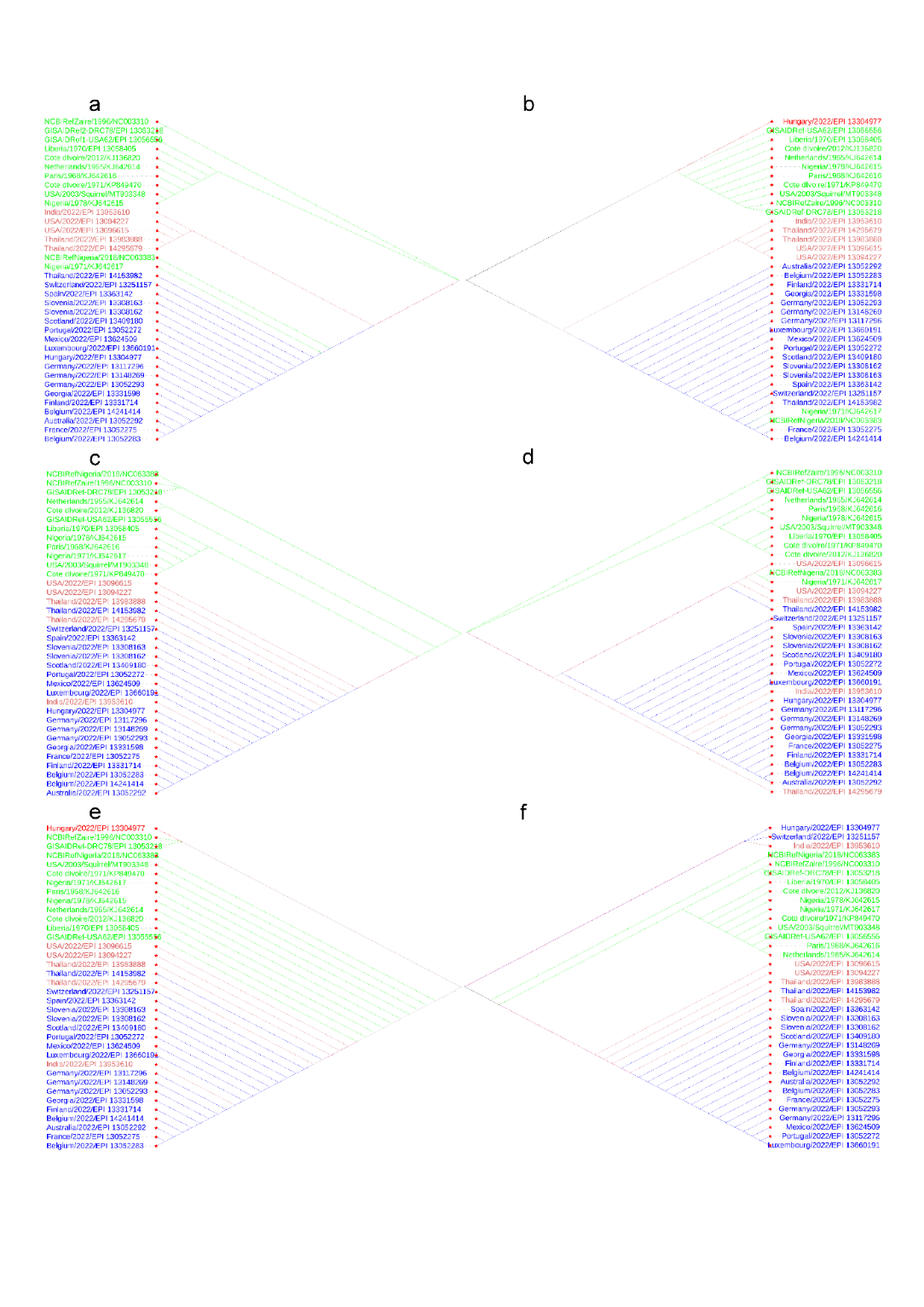


**Figure. S1. Phylogenetic tree types of genes/sequences within the genomes of monkeypox viruses, 2022**

a, two-branch type (using V-slfn gene as the example). b, two-branch-plus type (using B5R gene as the example). c, the standard one-branch type (E3L gene). d and e, two variants of the one-branch type. As shown in d (N1R gene), the cluster of hMpxV/Thailand/NIC-PKT-M1/2022 (EPI_14295679, belonging to sub-clade I) clearly demonstrates the distance from others, while in e (K4L gene), the cluster of hMpxV/Hungary/NBL-001/2022 (EPI_13304977, belonging to sub-clade II) is noticeably divergent from others. f, irregular type (using C1L gene as the example). Strains in green are references, in firebrick red are sub-clade I strains, in blue are sub-clade II strains, and in bright red are the distinctive strains of interest.

**
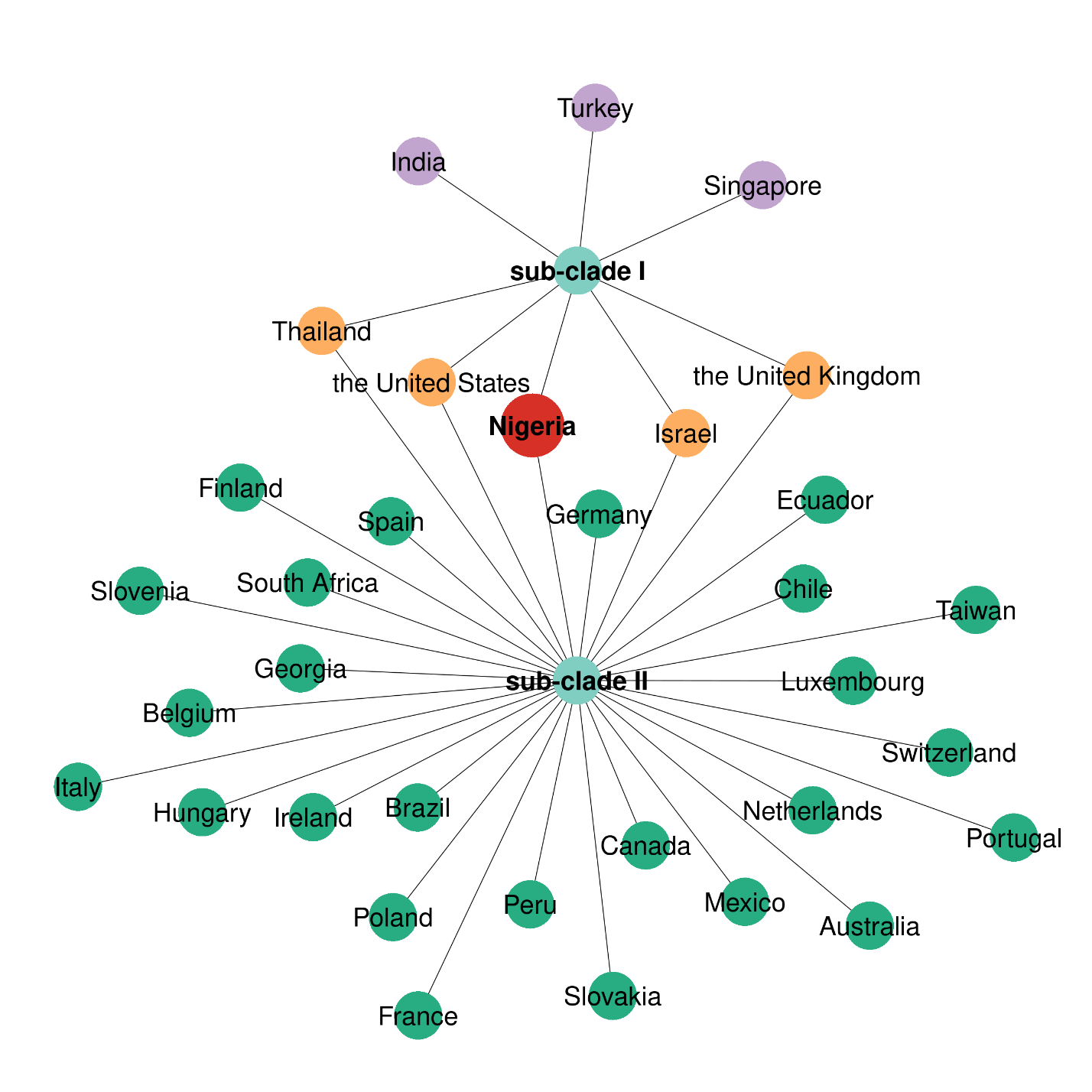
Figure. S2. Epidemic areas affected by the monkeypox viruses, 2022 (as of August 7, 2022)**

Epidemic areas were identified by analyzing the names of isolates belonging to sub-clades I and II in the evolutionary phylogenetic trees of the K1L, O1L_trctd, and A46R genes. The network plot shows that areas affected by strains in sub-clade I are relatively limited, mainly to the United States, the United Kingdom, Thailand, Israel, India, Turkey, and Singapore. In contrast, the epidemic areas affected by strains in sub-clade II are extensive. Strains in this sub-clade are primarily responsible for the current global outbreak. Nigeria is the common origin of viruses in both sub-clades I and II of the West African clade. Additionally, the United States, the United Kingdom, Thailand, and Israel have reported imported cases infected by isolates from both sub-clades I and II.

Table S1. Genes and sequences used in this study

Table S2. Unique clusters and their redundant genomes

Data S1. Twenty-five phylogenetic trees of the monkeypox viruses’ genes or sequences (PPTX)

Data S2. Polymorphism of protein: D7L (FAS)

Data S3. Polymorphism of protein: O1L (FAS)

Data S4. Polymorphism of protein: B5R (FAS)

Data S5. Heterogeneity of gene: B14R (FAS)

Data S6. Polymorphism of protein: B14R (FAS)

Data S7. Polymorphism of protein: T4 (FAS)
