# Supplementary material for: Monkeypox virus 2022, gene heterogeneity and protein polymorphism
Source: Signal Transduct Target Ther. 2023 Jul 17;8:278. doi: 10.1038/s41392-023-01540-2 (PMC10352349; doi:10.1038/s41392-023-01540-2)
Supplement: Supplementary file 6 — Data S1 [file 41392_2023_1540_MOESM6_ESM.pptx]

## Slide 1
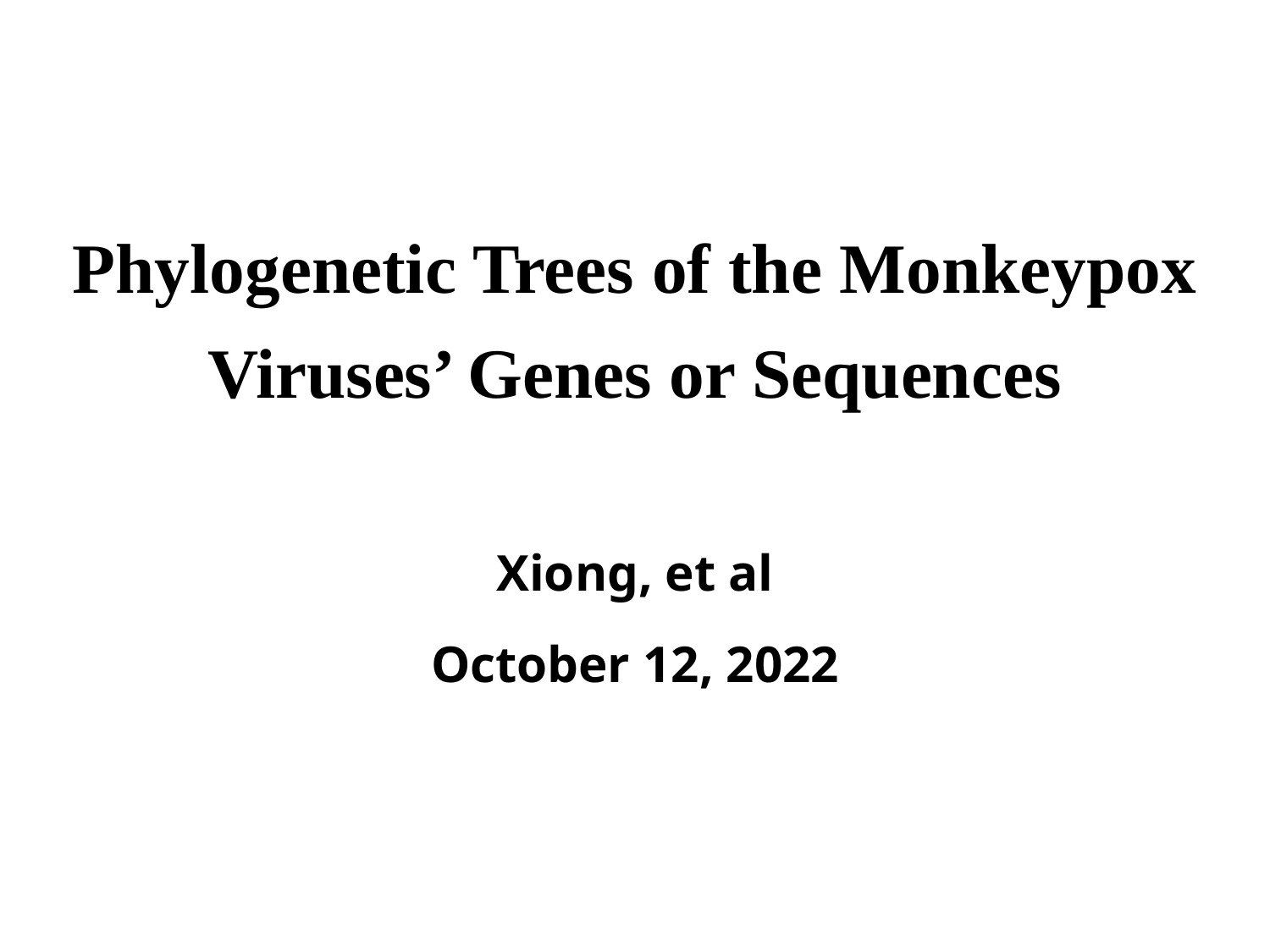

# Phylogenetic Trees of the Monkeypox Viruses’ Genes or Sequences
Xiong, et al
October 12, 2022

## Slide 2
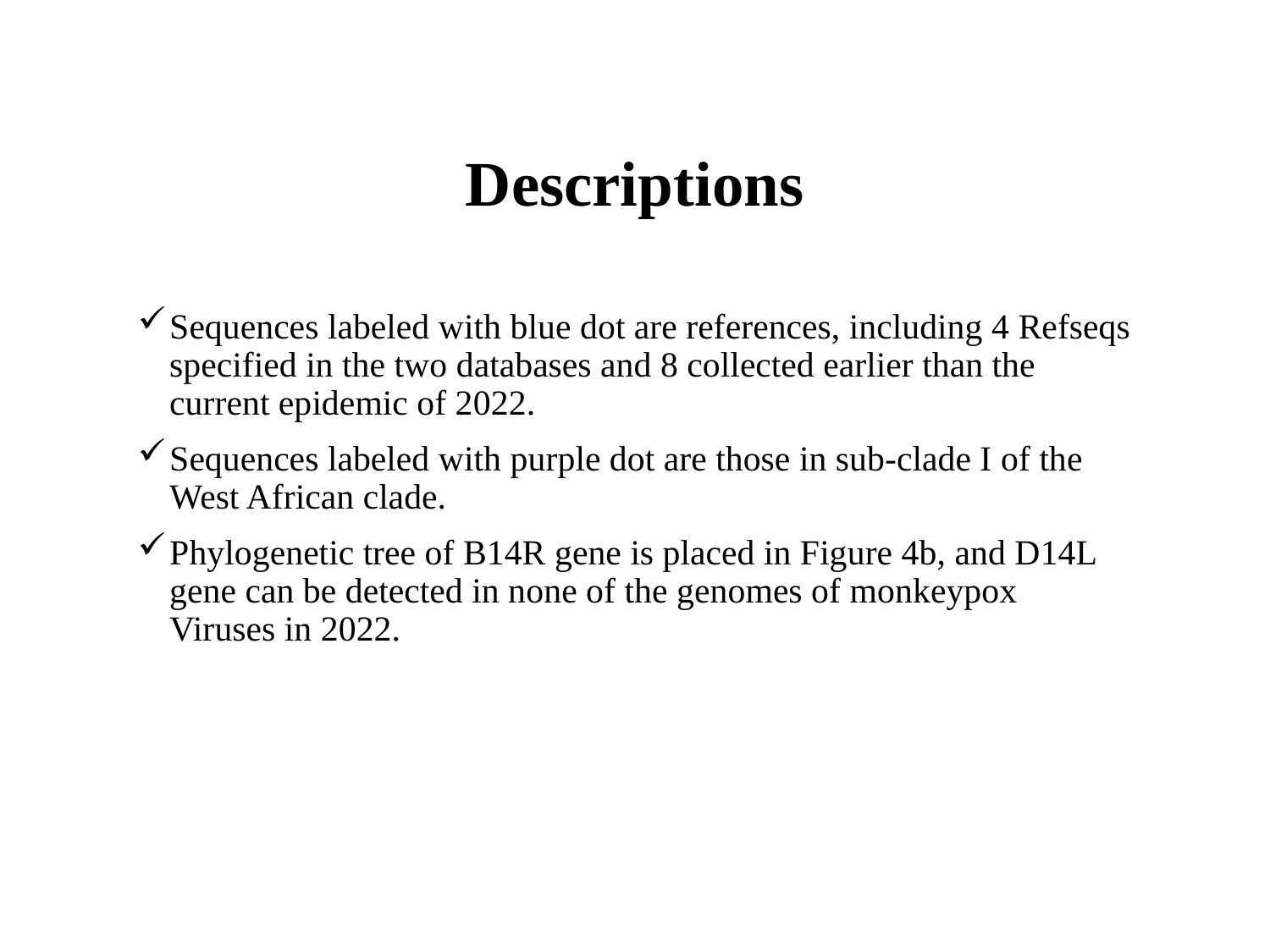

# Descriptions
Sequences labeled with blue dot are references, including 4 Refseqs specified in the two databases and 8 collected earlier than the current epidemic of 2022.
Sequences labeled with purple dot are those in sub-clade I of the West African clade.
Phylogenetic tree of B14R gene is placed in Figure 4b, and D14L gene can be detected in none of the genomes of monkeypox Viruses in 2022.

## Slide 3
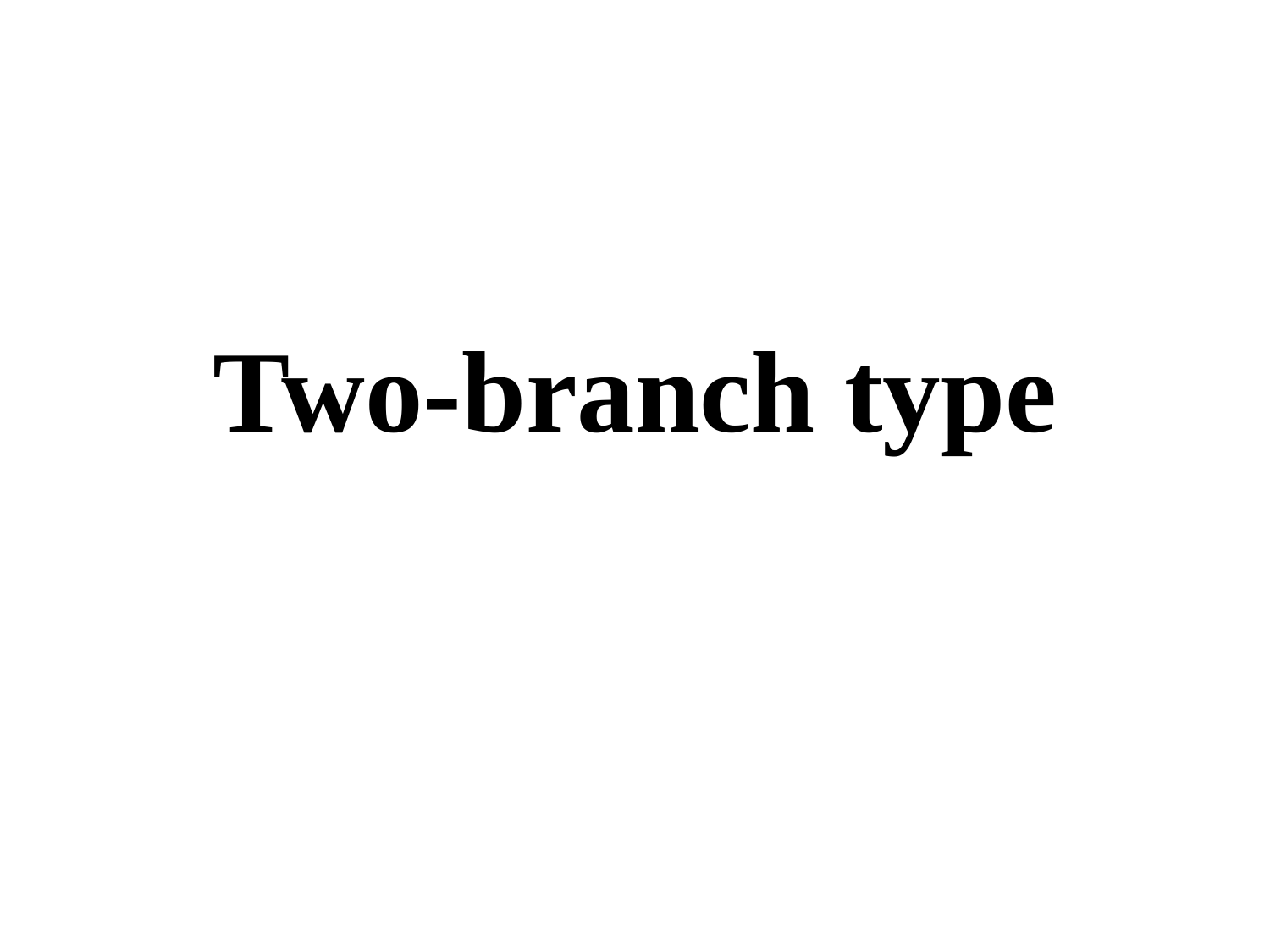

# Two-branch type

## Slide 4
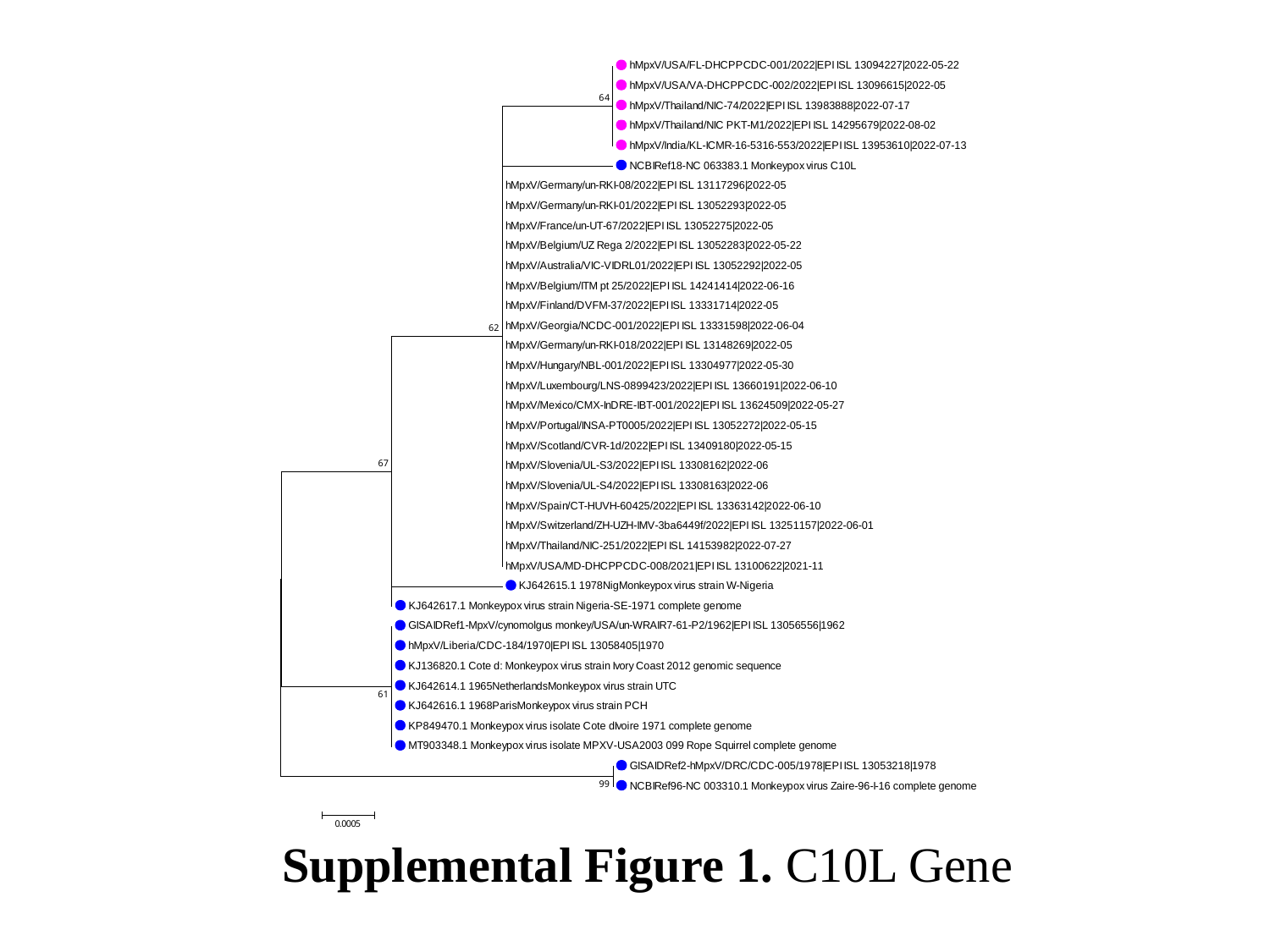

# Supplemental Figure 1. C10L Gene

## Slide 5
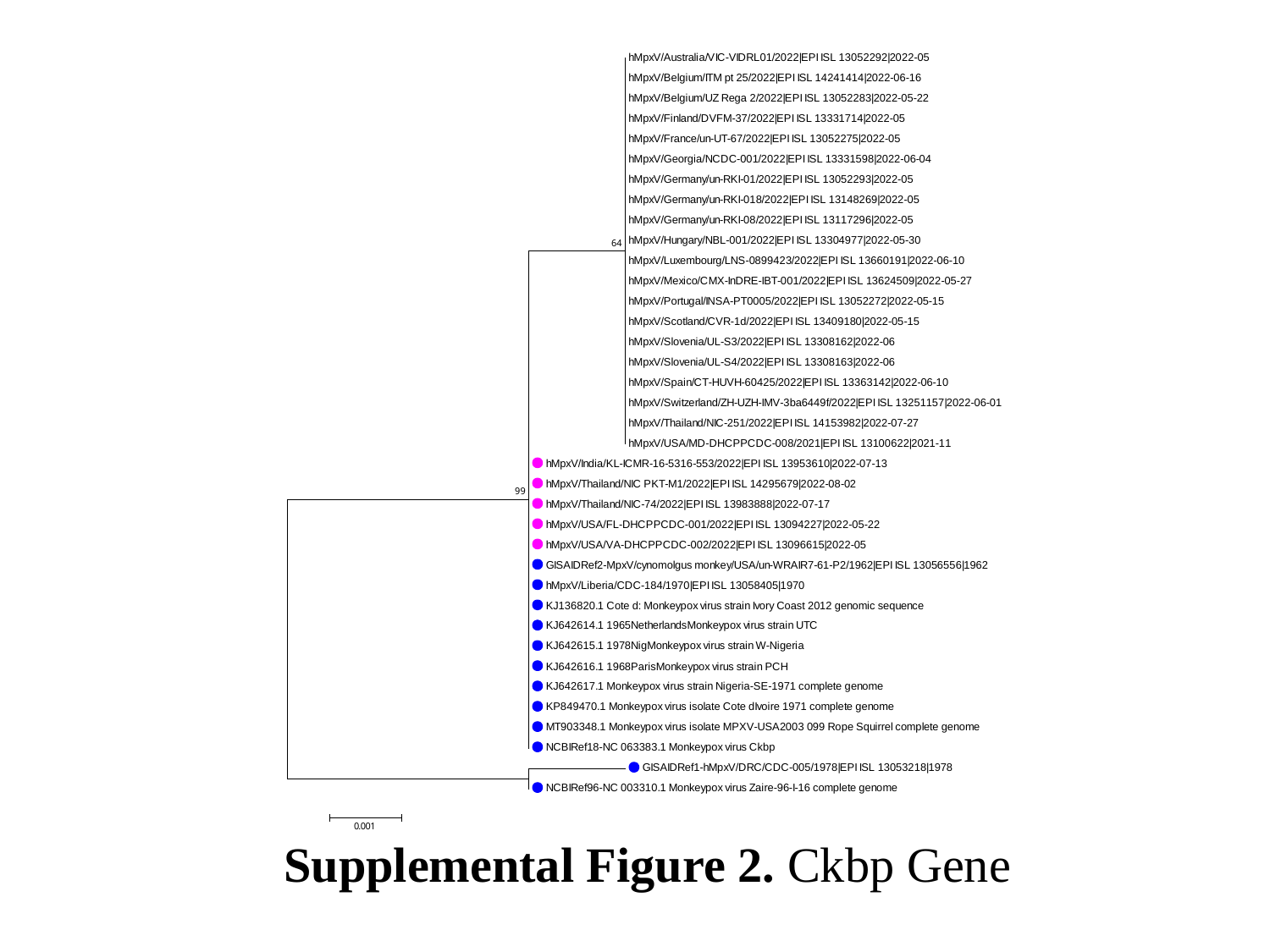

# Supplemental Figure 2. Ckbp Gene

## Slide 6
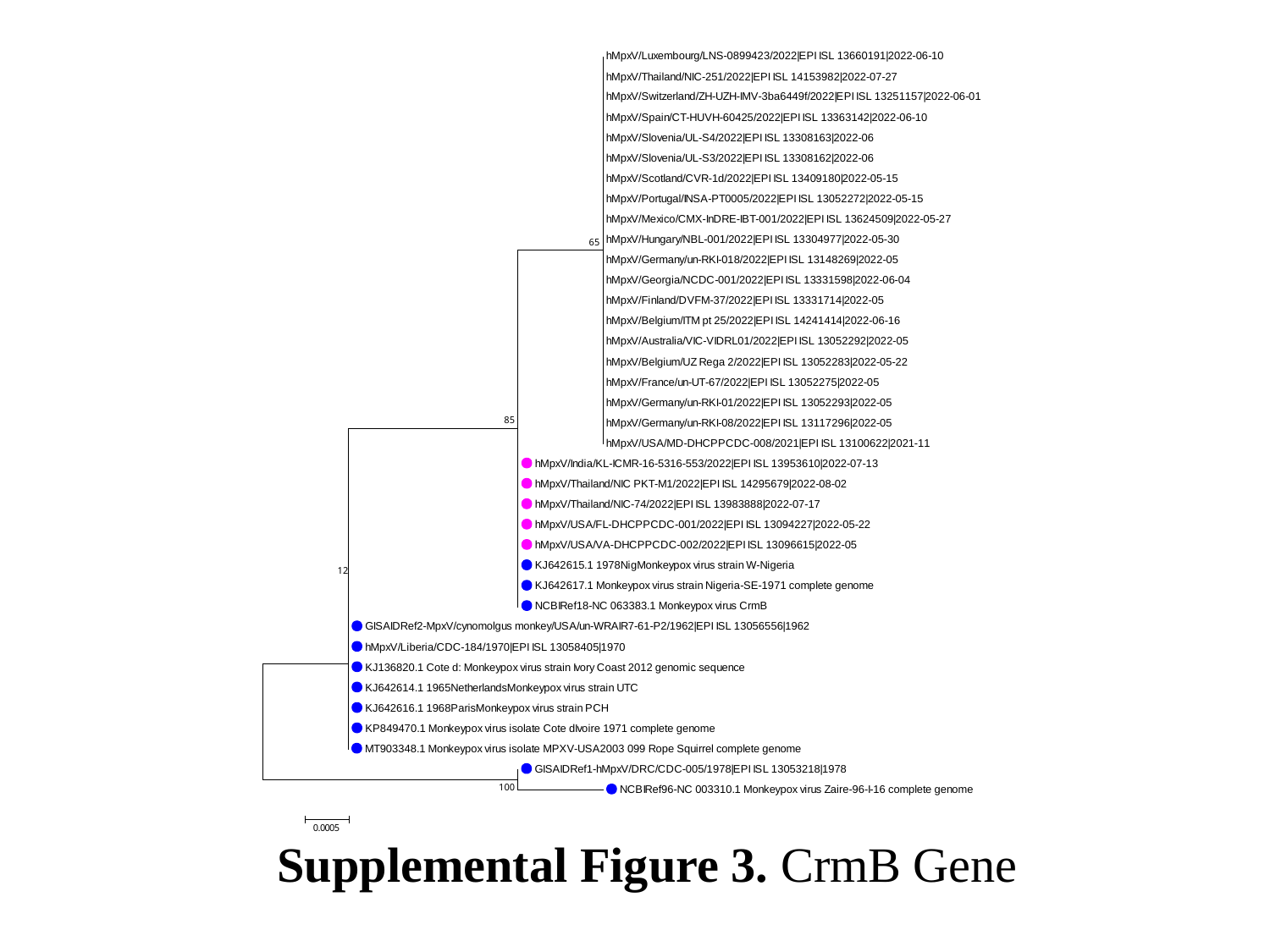

# Supplemental Figure 3. CrmB Gene

## Slide 7
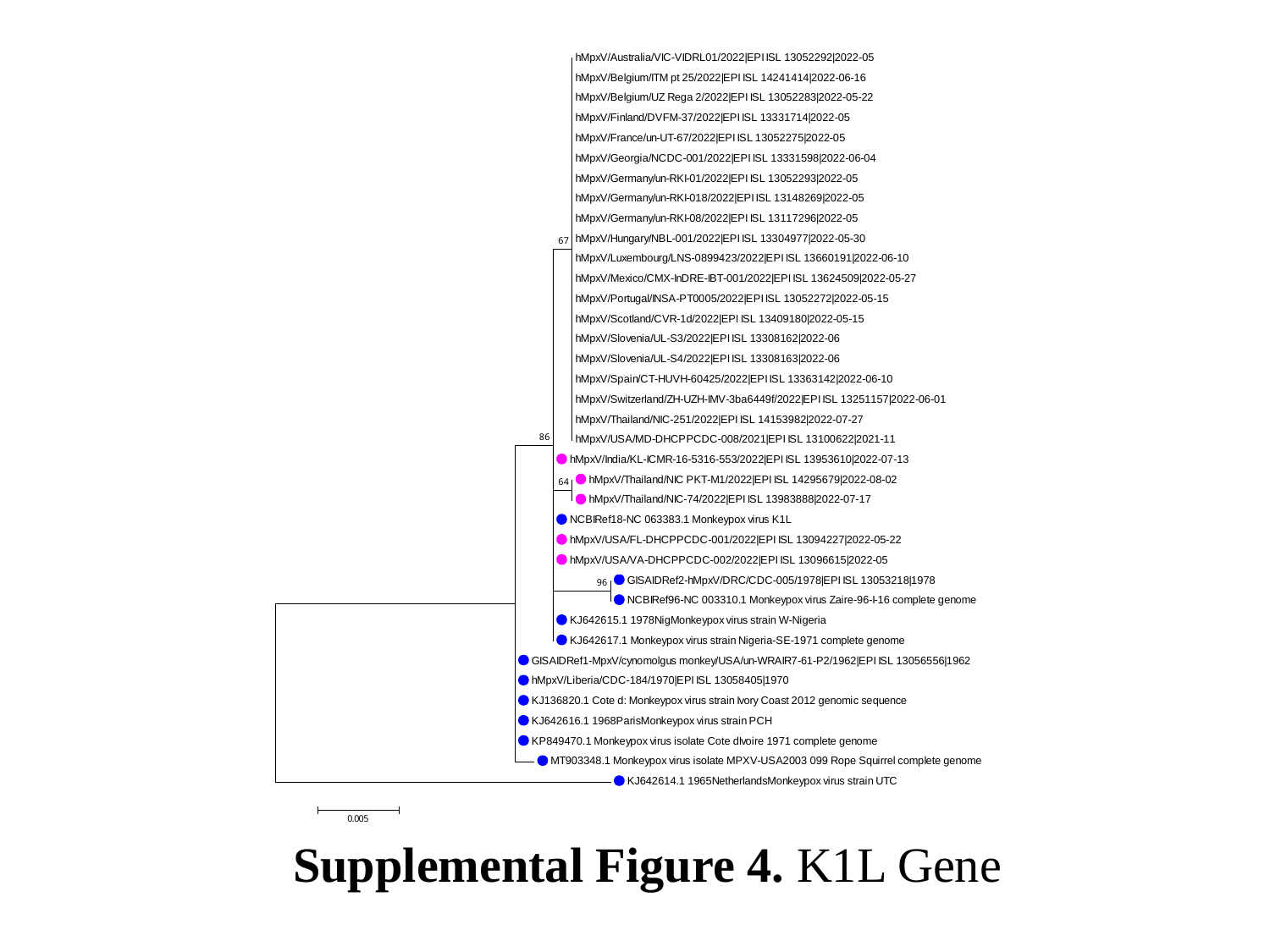

# Supplemental Figure 4. K1L Gene

## Slide 8
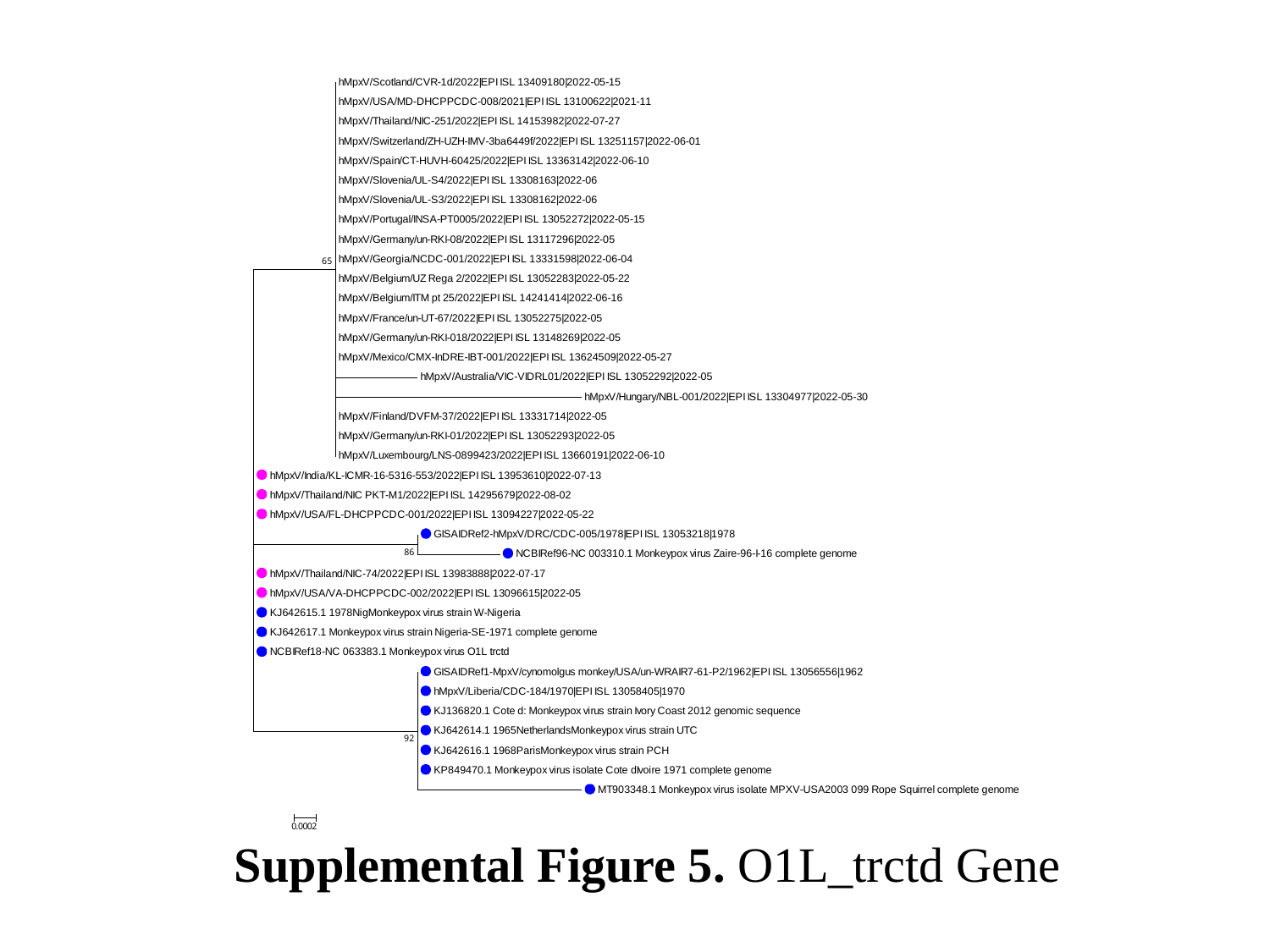

# Supplemental Figure 5. O1L_trctd Gene

## Slide 9
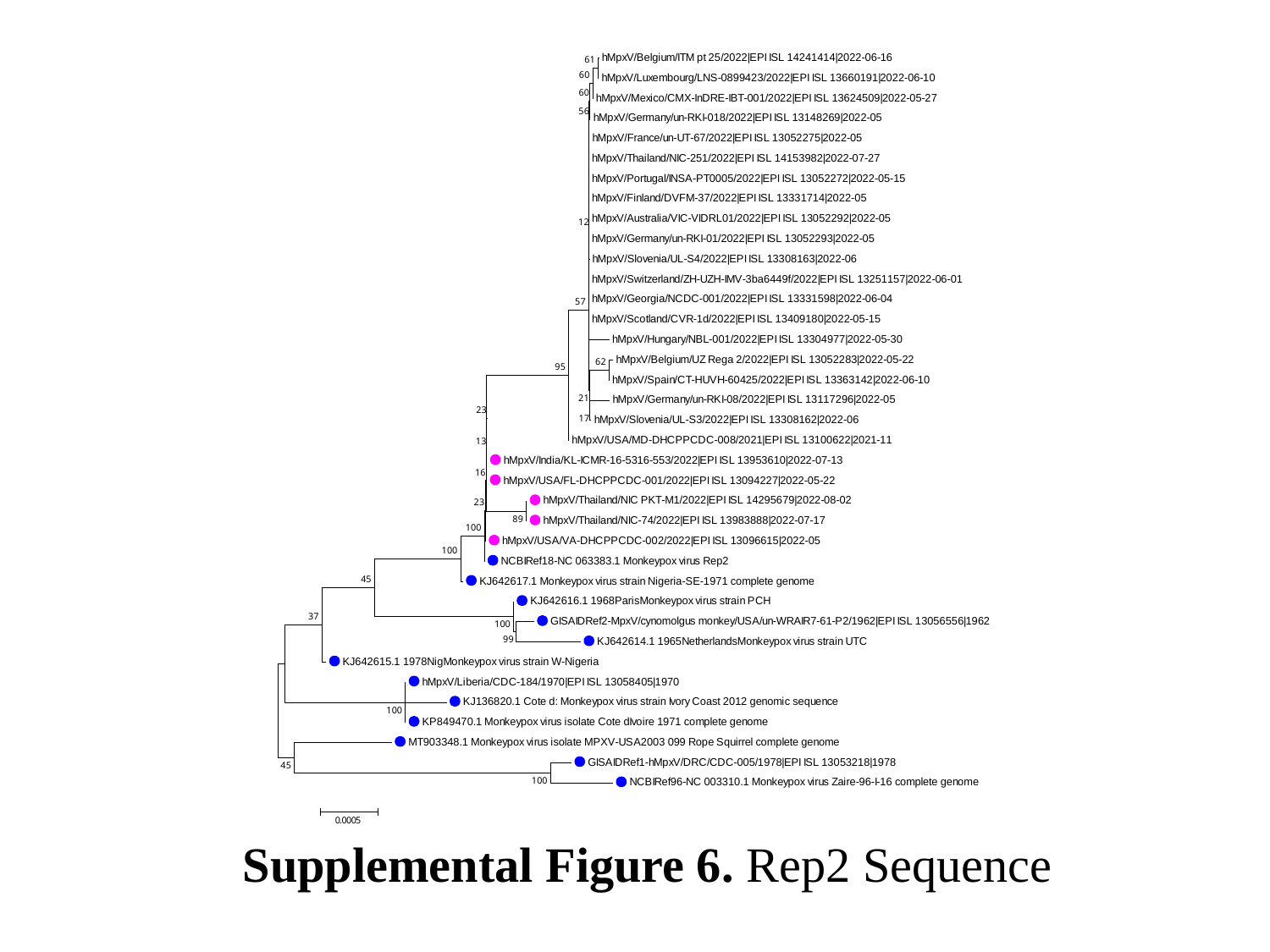

# Supplemental Figure 6. Rep2 Sequence

## Slide 10
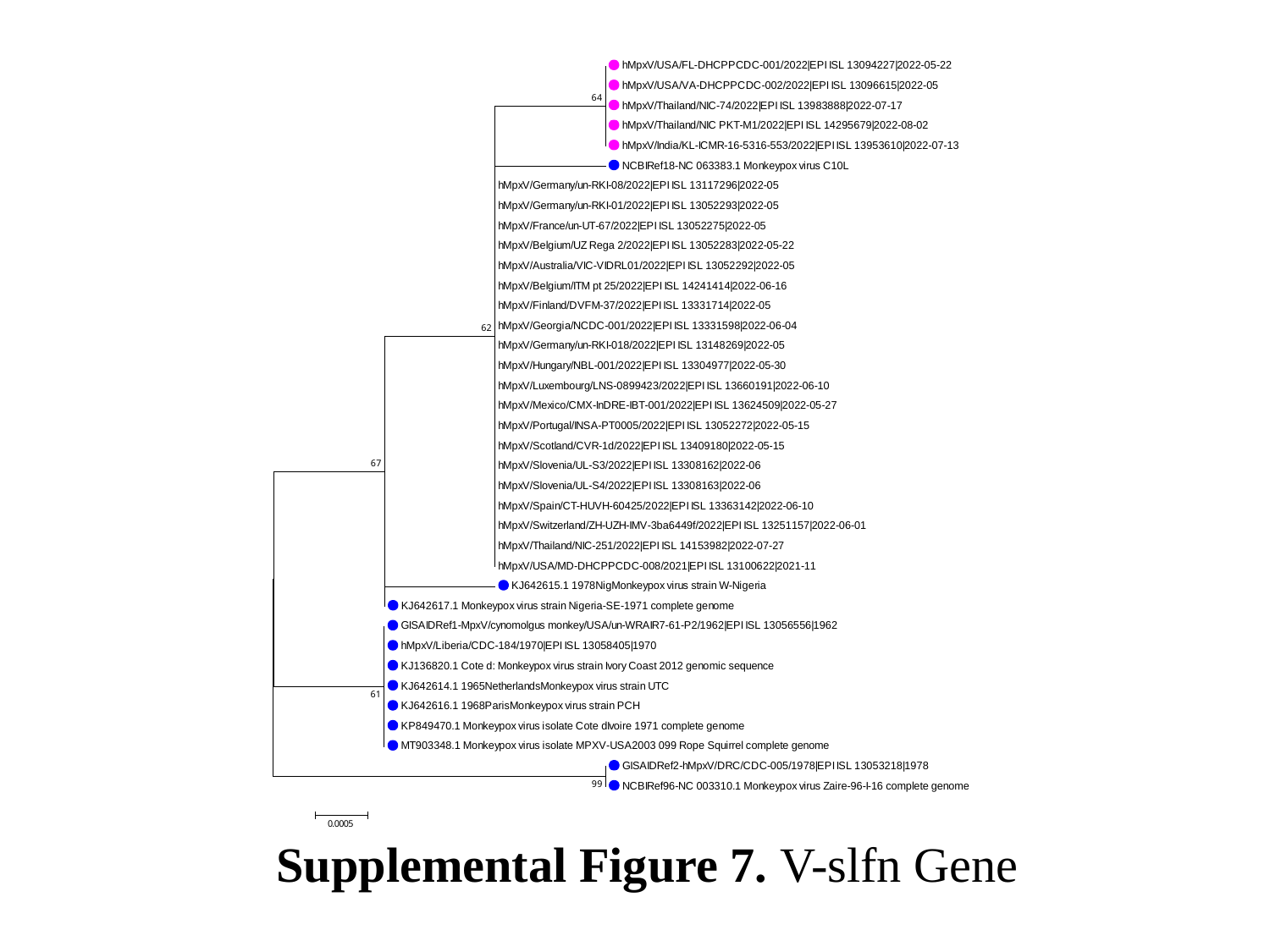

# Supplemental Figure 7. V-slfn Gene

## Slide 11
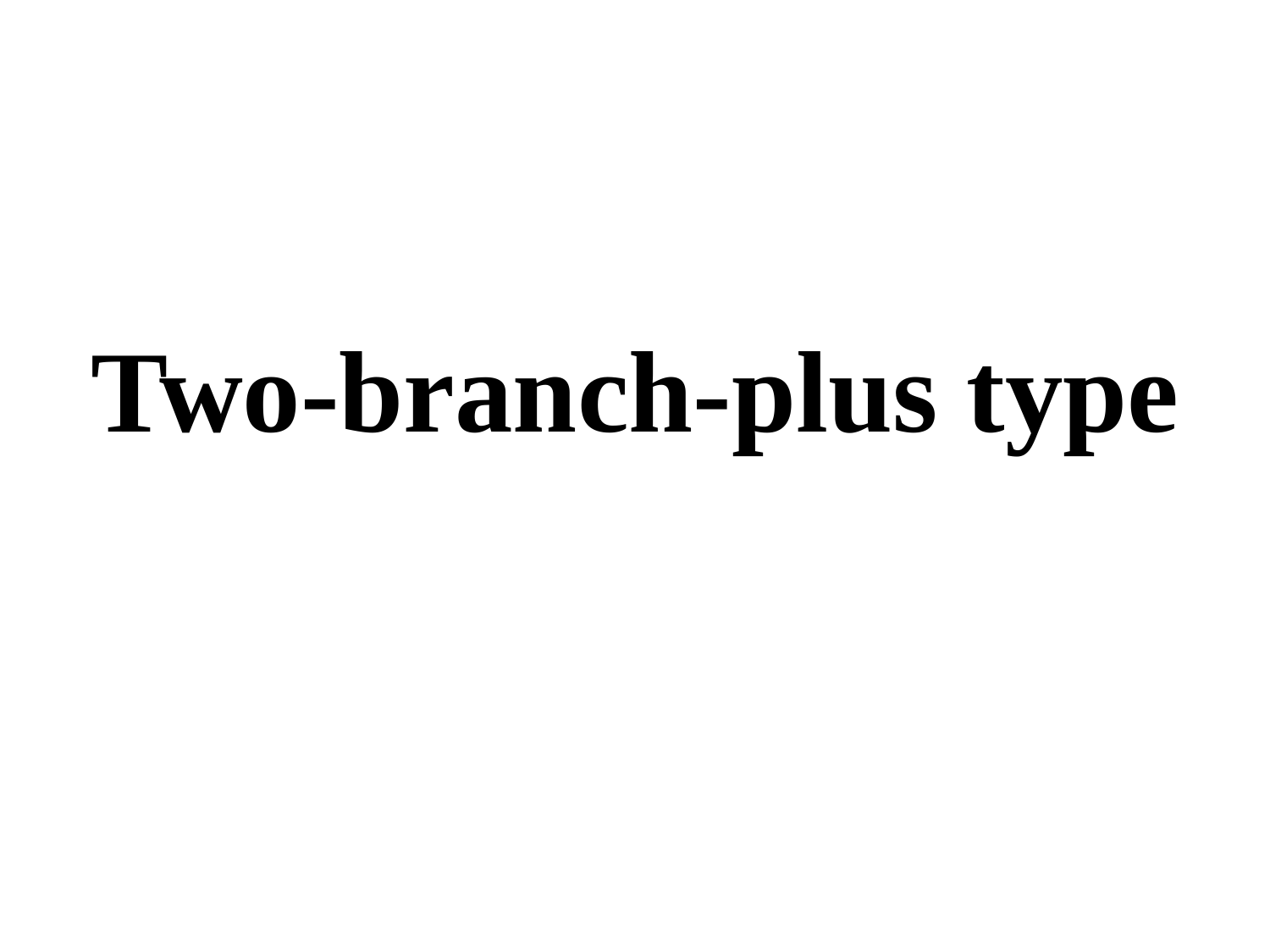

# Two-branch-plus type

## Slide 12
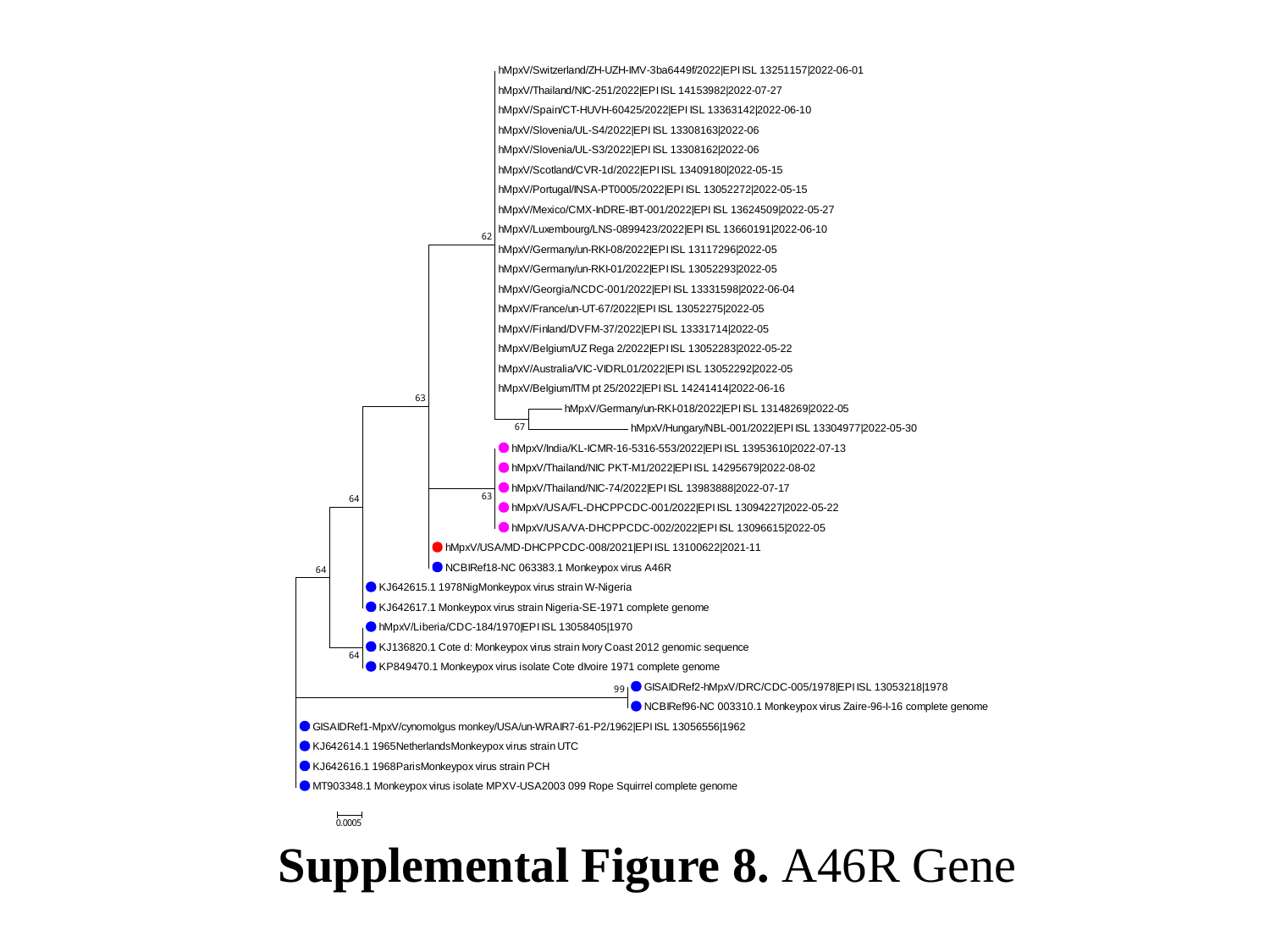

# Supplemental Figure 8. A46R Gene

## Slide 13
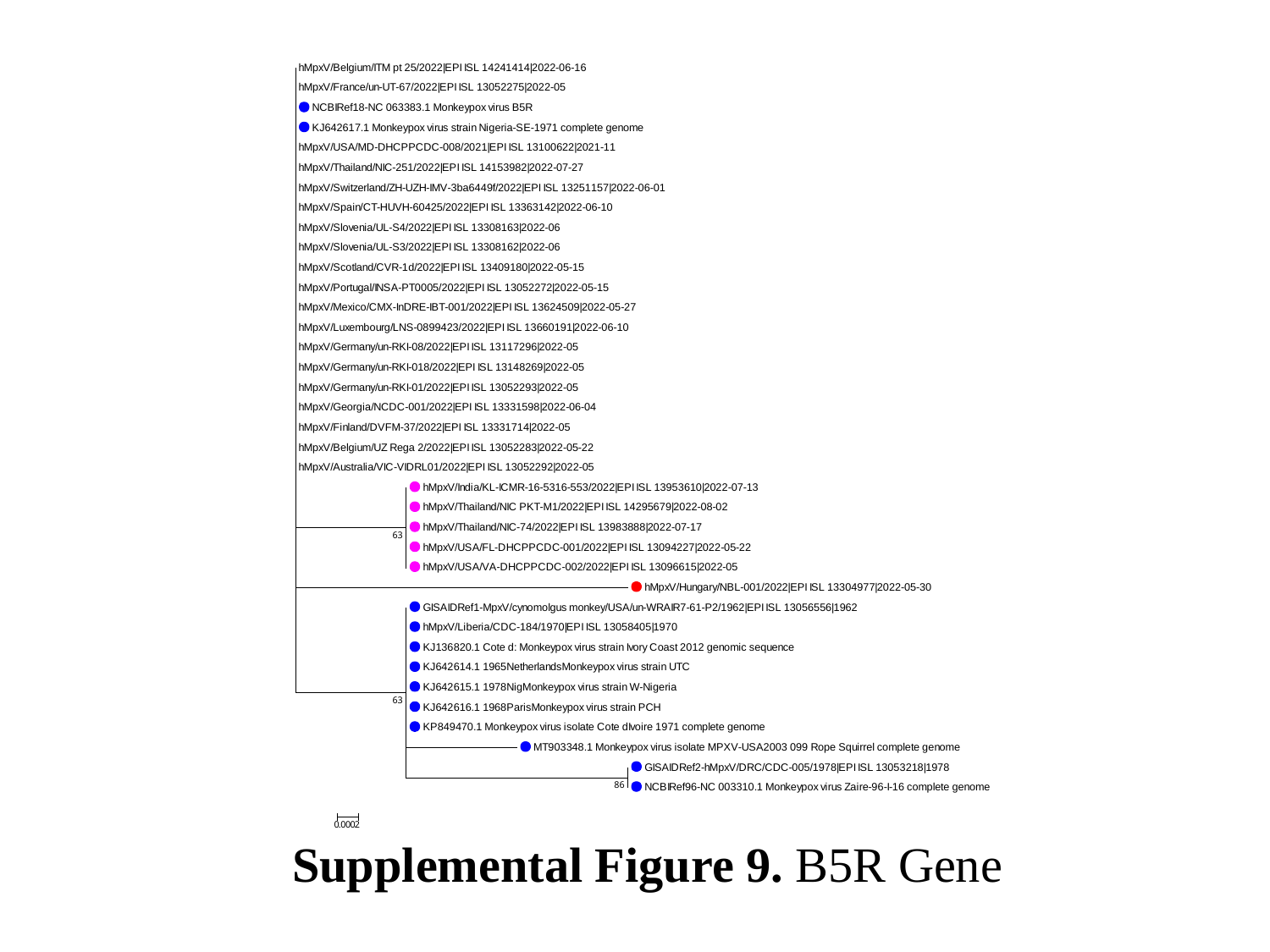

# Supplemental Figure 9. B5R Gene

## Slide 14
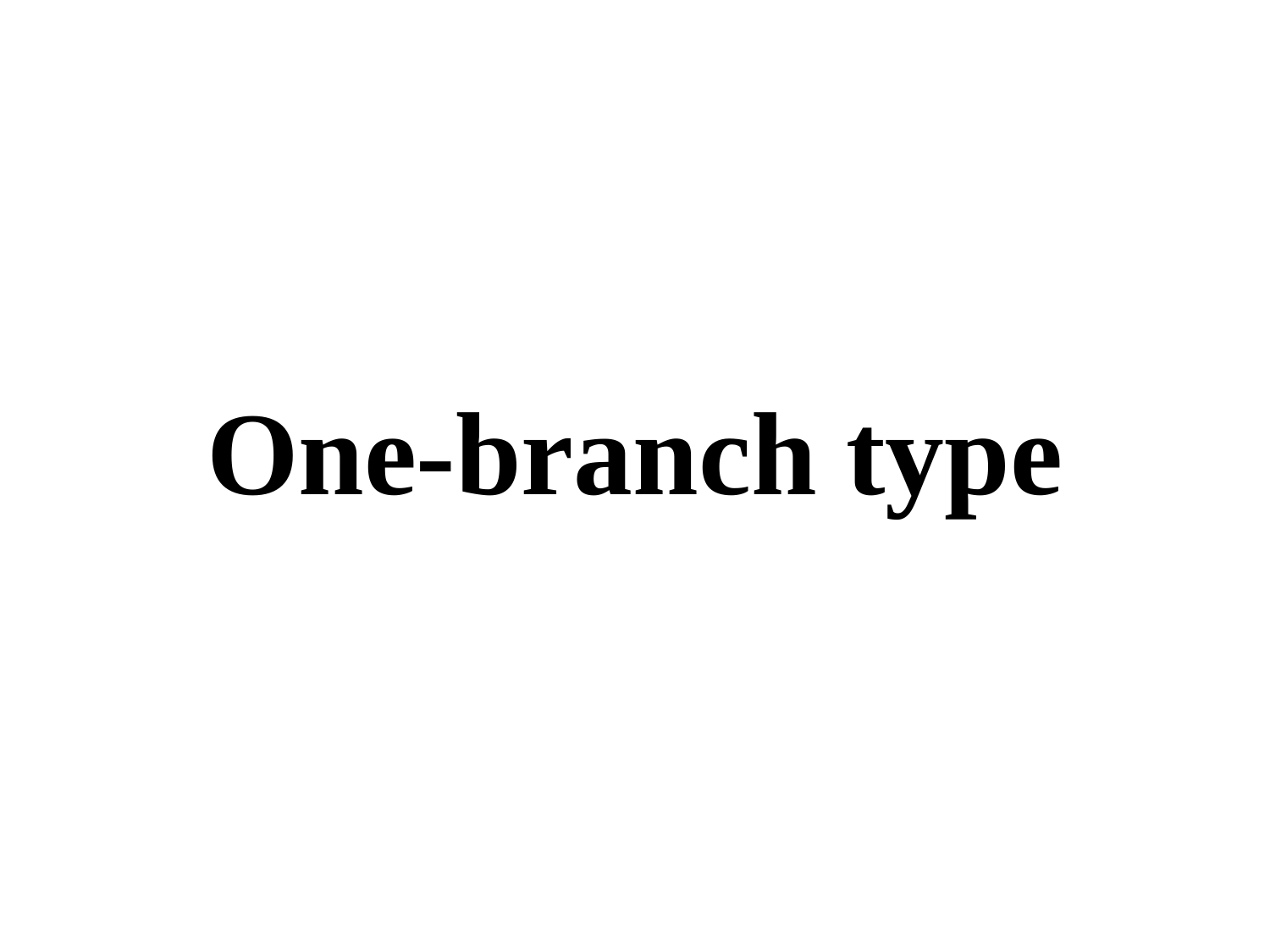

# One-branch type

## Slide 15
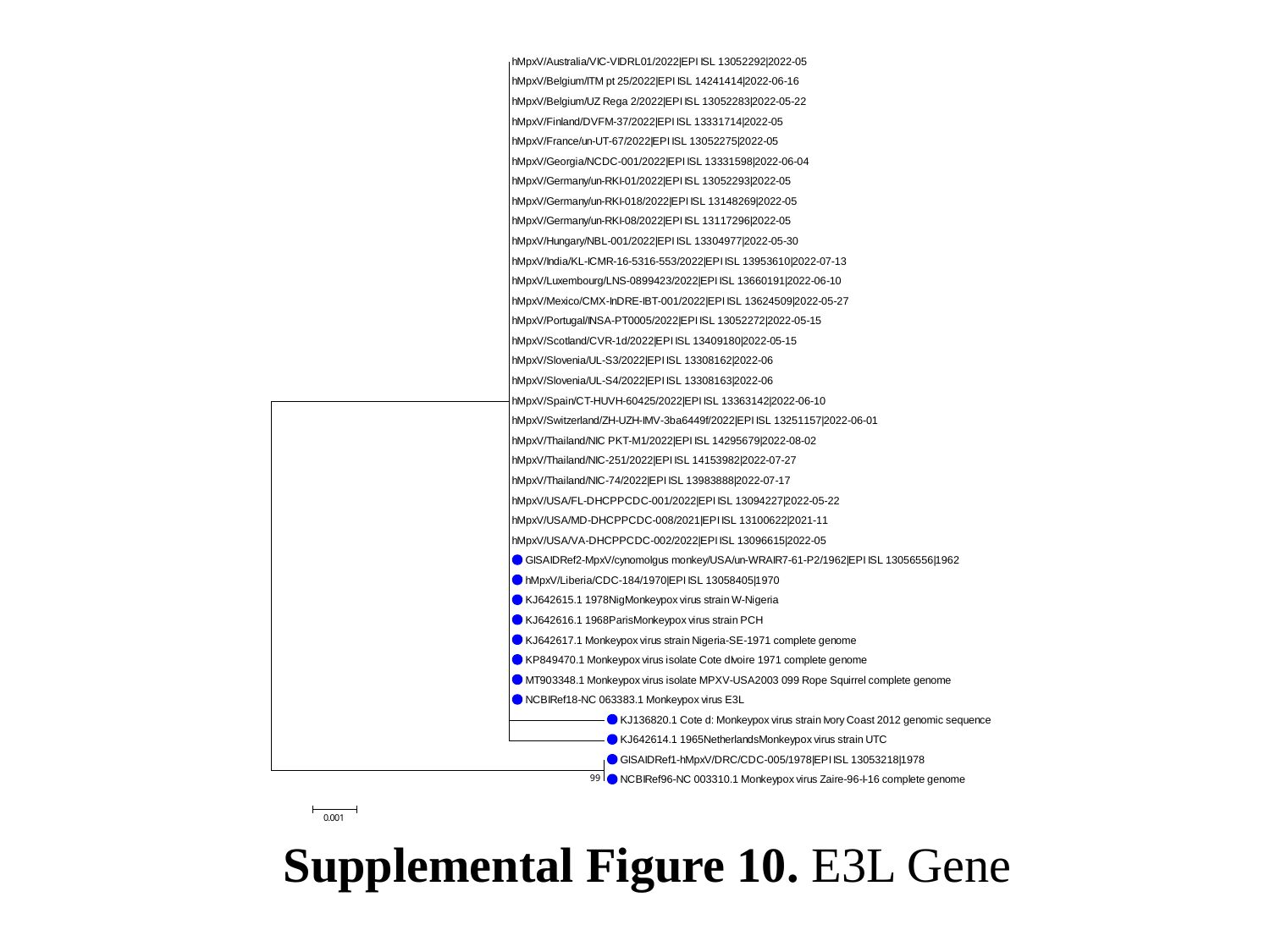

# Supplemental Figure 10. E3L Gene

## Slide 16
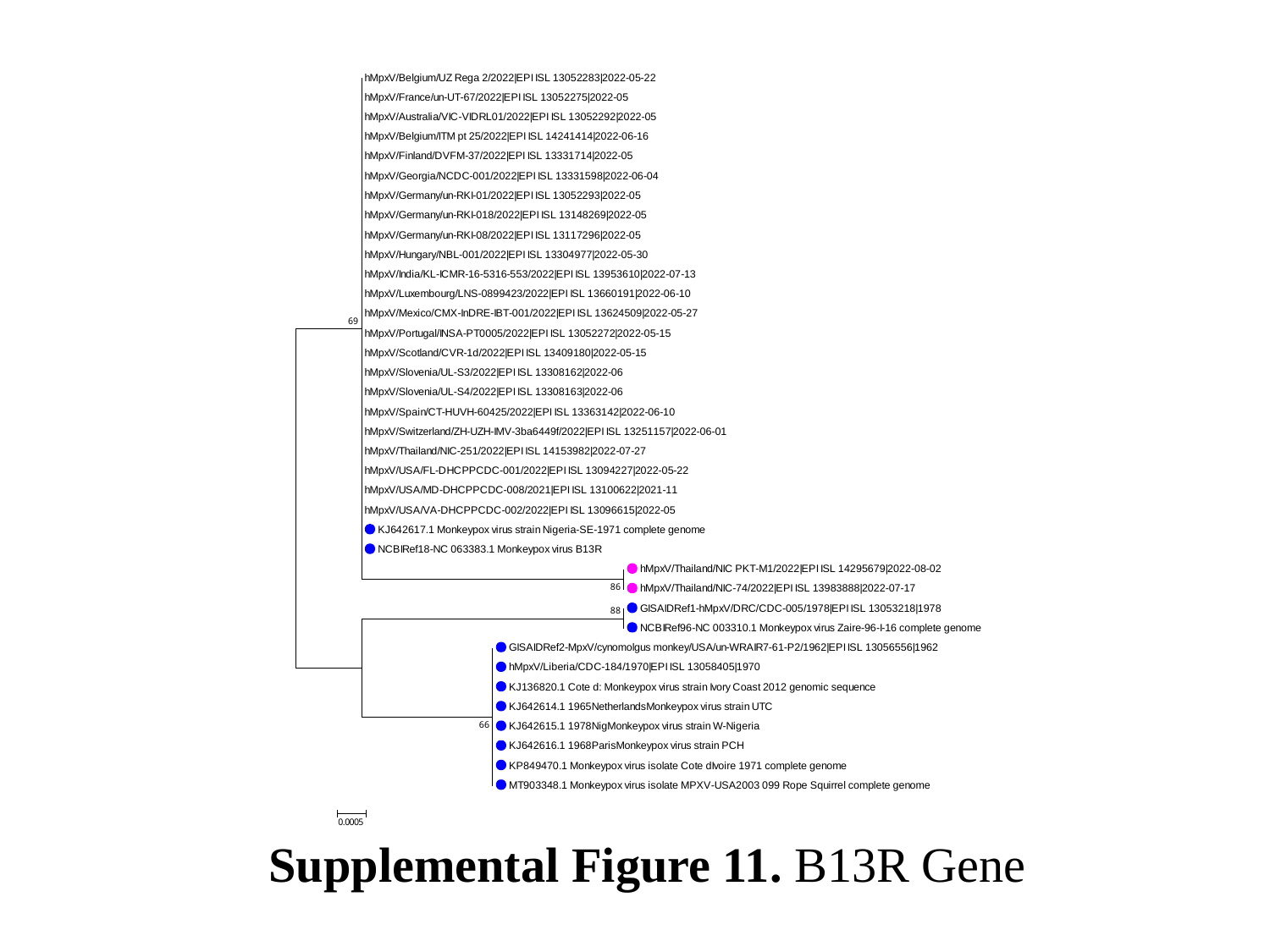

# Supplemental Figure 11. B13R Gene

## Slide 17
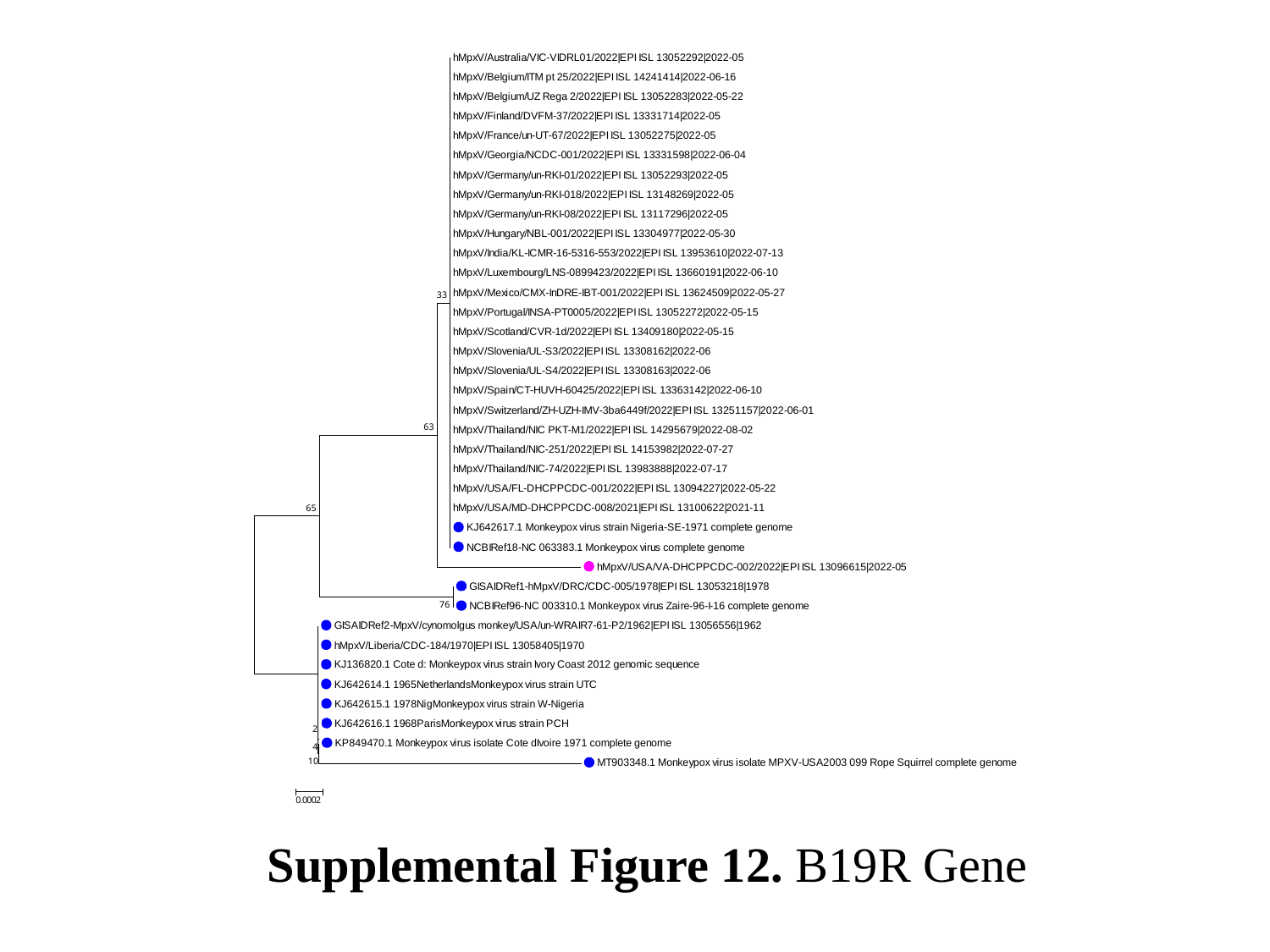

# Supplemental Figure 12. B19R Gene

## Slide 18
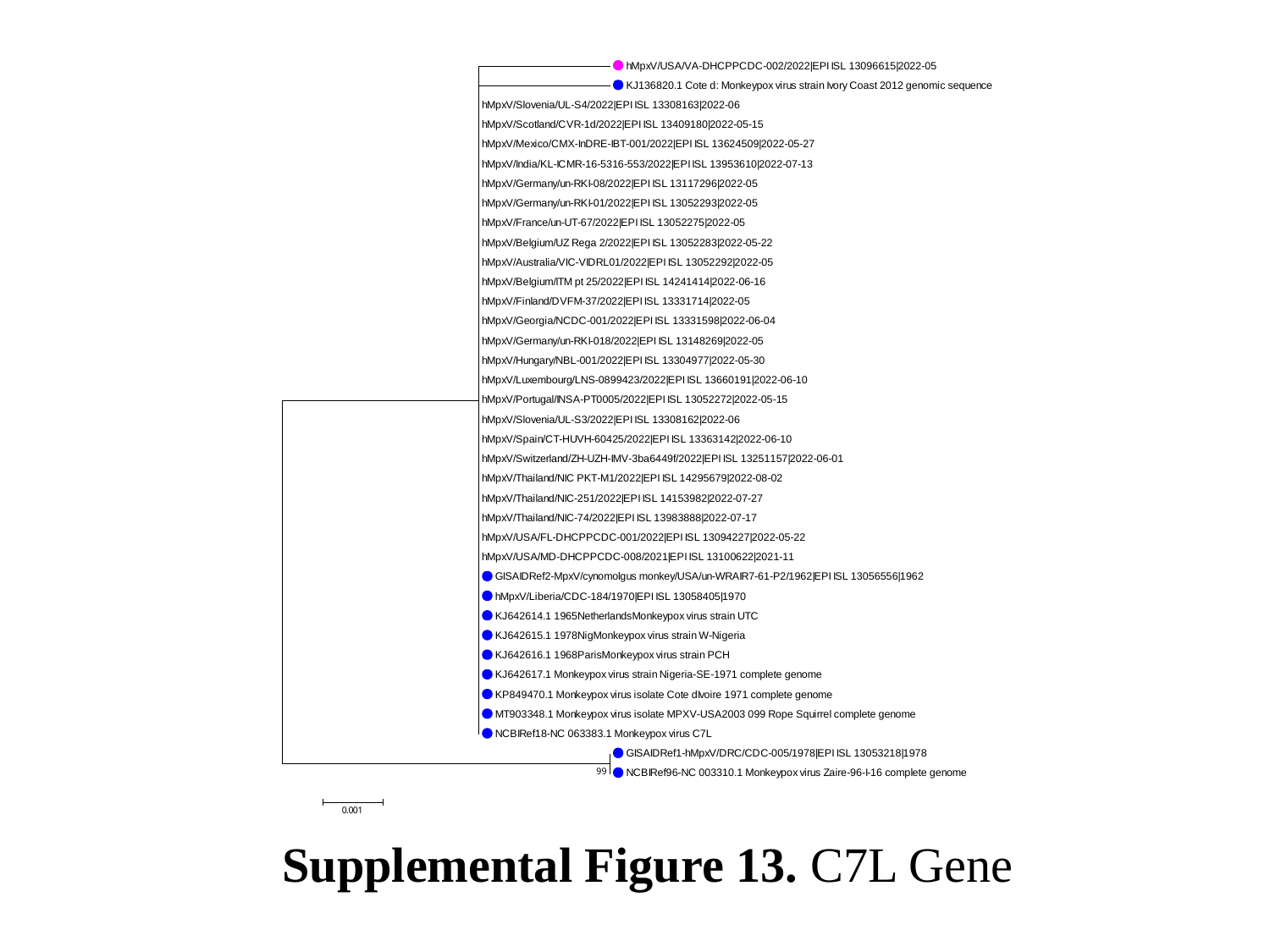

# Supplemental Figure 13. C7L Gene

## Slide 19
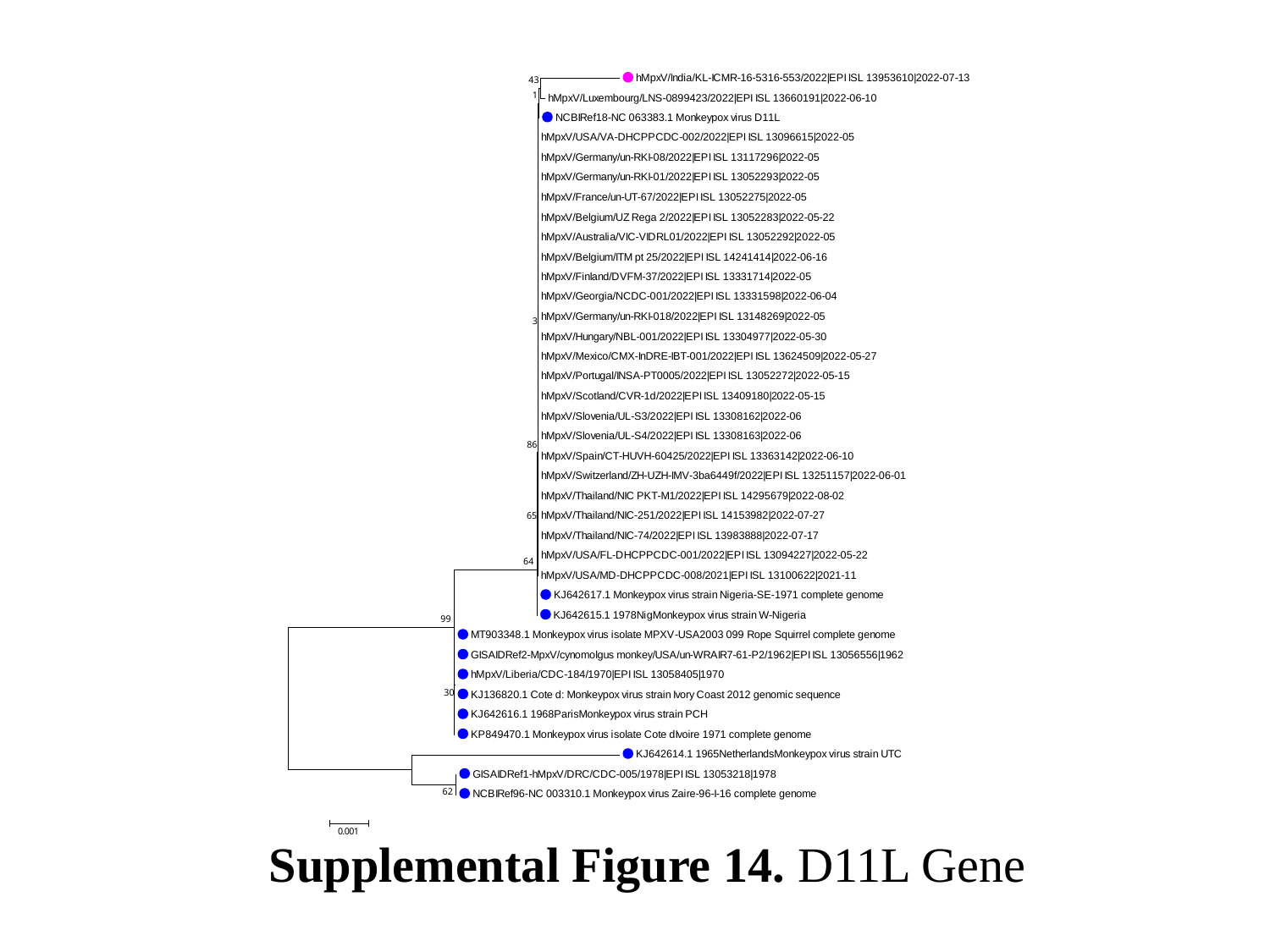

# Supplemental Figure 14. D11L Gene

## Slide 20
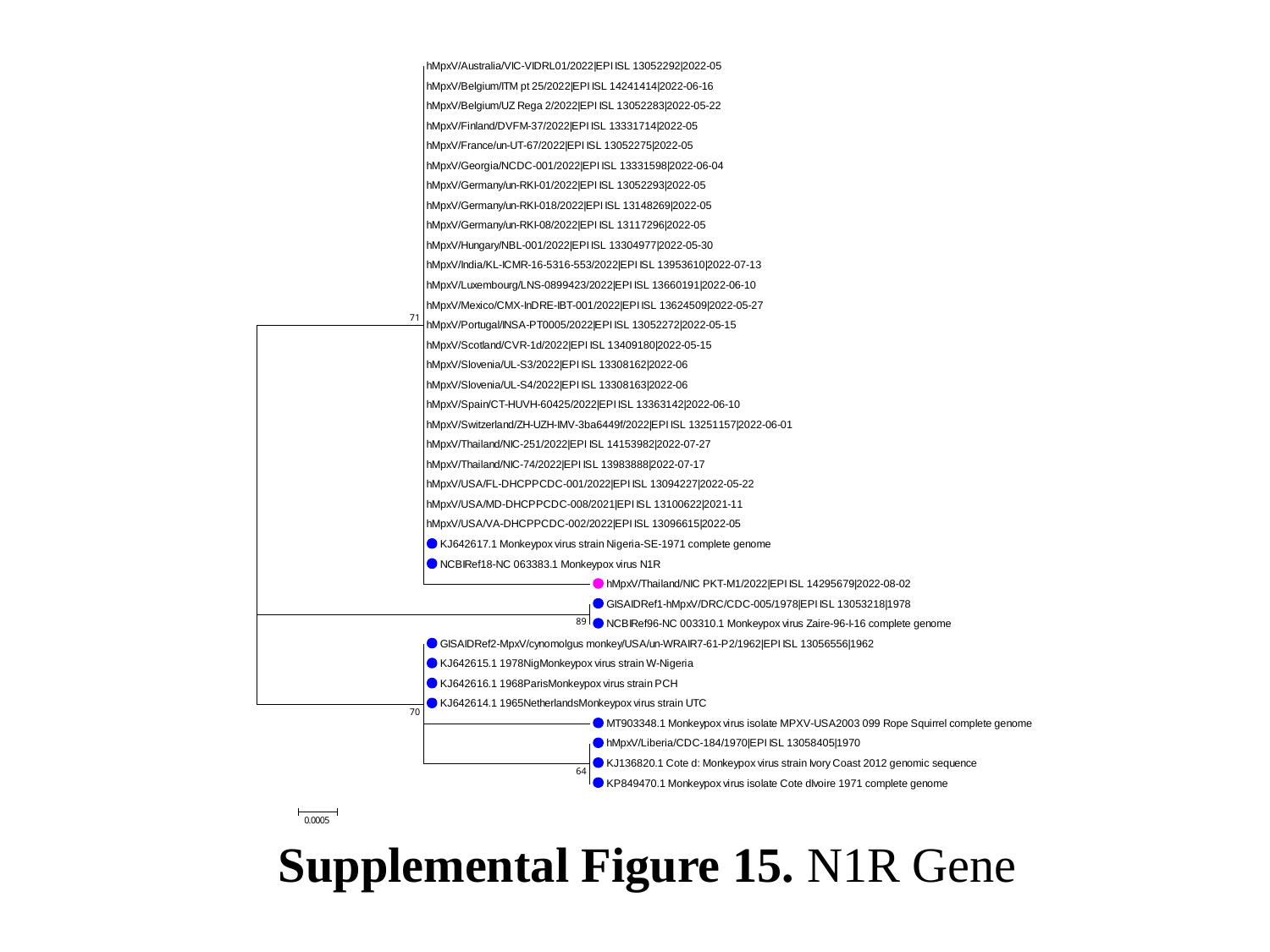

# Supplemental Figure 15. N1R Gene

## Slide 21
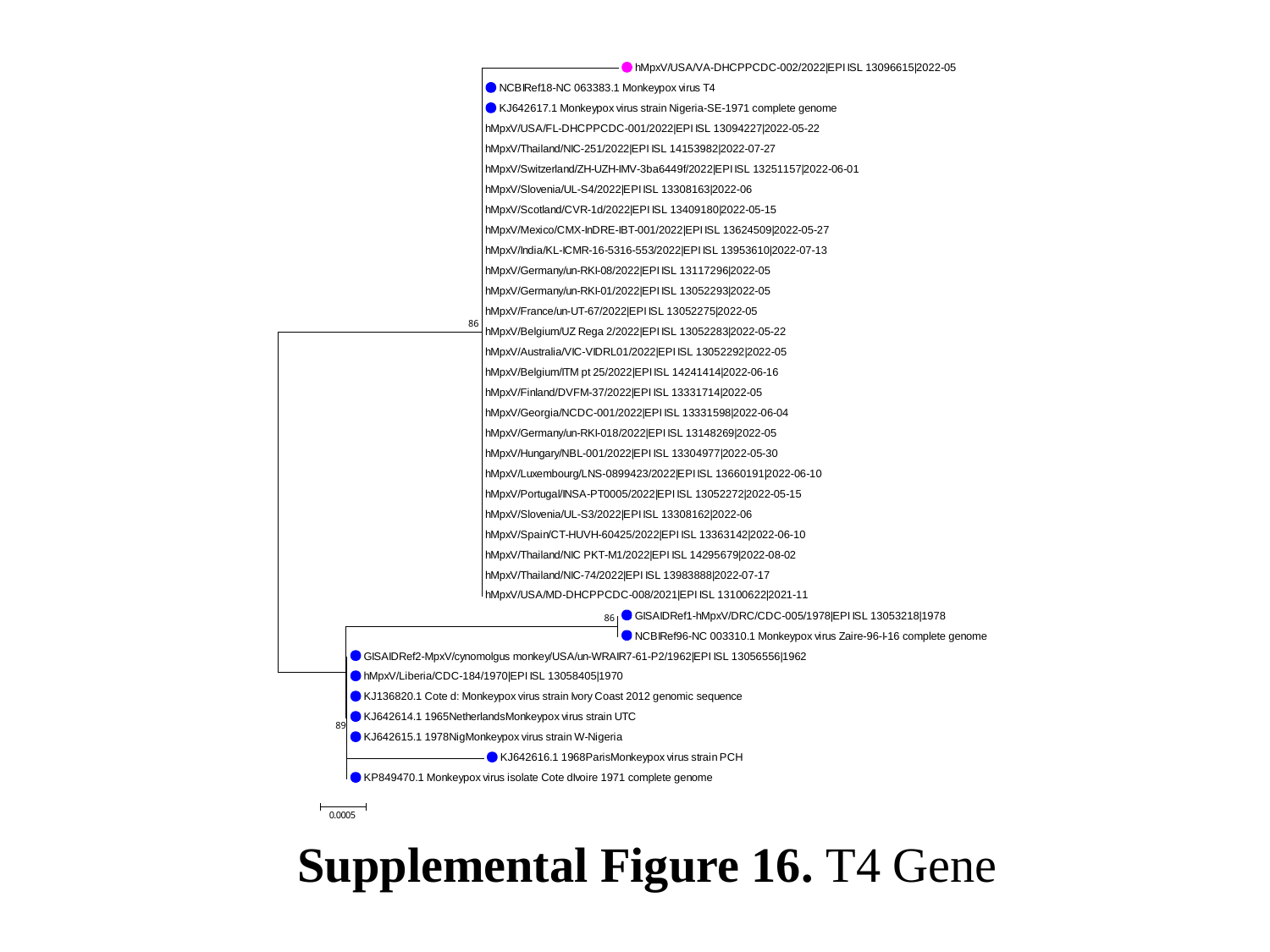

# Supplemental Figure 16. T4 Gene

## Slide 22
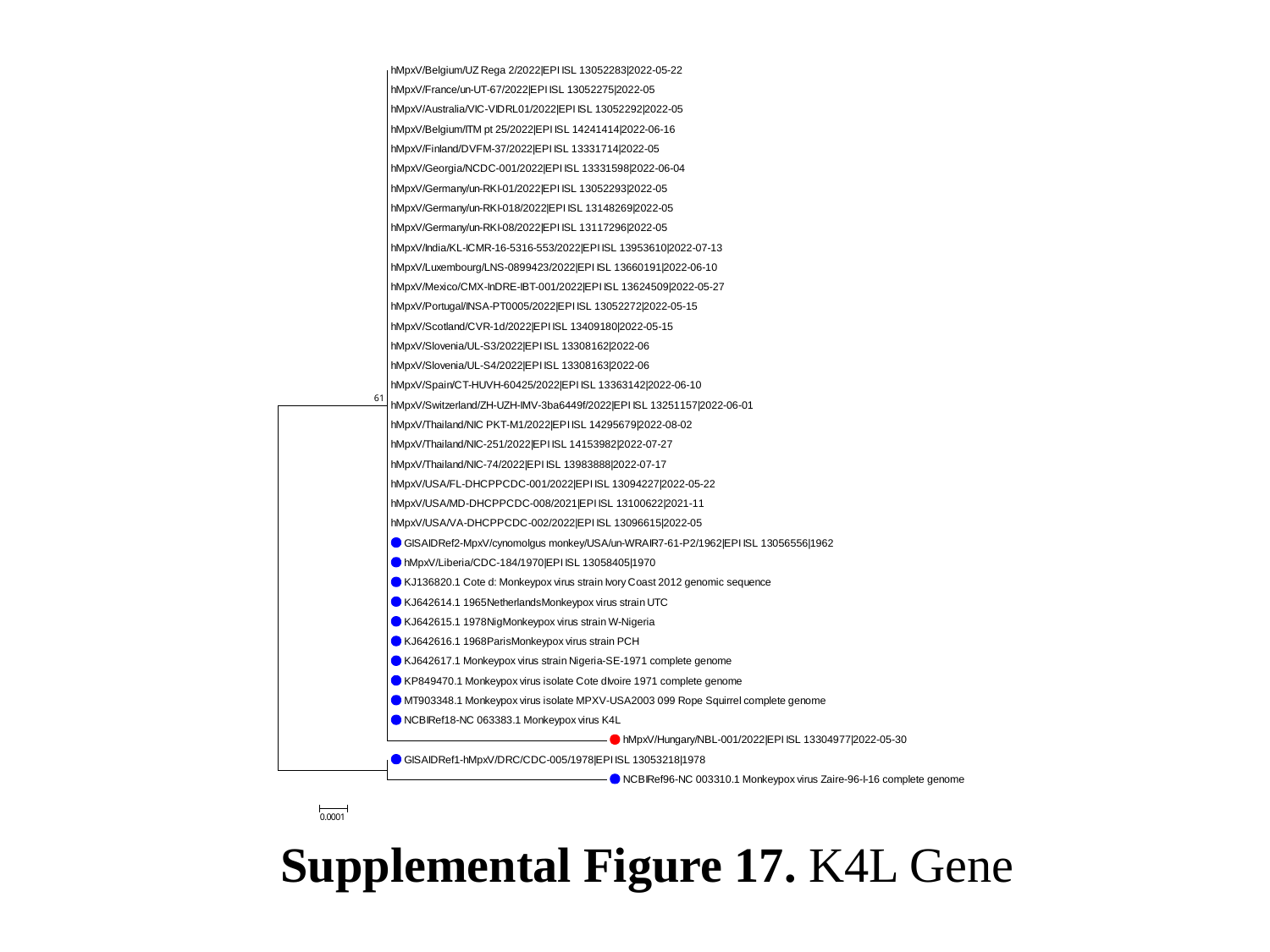

# Supplemental Figure 17. K4L Gene

## Slide 23
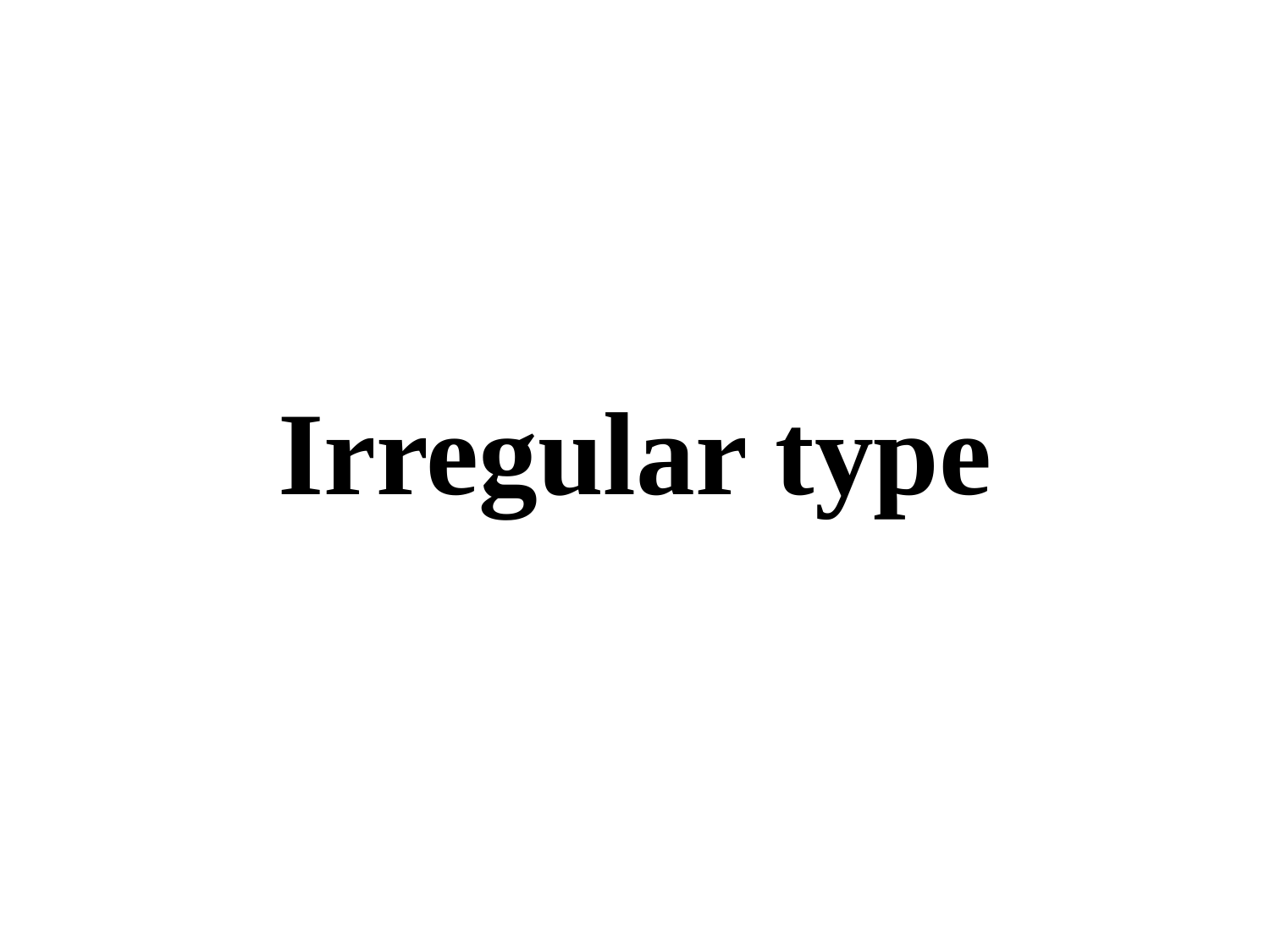

# Irregular type

## Slide 24
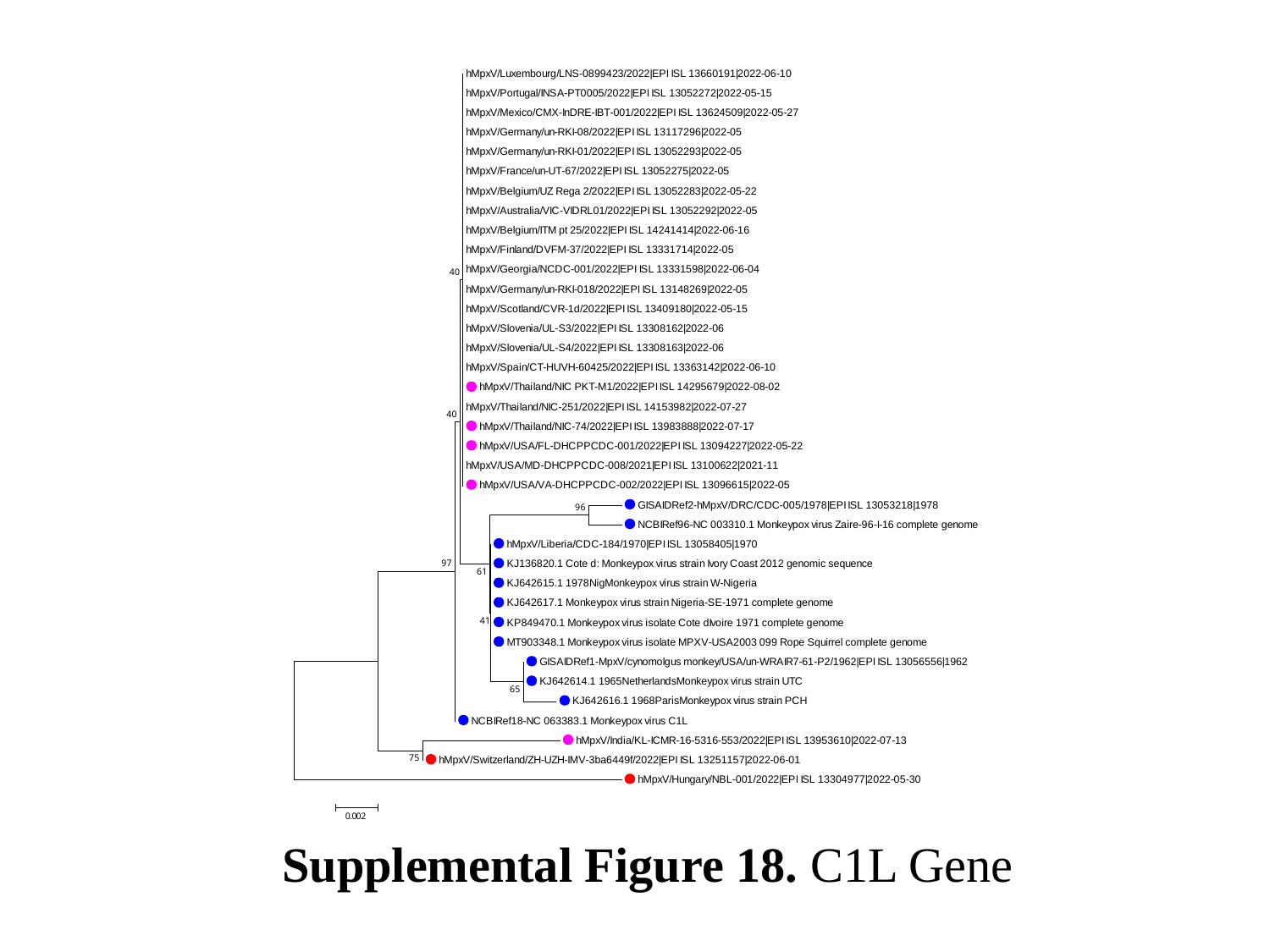

# Supplemental Figure 18. C1L Gene

## Slide 25
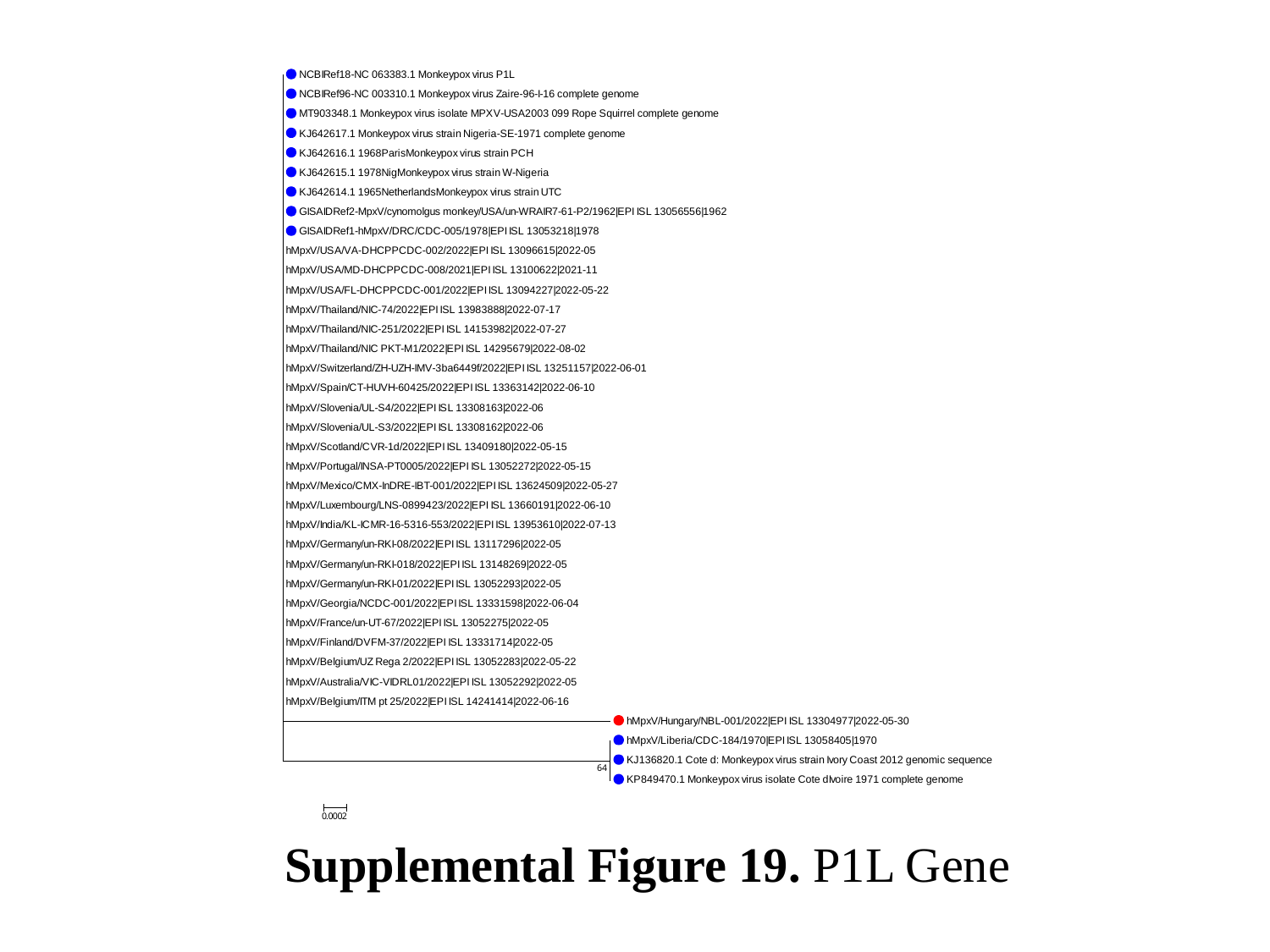

# Supplemental Figure 19. P1L Gene

## Slide 26
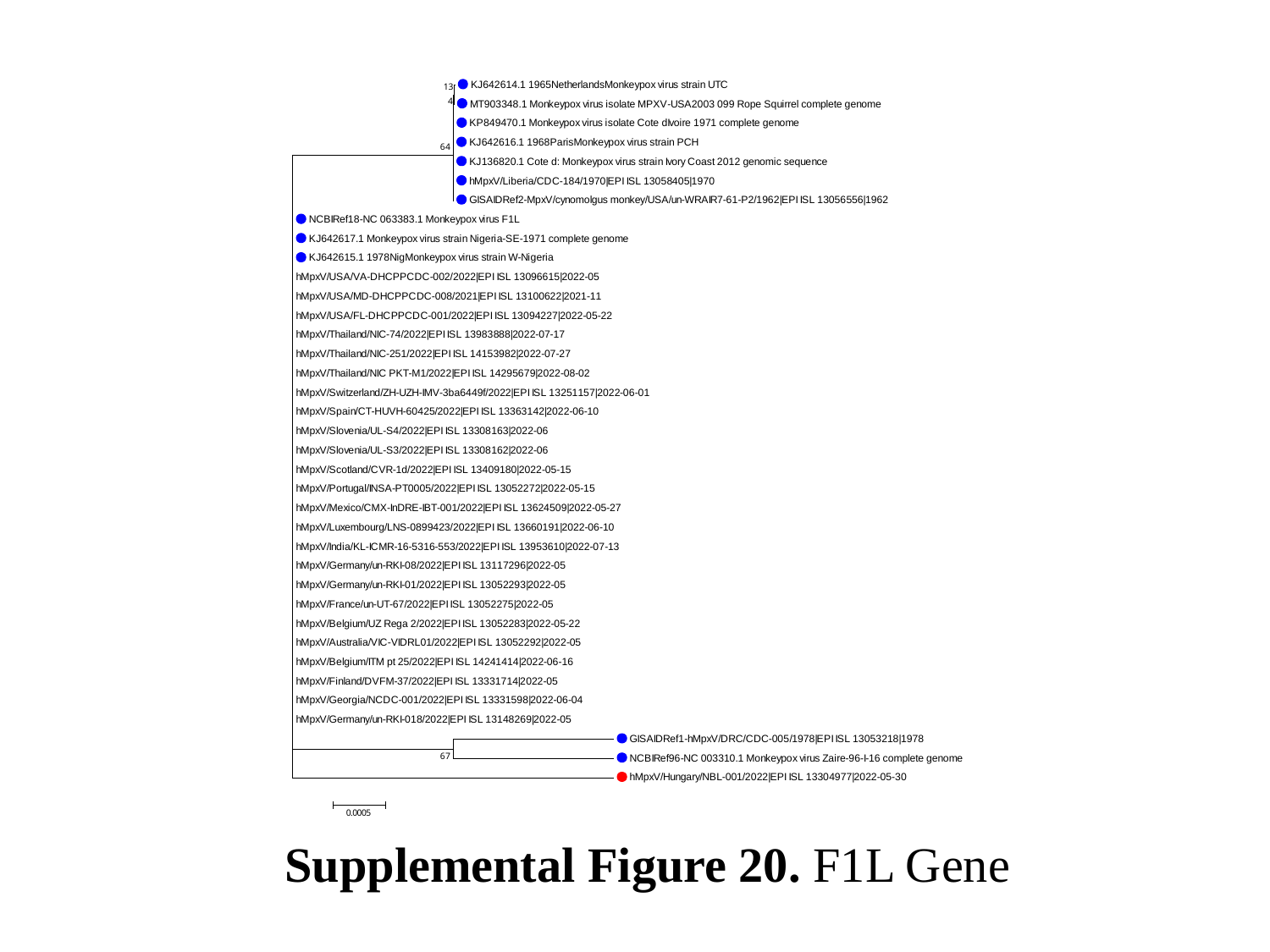

# Supplemental Figure 20. F1L Gene

## Slide 27
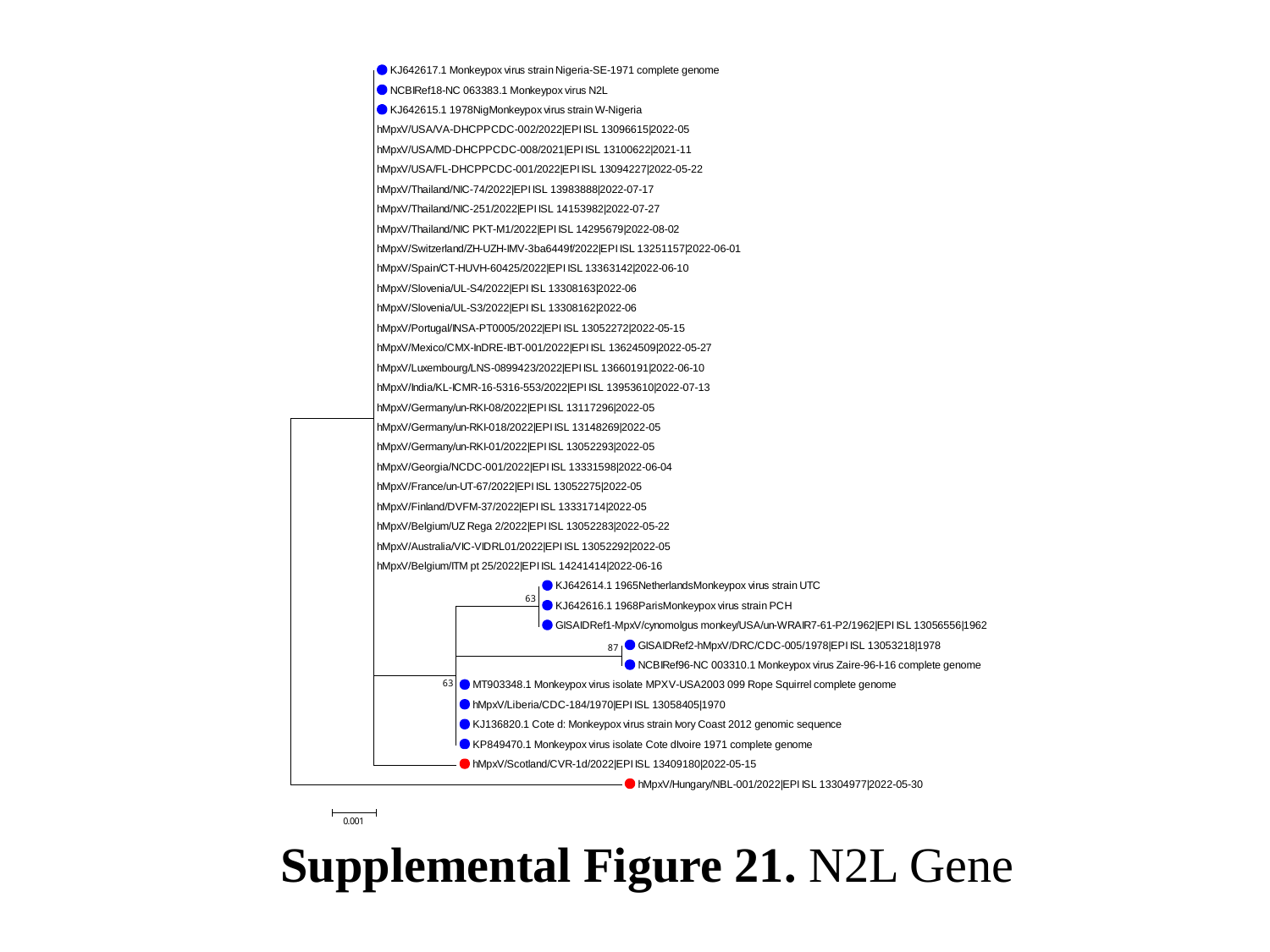

# Supplemental Figure 21. N2L Gene

## Slide 28
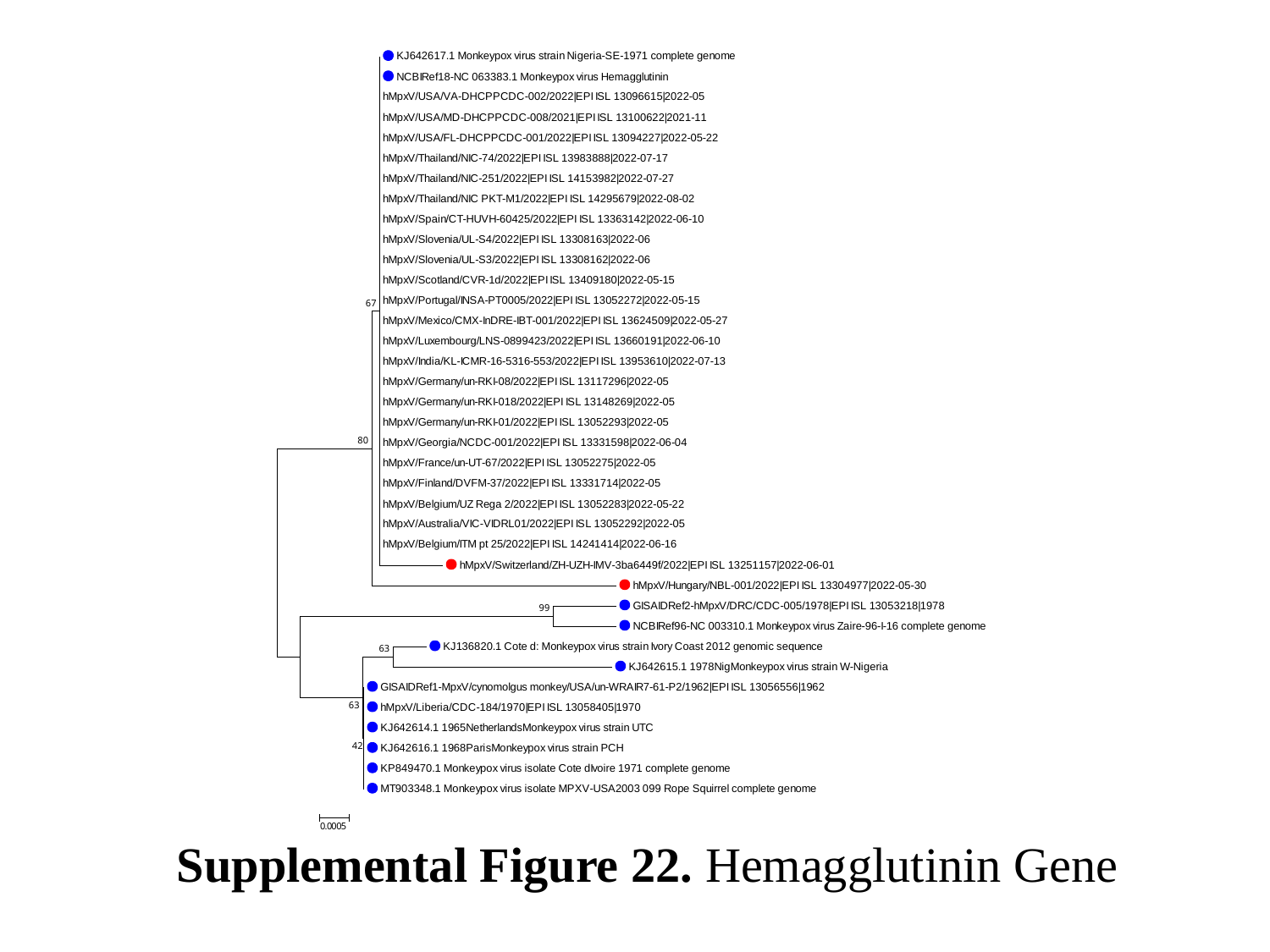

# Supplemental Figure 22. Hemagglutinin Gene

## Slide 29
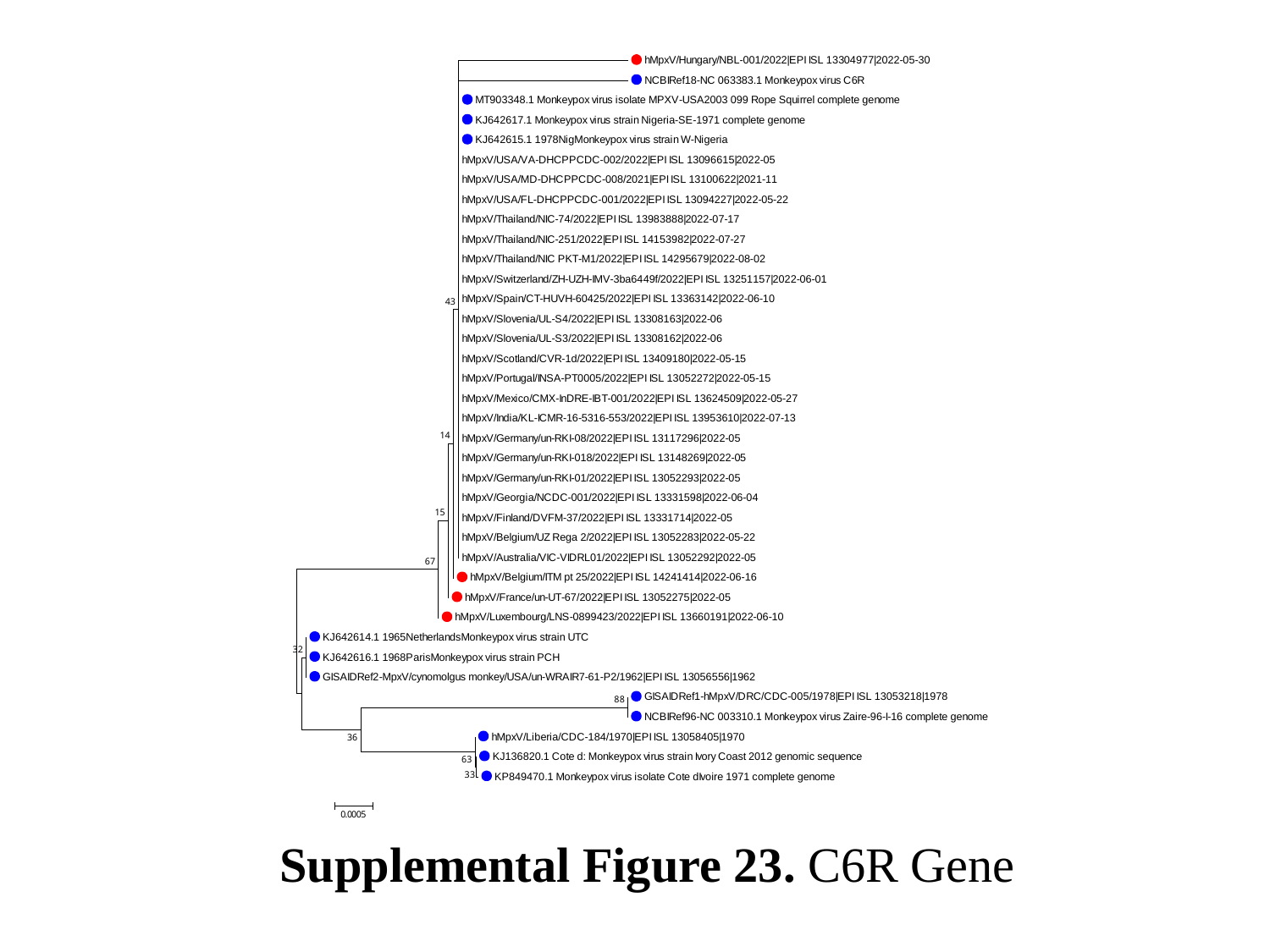

# Supplemental Figure 23. C6R Gene

## Slide 30
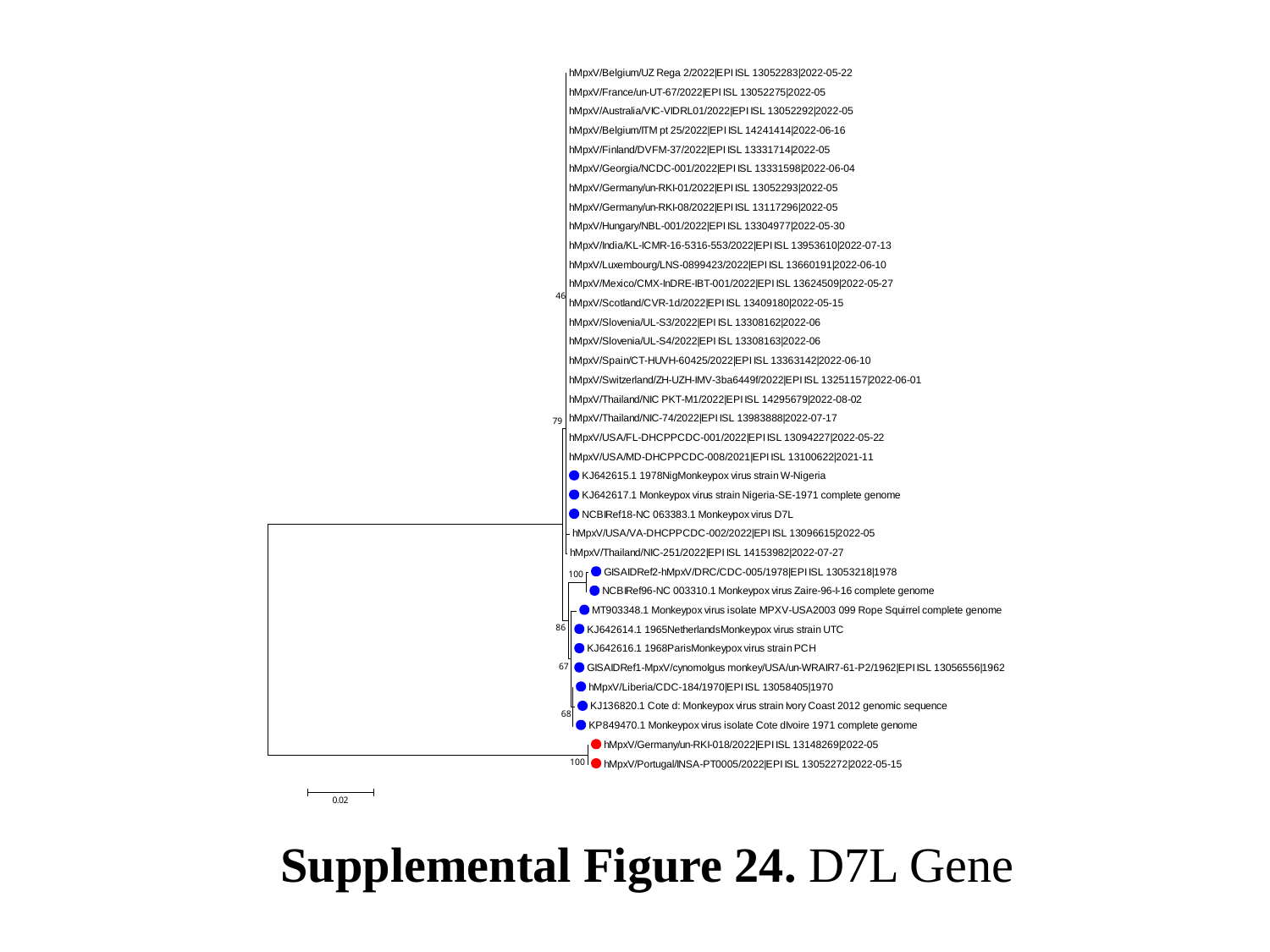

# Supplemental Figure 24. D7L Gene

## Slide 31
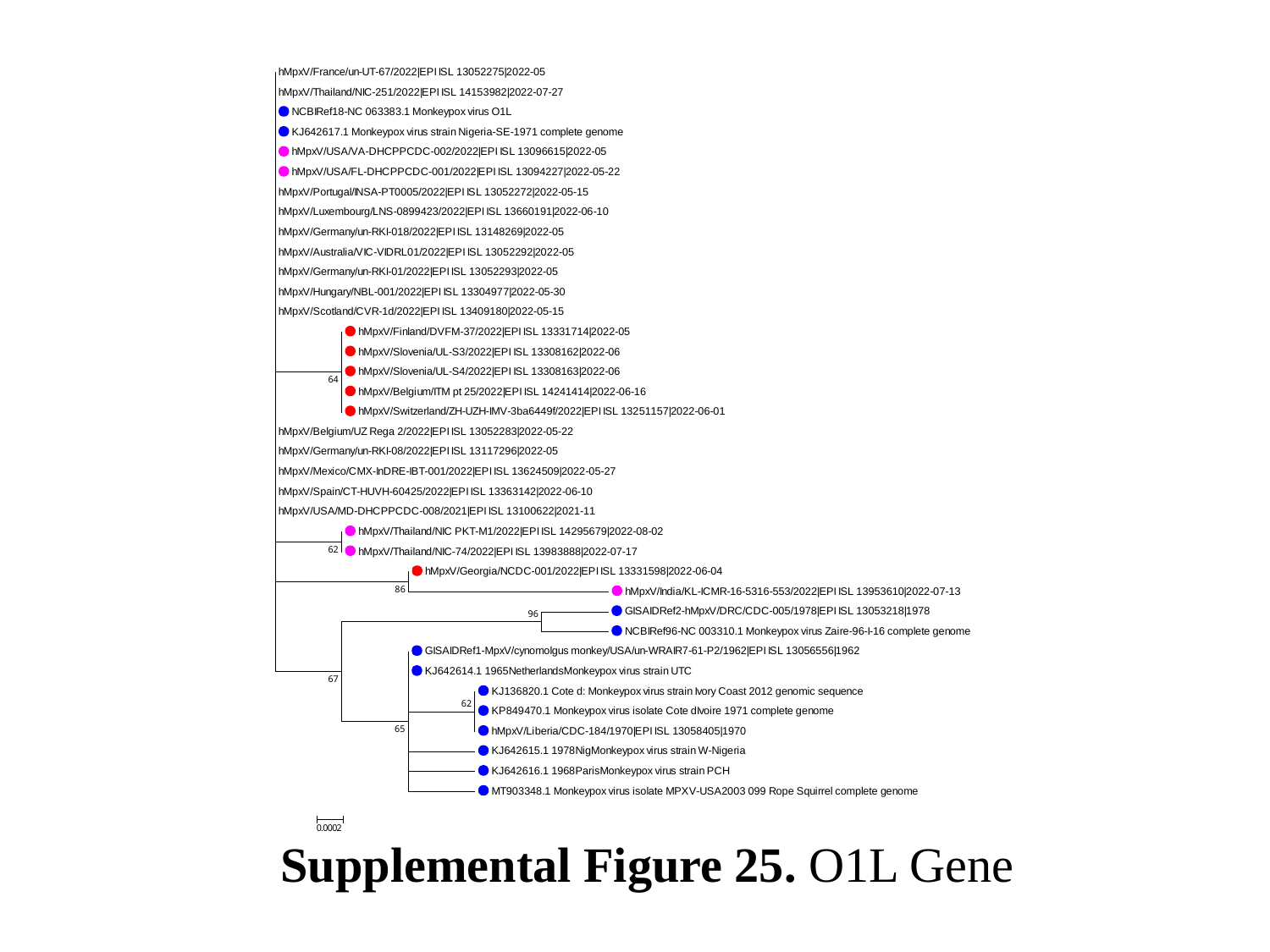

# Supplemental Figure 25. O1L Gene
